# Active HHV-6 Infection of Cerebellar Purkinje Cells in Mood Disorders

**DOI:** 10.3389/fmicb.2018.01955

**Published:** 2018-08-21

**Authors:** Bhupesh K. Prusty, Nitish Gulve, Sheila Govind, Gerhard R. F. Krueger, Julia Feichtinger, Lee Larcombe, Richard Aspinall, Dharam V. Ablashi, Carla T. Toro

**Affiliations:** ^1^Biocenter, Department of Microbiology, University of Würzburg, Würzburg, Germany; ^2^Institute for Virology and Immunobiology, University of Würzburg, Würzburg, Germany; ^3^Division of Virology, National Institute for Biological Standards and Control, Hertfordshire, United Kingdom; ^4^Department of Pathology and Laboratory Medicine, UT-Houston Medical School, Houston, TX, United States; ^5^Institute of Computational Biotechnology, Graz University of Technology, Graz, Austria; ^6^BioTechMed Omics Center, Graz, Austria; ^7^Applied Exomics Ltd., Stevenage Bioscience Catalyst, Stevenage, United Kingdom; ^8^Faculty of Health and Life Sciences, Coventry University, Coventry, United Kingdom; ^9^HHV-6 Foundation, Santa Barbara, CA, United States; ^10^The Institute of Digital Healthcare, The University of Warwick, Warwick, United Kingdom

**Keywords:** HHV-6, bipolar disorder, schizophrenia, major depressive disorder, Purkinje cells

## Abstract

Early-life infections and associated neuroinflammation is incriminated in the pathogenesis of various mood disorders. Infection with human roseoloviruses, HHV-6A and HHV-6B, allows viral latency in the central nervous system and other tissues, which can later be activated causing cognitive and behavioral disturbances. Hence, this study was designed to evaluate possible association of HHV-6A and HHV-6B activation with three different groups of psychiatric patients. DNA qPCR, immunofluorescence and FISH studies were carried out in post-mortem posterior cerebellum from 50 cases each of bipolar disorder (BPD), schizophrenia, 15 major depressive disorder (MDD) and 50 appropriate control samples obtained from two well-known brain collections (Stanley Medical Research Institute). HHV-6A and HHV-6B late proteins (indicating active infection) and viral DNA were detected more frequently (*p* < 0.001 for each virus) in human cerebellum in MDD and BPD relative to controls. These roseolovirus proteins and DNA were found less frequently in schizophrenia cases. Active HHV-6A and HHV-6B infection in cerebellar Purkinje cells were detected frequently in BPD and MDD cases. Furthermore, we found a significant association of HHV-6A infection with reduced Purkinje cell size, suggesting virus-mediated abnormal Purkinje cell function in these disorders. Finally, gene expression analysis of cerebellar tissue revealed changes in pathways reflecting an inflammatory response possibly to HHV-6A infection. Our results provide molecular evidence to support a role for active HHV-6A and HHV-6B infection in BPD and MDD.

## Introduction

Heritable as well as environmental factors, particularly those that lead to neuroinflammation early in life, might play an important etiologic role in bipolar disorder (BPD), major depressive disorder (MDD), and schizophrenia (SCZ) (Schmitt et al., 2014). Prenatal maternal infection with influenza type A, *Toxoplasma gondii*, measles or rubella increases disease risk in SCZ (Hagberg et al., 2012). Prenatal exposure to influenza also leads to a fourfold increase in the risk of developing BPD (Parboosing et al., 2013) and a meta-analysis of MDD reported association with Borna disease virus and members of the herpesvirus family HSV-1, HHV-3, and HHV-4 (Wang et al., 2014). Childhood hospitalizations for viral and non-viral infection are associated with an increased risk of developing SCZ, MDD, and BPD (Khandaker et al., 2012; Benros et al., 2013). Candidate pathogens may disrupt neurodevelopment and cross talk with the immune system at key developmental stages.

The roseolovirus genus of betaherpesviruses consisting of human herpesvirus (HHV) HHV-6A, HHV-6B, and HHV-7 infect a large fraction of the human population, leading to viral latency in various organs/tissues including the central nervous system (CNS), salivary glands, accompanied by periodic reactivation (Agut et al., 2015). Although HHV-6A and HHV-6B are known to be neurotropic, signs of active infection, i.e., the presence of late proteins expressed immediately after the replicative cycle–have not previously been investigated in brain tissue in psychiatric illness (Luppi et al., 1994; Challoner et al., 1995; Chan et al., 2001; Cuomo et al., 2001; Esposito et al., 2015).

We studied infection of HHV-6A and HHV-6B by both quantitative PCR-based DNA analysis and immunofluorescence (IFA) assays in post-mortem human cerebellum samples from patients with SCZ, BPD, MDD, and comparison controls (CON). The cerebellum was chosen based on evidence from animal models that perinatal viral infection or immune activation with viral mimetic leads to neuroanatomical changes in the cerebellum, and behavioral disturbances in adulthood (Williams et al., 2007; Shi et al., 2009; Aavani et al., 2015; da Silveira et al., 2017). Furthermore, altered cerebellar function is associated with psychiatric disorders (reviewed in Phillips et al., 2015) including abnormal corticocerebellar connections in SCZ (Ueland et al., 2004; Laidi et al., 2015), decreased cerebellar volume and cerebellar atrophy in BPD (Andreasen et al., 1996; Baldacara et al., 2011) and smaller cerebellum size in MDD patients (Brambilla et al., 2002). Here we report molecular evidence for active HHV-6A and HHV-6B infection in Purkinje cells of human cerebellum in BPD and MDD.

## Materials and Methods

### Post-mortem Brain Samples

Human posterior cerebellum tissue samples were obtained from two collections housed at the Stanley Medical Research Institute, approved by NHS Research Ethics Committee (09/H0603/18). The Neuropathology Consortium collection included 60 samples from 15 SCZ, 15 MDD, 15 BPD and 15 non-psychiatric, non-neurological CON, matched for age, post-mortem interval (PMI), brain pH, gender, and side of brain (Torrey et al., 2000). The Array collection consisted of 105 samples from 35 SCZ, 35 BPD and 35 non-psychiatric, non-neurological CON^1^. The median age and cause of death of both the cohort of samples are presented in **Supplementary Tables S1, S2**, respectively.

Frozen blocks of cerebellum were used for quantitative real-time PCR (qPCR), immunofluorescence analysis (IFA), and transmission electron microscopy (TEM). Formalin-fixed, paraffin-embedded (FFPE) sections (10 μm) were used for fluorescent *in situ* hybridization (FISH).

### Immunofluorescence Analysis (IFA)

To detect cell-specific infection by HHV-6A or HHV-6B, we examined the presence of HHV-6A and HHV-6B late proteins, gp82/105 and OHV3 respectively, as a marker of active viral infection, using IFA staining in both cohorts. When IFA staining indicated possible infection with HHV-6A and/or HHV-6B, tropism was verified using two further HHV-6-specific antibodies [HHV-6B specific U94 and glycoprotein B (gB) of both HHV-6A and HHV-6]. Positive samples were crosschecked for virus-specific staining using confocal microscopy. Presence of HHV-6 DNA was confirmed by FISH analysis and active viral infection was verified by TEM in randomly selected samples. Antibody details are provided in **Supplementary Table S3**.

To determine the cell type(s) infected with HHV-6A and/or HHV-6B, co-staining with Purkinje cell specific marker RBFOX2, astrocyte specific marker GFAP and microglia specific marker Iba1 were used. Antibodies against NeuN were used to identify other neurons such as granule cells. All experiments were carried out blind to diagnosis. Antibody specificity against HHV-6A or HHV-6B was verified using cultured cells infected either with HHV-6A (U1102) or HHV-6B (Z29) (**Supplementary Figure S1**).

Frozen (14 μm) cerebellum sections (posterior lobe) were fixed for 15 min in ice cold methanol and acetone (1:1), followed by permeabilization in 0.2% Triton X-100 for 20 min at room temperature (RT). Sections were treated with 0.4% pepsin for 30 min at 37°C. Slides were rinsed with PBS and blocked for 30 min in 10% fetal calf serum (FCS) followed by incubation with primary antibodies (**Supplementary Table S3**) in 2% FCS for 1 h at RT. After washes in 1X PBS, sections were incubated in respective secondary antibodies in 2% FCS containing DAPI. After washes, sections were air-dried and mounted with anti-fade medium containing *p*-Phenylenediamine (P-6001, Sigma).

Two brain sections from each sample were stained for each viral antibody (HHV-6A gp82/105, HHV-6B OHV3) on a separate day together with a marker for neuronal cells (NeuN). Additional staining for HHV-6B U94 and HHV-6A and/or HHV-6B gB was carried out for cohort 1 (*n*= 60) to confirm specificity for Purkinje cells. Specificity for all antibodies against HHV-6A and HHV-6B has been previously verified (Takeda et al., 1996; Dhepakson et al., 2002; Mori et al., 2002; Santoro et al., 2003; Loginov et al., 2010; Pfeiffer and Lingner, 2012). In addition, we confirmed specificity of the antibodies using HHV-6A (U1102) and HHV-6B (Z29) infected HSB-2 and Molt-3 cells respectively (Das et al., 2016). All the samples showing possible HHV-6 positivity (*n*= 20) in cells other then Purkinje cells were stained using anti-GFAP (astrocyte specific staining) and anti-Iba1 (microglia specific staining) monoclonal antibodies. Samples were analyzed on a Leica DMR epifluorescence microscope. For confocal imaging samples were analyzed on a Leica SP5 or SPE confocal microscope. All image-processing steps were performed using FIJI (Schindelin et al., 2012). Image background was subtracted using the rolling ball background subtraction. All image analyses were carried out blind to diagnosis.

To avoid selection bias due to staining artifacts, stringent criteria were applied. Samples were considered positive for HHV-6A/B infection, when more than three HHV-6A/B positive Purkinje cells were detected within a region of interest in at least two of the five areas assessed per section. Well-defined cytoplasmic or nuclear signal of viral proteins were considered essential for classification of positive samples.

### Fluorescent *in situ* Hybridization (FISH)

The FISH assay was designed to detect HHV-6 using fluorescent PCR-probes with no differentiation between HHV-6A and HHV-6B types. FFPE sections (10 μm) of cerebellum and orbitofrontal cortex (OFC) were baked overnight (12–18 h) at 55°C then rinsed using xylene, dehydrated in 100% ethanol and air-dried. Subsequently slides were incubated in 0.2N HCl at RT for 20 min, rinsed in water then incubated in pre-warmed 1N NaSCN solution at 80°C. After rinsing, sections were treated with 0.4% pepsin for 10 min at 37°C, rinsed with PBS and incubated in 10% buffered formalin for 15 min at RT. Slides were washed and hybridized for 12–18 h in a humidified environment at 37°C with fluorophore-tagged HHV-6 U22 or U42 PCR probes in hybridization buffer containing 10 mM Tris-HCl (pH 7.2, 70% Formamide). Slides were washed in 2X SSC/0.1% Tween 20 and 0.5X SSC/0.1% Tween 20 at 65°C, then mounted using DAPI/Antifade solution.

Cy3-tagged PCR products were generated from HHV-6A (U1102) infected cellular DNA using Cy3 PCR Labeling Kit (Jena Biosciences, Germany) using the following primer pairs. U22_For 5′-GGATCCAAAGCAAACCAGCAAGA-3′, U22_Rev 5′-TGGCGGATGGCTAGTGTGCC-3′, U42_For 5′-AGTTAGTTTCACAGGTGTCAGC-3′, and U42_Rev 5′-ACCGAAATCTTTCTTTTACTTGTC-3′. PCR amplified DNA were gel eluted, purified, and used for FISH.

### Transmission Electron Microscopy (TEM)

Transmission electron microscopy was carried by directly fixing sections of cerebellum (14 μm thick) with 2.5% glutaraldehyde (50 mM sodium cacodylate pH 7.2; 50 mM KCl; 2.5 mM MgCl_2_) at RT, incubated for 2 h at 4°C with 2% OsO_4_ buffered with 50 mM sodium cacodylate (pH 7.2), washed with dH_2_O and incubated overnight at 4°C with 0.5% uranyl acetate (in dH_2_O). Sections were dehydrated, embedded in Epon812 and ultrathin sectioned at 50 nm. Sections were stained with 2% uranyl acetate in ethanol, followed by staining with lead citrate and analyzed on a Zeiss EM10 (Zeiss, Oberkochen, Germany). Two independent pathologists reviewed all sections.

### DNA Extraction From Frozen Blocks of Tissue

Genomic DNA was extracted from frozen (40–50 mg) blocks of cerebellum using a method described previously (Dunham et al., 2009). DNA pellets were re-suspended in TE buffer (10 mM Tris; 1 mM EDTA, pH 8) overnight, then samples normalized to 50 ng/μl following quantification and verification of integrity (Picodrop 260/280 ratio > 1.8), and frozen at -20°C.

### Quantitative Real Time PCR (qPCR)

Total DNA isolated from frozen samples was quantitated using previously described qPCR assays (Prusty et al., 2013b, 2017; Gulve et al., 2017). DNA samples (100 ng per sample) were amplified using PerfeCTa qPCR SuperMix (Quanta Biosciences) and StepOnePlus real time PCR platform (Applied Biosciences) and SYBR Green chemistry. DNA derived from HeLa cells carrying two copies of latent and integrated viral genome (Gulve et al., 2017) was used as positive control. DNA from HeLa cells without having HHV-6A or HHV-6B served as negative control. In contrast to IFA, FISH and TEM results–which focused on infection of specific cell types–the qPCR measurements involved the entire cerebellar sample.

### Preparation of Plasmids for Standard Curve Analysis

HHV-6A U94 and human PI15 ORF cloning and standard curve analysis are previously described (Prusty et al., 2013b). HHV-6A and HHV-6B DR specific PCR is described elsewhere (Gulve et al., 2017). Limits of detection (LOD) for HHV-6 U94, PI15, and HHV-6A DR were 10 copies per reaction and that for HHV-6B DR was one copy per reaction with a CV value of less than 5%. Ten randomly selected PCR products from each PCR were verified by sequencing. Samples were considered as negative for HHV-6A or HHV-6B by qPCR when the detected numbers of viral genome equivalents were less than the LOD in a particular qPCR reaction.

### Measurement of Soma Size of Purkinje Cells

Purkinje cells were identified using RBFOX2 staining from fluorescence microscopy images. Only large round cells localized between the granular and molecular layers and where the border between somatic and proximal dendritic membrane could be defined, were selected for measurement of cross-sectional soma area (Fatemi et al., 1999; Sakamoto et al., 2001). In total between 10 and 20 labeled Purkinje cells were measured from randomly chosen fields of view for each case, using ImageJ software.

### Gene Expression (Microarray) Analysis

Differential gene expression analysis of the cerebellum in 116 cases from the Array and Consortium collections used in this study has been conducted previously (Torrey et al., 2000; Chen et al., 2013).

The raw data of the dataset E-GEOD-35974 (Chen et al., 2013) (144 samples, Affymetrix GeneChip Human Gene 1.0 ST Arrays) was downloaded from ArrayExpress and analyzed in R 3.3.2 (R Development Core Team, 2017). Based on our studies of the same tissue, we compared the gene expression in those subjects we classed as HHV-6A positive for both DNA and protein to those we classified as HHV negative for both DNA and protein, with diagnosis, age and post-mortal interval (PMI) as covariates. Then, we performed pathway analysis based on a gene set enrichment analysis (GSEA).

The R package ‘oligo’ (Carvalho and Irizarry, 2010) was used to preprocess the arrays using rma as well as for quality control. Replicates, bad quality arrays and outliers based on PCA as well as samples without HHV-6A or HHV-6B infection status were removed, resulting in 116 arrays for further analysis. Non-specific filtering was applied to remove low expressed genes before proceeding with the analysis. The R package ‘limma’ (Ritchie et al., 2015) was used to compute log2 (fold changes) between the groups (HHV-6 infected vs. non-infected) with diagnosis, age, and PMI as covariates. The computed log2 (fold changes) were fed into a GSEA using the R package ‘ReactomePA’ (Yu and He, 2016) and Reactome pathways with a *q*-value <= 0.05 were considered as significantly enriched.

### Statistical Analysis

Nominal HHV-6A and HHV-6B data was analyzed using the Pearson’s Chi-square test for more than two diagnostic groups, whereas the Fisher’s exact test (two-sided) was applied for pair-wise analysis. Scale data for HHV-6A and HHV-6B was analyzed using Univariate ANOVA and Bonferroni *post hoc* test for pair-wise analysis. Covariates were defined by assessing the effect of potentially confounding variables (age, PMI, pH, age of illness onset, duration of illness) using Spearman’s rho correlation analysis. Additional between-subjects variables were included in Univariate ANOVA if found to be significant when assessed individually using one-way ANOVA. Viral copy number data was analyzed using Kruskal–Wallis test to reveal differences between diagnostic groups. Multinomial logistic regression was used to assess gender and diagnosis with HHV-6A and HHV-6B protein variables. All statistical analyses were performed using SPSS (version 22).

## Results

### HHV-6A and HHV-6B Active Infection of Purkinje Cells

**FIGURE 1:**
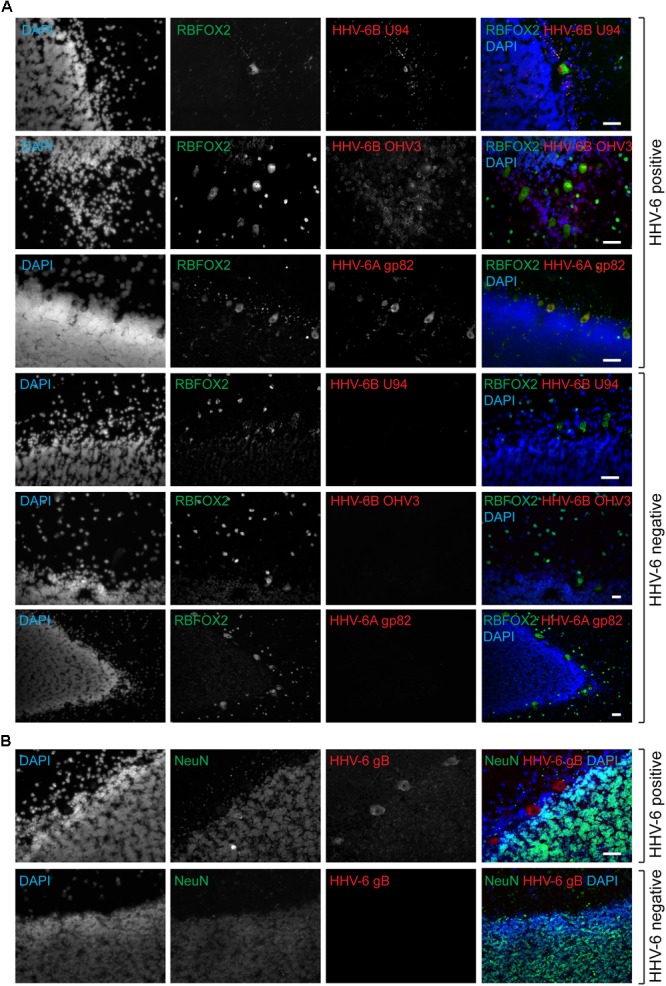
Active HHV-6 infection in Cerebellum of mood disorder patients. **(A)** Representative images showing Immuno-fluorescence analysis for HHV-6 viral antigens in cerebellar cortex samples. Cryo-sectioned cerebral cortex samples were stained using antibodies raised against different HHV-6 antigens (gp82/105, U94, and OHV3) together with either RBFOX2 (Fox2; marker for Purkinje cells). Representative samples without HHV-6 infection (negative control) are also shown in the lower panel. **(B)** Purkinje cell specific HHV-6 staining was tested using antibodies against NeuN (marker for other neuronal cells). Each panel shows results from a different case. DAPI was used to stain DNA. The scale bars (indicated with white lines) represent 200 μm.

**FIGURE 2:**
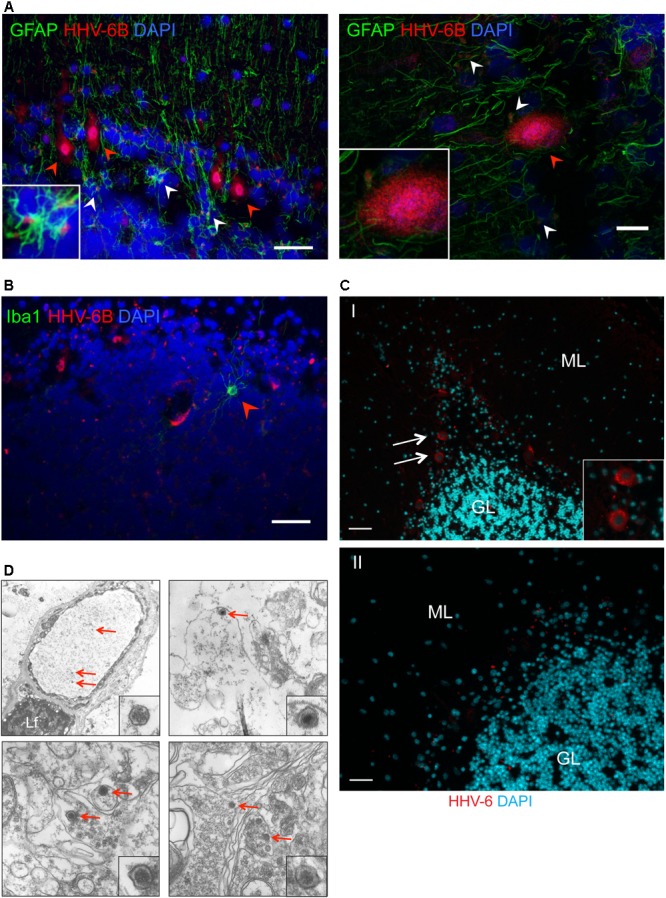
Detection of HHV-6 in Purkinje cells and other associated cell types. **(A)** Immuno-fluorescence analysis to study HHV-6 infection in astrocytes within cerebellar cortex samples. Cryo-sectioned cerebellar cortex samples were co-stained using monoclonal antibodies against OHV3 together with GFAP (marker for astrocytes). Left panel shows a representative image taken with epifluorescence microscope whereas the right panel shows a representative image taken with confocal microscope. HHV-6 positive Purkinje cells are marked with red arrowheads. HHV-6 positive astrocytes are marked with white arrowheads. **(B)** Immuno-fluorescence analysis showing status of HHV-6 infection in microglial cells within cerebellar cortex samples. Cryo-sectioned cerebellar cortex samples were co-stained using monoclonal antibodies against OHV3 together with Iba1 (marker for microglia). Iba1 positive microglial cells are marked with red arrowheads. **(C)** FISH analysis in FFPE samples of cerebral cortex region detected HHV-6 positive Purkinje cells (marked with white arrows). ML, molecular layer; GL, granular layer. Panel I shows a representative image of positive staining for HHV-6 while panel II shows a representative image for negative staining for HHV-6. The scale bars represent (indicated with white lines) 200 μm. **(D)** Transmission electron microscopy was used to identify HHV-6 viral particles (marked with red arrows) in randomly selected samples positive for HHV-6 by both qPCR and IFA. Lf, lipofuscin.

Quantitative PCR analysis for HHV-6A and HHV-6B infection was carried out in both cohorts of samples (**Supplementary Tables S4, S5**). No significant difference in viral loads was observed between psychiatric cases and controls. Subsequently, immunofluorescence studies were carried out using samples from both cohorts (**Supplementary Table S6**). We detected HHV-6A and HHV-6B infection predominantly in Purkinje cells at the interface between the molecular and granular layers (**Figures 1A,1B** and **Supplementary Figure S2**). We detected putative staining of other smaller cells in 20 samples; of these, we detected GFAP-positive astrocytes as HHV-6B positive in 16 cases (**Figure 2A** and **Supplementary Figures S3, S4**). All cases showing HHV-6B positivity of astrocytes were also positive for HHV-6B in local Purkinje cells. Occasionally, Iba1-positive microglial cells (*n*= 4) also positively stained for either HHV-6A or -6B (**Figure 2B** and **Supplementary Figure S3**) in samples carrying HHV-6A/B infection in Purkinje cells.

Presence of viral infection of Purkinje cells was verified by FISH (*n*= 25) and TEM (*n*= 7) in randomly selected HHV-6A or HHV-6B positive and negative cases. FISH analysis confirmed specific localization of HHV-6A/B DNA in Purkinje cells (**Figures 2C,D**). FISH and TEM analysis revealed a negative signal in all HHV-6A/B DNA/protein negative samples we examined.

### Association of HHV-6A and HHV-6B Infection With Mood Disorders

As summarized in **Table 1** and **Supplementary Table S6**, late proteins and DNA from both HHV-6A and HHV-6B were found in Purkinje cells significantly more often in patients with MDD than in matched controls without having neurological or psychiatric disease. The combination of both late proteins and DNA, from both HHV-6A and HHV-6B, was found significantly more often in patients with BPD than in CON. In contrast, there was no difference between patients with SCZ and CON with regards to the detection of late proteins or DNA from either virus.

**Table 1:** Detection frequencies for HHV-6A and HHV-6B protein and DNA in cerebellum from cohort 1 and cohort 2 combined, in the three disease groups and in controls.

	*p*-Values^∗^						
	MDD	BPD	SCZ	CON	MDD vs. CON	BPD vs. CON	SCZ vs. CON
HHV-6A protein presence (%)	11/15 (73%)	23/49 (47%)	15/50 (30%)	11/50 (22%)	<0.0001	0.01	0.50
HHV-6A DNA presence (%)	13/15 (87%)	27/49 (55%)	27/48 (56%)	23/50 (46%)	0.007	0.42	0.32
HHV-6A protein and DNA presence (%)	10/15 (67%)	15/49 (31%)	6/48 (13%)	2/50 (4%)	<0.0001	<0.0001	0.16
HHV-6B protein presence (%)	12/15 (80%)	18/49 (37%)	18/49 (37%)	16/50 (32%)	0.002	0.68	0.61
HHV-6B DNA presence (%)	8/15 (53%)	12/49 (24%)	10/50 (20%)	8/50 (16%)	0.006	0.33	0.68
HHV-6B protein and DNA presence (%)	7/15 (47%)	9/49 (18%)	3/48 (6%)	0/50 (0%)	<0.0001	0.001	0.11

*^∗^Pairwise comparisons performed by Fisher’s exact test, with two-tailed *p*-values. MDD, major depressive disorder; BPD, bipolar disease; SCZ, schizophrenia; CON, matched controls without neurological or psychiatric disorders.*

### Examination of Possible Confounding Clinical/Demographic Variables on HHV-6A and HHV-6B Protein or DNA Expression

No significant correlations for brain pH, PMI or age at death with HHV-6A or HHV-6B copy number were found for Cohort 1 or Cohort 2 (Spearman’s rho, *p* > 0.05). For the three disease groups (CON excluded from analysis), age of illness onset and duration of illness did not correlate with HHV-6A or HHV-6B DNA (Spearman’s rho, *p* > 0.05) nor HHV-6A protein or DNA frequency (one-way ANOVA, *p* > 0.05). However, for both HHV-6B DNA and protein a significant association with age of illness onset was found (one-way ANOVA, *p* = 0.005). Cases positive for HHV-6B DNA and protein were diagnosed with a psychiatric illness later than cases negative for HHV-6B DNA and protein.

For BPD and SCZ (CON and MDD excluded from the analysis), antipsychotic dose equivalents or clinical presentation of psychotic features did not associate with HHV-6A or HHV-6B protein or DNA frequency (one-way ANOVA and Fisher’s exact test respectively, *p* > 0.05).

No relationships for smoking at time of death, lifetime drug use, lifetime alcohol use, race or brain hemisphere were found with HHV-6A or HHV-6B protein or DNA frequency variables (Pearson’s Chi-square, *p* > 0.05). A higher frequency of HHV-6A protein and DNA positive cases and HHV-6B protein and DNA positive cases was found amongst suicide completers compared to other forms of death (Fisher’s exact, *p* = 0.02 and 0.047 respectively), however when controls were excluded from the analysis this no longer reached statistical significance.

A significant effect of gender was found for HHV-6A protein and HHV-6A protein and DNA (Fisher’s exact, *p* = 0.03 and 0.01 respectively). Specifically, a higher frequency of HHV-6A protein and HHV-6B protein and DNA was found amongst females compared to males. To explore potentially confounding effects of gender on HHV-6A or HHV-6B status, likelihood ratio tests in multinomial logistic regression revealed no significant effect of gender on HHV-6A or HHV-6B protein expression (Chi-square, *p* > 0.12). In summary, none of the clinical and demographic variables examined correlated significantly with HHV-6A or HHV-6B infection, except age of illness onset, which was higher in psychiatric patients with HHV-6B protein and DNA.

### Biological or Pathological Correlates of HHV-6A and HHV-6B

#### HHV-6A and HHV-6B and Purkinje Cell Cross-Sectional Soma Area

Cross-sectional soma area of RBFOX2-positive Purkinje cells was assessed for all cases to make comparisons across HHV-6A/B protein/DNA status groups (**Table 2**). As BPD diagnosis, has been previously associated with reduced cross-sectional soma area of Purkinje cells, diagnosis was included as a between-subjects factor in general linear model univariate analyses. A decrease in Purkinje cell cross-sectional area was found in cases positive for HHV-6A protein relative to negative cases (*p* = 0.002; **Table 2**). A decrease in Purkinje cell cross-sectional area was found in BPD relative to CON and SCZ (Bonferroni *post hoc* test, *p* < 0.05; **Table 2**). By contrast, an increase in Purkinje cell cross-sectional area was found in positive relative to negative cases for HHV-6B DNA (*p* = 0.02). In summary, Purkinje cell cross-sectional area was reduced in cerebellum samples positive for HHV-6A protein; by contrast, cross-sectional area was increased in HHV-6B DNA positive samples.

**Table 2:** Summary of estimated marginal mean soma area in μm^2^ (+SE) for Purkinje cells in cases with positive or negative HHV-6 status for HHV-6A and HHV-6B protein and DNA.

	Negative HHV-6 status Mean + (SE)	Positive HHV-6 status Mean + (SE)	Between-subjects effect of HHV-6 status	Between-subjects effect of diagnosis
HHV-6A protein (*N* = 162)	544 (20.3)	446 (18.6)	^∗^*F* = 12.6; *p* = 0.001	^∗∗^*F* = 5.19; *p* = 0.002
HHV-6A DNA (*N* = 160)	503 (30.0)	512 (15.5)	*F* = 0.08; *p* = 0.78	^∗∗^*F* = 7.0; *p* < 0.001
HHV-6A protein and DNA (*N*= 160)	529 (18.6)	485 (32.3)	*F* = 1.42; *p* = 0.24	^∗∗^*F* = 4.99; *p* = 0.002
HHV-6B protein (*N* = 161)	515 (23.4)	469 (18.2)	*F* = 2.42; *p* = 0.12	^∗∗^*F* = 5.22; *p* = 0.002
HHV-6B DNA (*N* = 160)	471 (16.6)	544 (23.5)	^∗^*F* = 6.63; *p* = 0.01	^∗∗^*F* = 3.35; *p* = 0.02
HHV-6B protein and DNA (*N* = 160)	484 (15.8)	490 (37.1)	*F* = 0.31; *p* = 0.58	^∗∗^*F* = 4.36; *p* = 0.006

*^∗^Significant difference in estimated marginal mean soma area according to HHV-6 status (General Linear Model, Univariate ANOVA using HHV-6 status and diagnosis as between-subjects factors; *p* < 0.05); ^∗∗^Mean soma area in BPD is significantly less than both CON and SCZ for HHV-6A protein; HHV-6 DNA; HHV-6A protein and DNA, HHV-6B protein, HHV-6B DNA, and HHV-6B protein and DNA; *p* < 0.05 using Bonferroni *post hoc* comparisons).*

#### Analysis of Published Microarray Data

We also performed functional profiling of a microarray dataset from cerebellum, where cases were classed as either HHV-6A (DNA and protein) positive or negative. Gene set enrichment analysis of Reactome pathways resulted in 254 significantly enriched pathways (*q*-value <= 0.05, **Supplementary Table S7**). Of the 254 pathways, 23 were associated with viral infection (highlighted with green within **Supplementary Table S7**), which is consistent with our findings of active virus in the cerebellum of HHV-6A positive cases. Pathways activated by HIV and influenza (from previous published studies of infected cells from non-CNS organs) were prominent. To our knowledge, there have been no previous studies indicating whether these pathways are enriched during infection with HHV-6.

The GSEA analysis also revealed significantly enriched pathways associated with immune response, in particular pathways involving interferons (4 pathways), MHC (5 pathways), and toll-like receptor signaling (11 of 254 pathways, highlighted with red within **Supplementary Table S7**); the latter is summarized in **Supplementary Figure S5**.

## Discussion

### Characteristics of HHV-6A and HHV-6B Expression in Human Cerebellum

In the present study, we examined expression of HHV-6A and HHV-6B protein and DNA in the posterior lobe of human cerebellum. Anatomical assessment of brain sections using DNA FISH and IFA showed that DNA for HHV-6A/B and four different HHV-6A/B proteins (detected by gB, OHV3, gp82/105, and U94 antibodies) localized to Purkinje cells at the interface of the granule cell and molecular layers. Occasionally, smaller cells surrounding Purkinje cells stained positive for HHV-6A/B; we identified HHV-6B in GFAP-positive astrocytes (**Figures 2A,B** and **Supplementary Figures S3, S4**). In summary, there appears to be a high tropism of HHV-6A and HHV-6B for GABAergic Purkinje cells, and very occasionally GABAergic interneurons of the molecular layer, and occasionally astrocytes or microglia in the vicinity of positively stained Purkinje cells. Previous studies in rodents have shown that Purkinje cells of the cerebellum are targeted during perinatal development by infection with Borna disease virus and influenza virus (Williams et al., 2007; Shi et al., 2009). For that reason, we speculate that primary infection with HHV-6A or HHV-6B within a specific developmental time window may lead to Purkinje cell tropism.

Previous studies in human neural cells have revealed that astrocytes, microglia, and oligodendrocytes are primary targets of HHV-6A/B infection (Albright et al., 1998). More recently, Esposito et al. (2015) revealed HHV-6A/B staining of glutamatergic pyramidal cells in human hippocampus (Donati et al., 2003). We extend these findings by highlighting a different neuronal population in the cerebellum targeted by HHV-6A and HHV-6B. Other than a study by Chan et al. (2001), where HHV-6A/B expression was detected in human cerebellum, cytoarchitectural expression of HHV-6A/B in human cerebellum has not been previously studied. Our observation that four different antibodies to HHV-6A/B proteins and DNA probes all target Purkinje cells, provides convincing evidence that HHV-6A and HHV-6B infect Purkinje cells of the human cerebellum in a significant proportion of cases. Occasionally, cases positive for HHV-6B in Purkinje cells also expressed positive HHV-6B staining of neighboring astrocytes or microglia. Further studies in human cerebellum and other brain regions using Z-axis imaging are required to extend our findings.

We opted to combine our data from our qPCR and protein studies for a number of reasons. Firstly, as granule cells (which were consistently HHV-6A/B negative) are the most prolific type of neuron in human brain and predominate the DNA pool from whole cerebellar cortex, it is possible that some samples with low viral copy numbers in qPCR assays fall below the LOD; and are therefore misinterpreted as HHV-6A/B negative. HHV-6 undergoes productive viral infection in CD4+ T cells. Other cell types, even though they allow viral infection, may be non-permissive to productive viral infection (Agut et al., 2015). It is plausible that HHV-6 infection in Purkinje cells is not equivalent to productive HHV-6 infection where one expects to see several 100 copies of viral genome. The viral genome can undergo reactivation without much viral DNA replication in cells non-permissive to productive viral infection. Under these conditions, viral DNA is not replicated but shows transcription and translation of several viral ORFs. Several studies have shown a decrease in HHV-6 DNA upon viral reactivation (Arbuckle et al., 2010; Prusty et al., 2013a). Hence, HHV-6 reactivation in specific cells that are potentially non-permissive to full viral productive infection should be considered. A recent large-scale study published during the time of evaluation of our manuscript (Readhead et al., 2018) showed a potential association of HHV-6 with Alzheimer’s disease. The entire viral genome could not be detected in most of the cases in this study. In addition, transcripts from specific viral regions were detected in the absence of full transcription of entire viral ORFs as expected in productive viral infection. These findings support our data that a fully productive viral infection is potentially not necessary to cause a disease. We speculate that this may lead to limitations in the use of qPCR alone. For these reasons, we applied a combination of both methods (IFA and qPCR) for data analysis.

### HHV-6A and HHV-6B Detection Is Increased in Mood Disorders in the Cerebellum

The 164 cases we examined belonged to one of three psychiatric groups or comparison controls. We detected HHV-6A late protein more frequently in the cerebellum from patients with BPD and MDD relative to comparison controls. When protein and DNA data was combined, material from donors with either BPD or MDD contained higher HHV-6A and HHV-6B DNA and protein relative to controls. In contrast, there was clearly no association of the proteins or DNA of either virus with SCZ.

This is the first study to report an association of HHV-6A and HHV-6B with mood disorders. The only previous study of HHV-6A/B in brain in mood disorders, by Conejero-Goldberg et al. (2003), identified HHV-6B DNA in one BPD and one CON case in the orbital fontal cortex, using a consensus PCR assay with low sensitivity (Conejero-Goldberg et al., 2003). Other studies using blood samples found an association of serum HHV-6A/B IgG with SCZ in military personnel (Niebuhr et al., 2008), and no difference from controls for HHV-6A and HHV-6B DNA in peripheral blood mononuclear cells (Yavarian et al., 2015). It is difficult to reconcile our findings with previous studies or to explain why we found HHV-6A and HHV-6B more frequently in MDD and BPD compared to SCZ. It could be speculated that a biological mechanism that is more frequently disrupted amongst MDD and BPD cases may facilitate tropism of HHV-6A and HHV-6B to Purkinje cells. Further studies in cerebellum and other brain regions are required to extend our findings that HHV-6A and HHV-6B are more frequently detected in MDD and BPD. Repetition using a larger collection is particularly important for MDD, given that the sample size for this group was much smaller than for the other diagnostic groups.

Other pathogens previously associated with BPD include influenza type-A, where prenatal exposure leads to a fourfold increase in the risk of developing BPD (Parboosing et al., 2013). Serological detection of HSV-1 antibodies in BPD has been associated with more severe cognitive impairments in BPD patients (Gerber et al., 2012). A study assessing CSF samples for antibodies for neurotropic agents found an increase in antibody production in BPD, but no specific pathogen was common amongst the cases; antibodies to HHV-6A/B were not measured (Stich et al., 2015).

A recent meta-analysis of infectious agents and MDD reported significant associations for Borna disease virus, HSV-1, varicella zoster virus, Epstein–Barr virus, and *Chlamydia trachomatis* (Wang et al., 2014). The association with *C. trachomatis* is relevant to our finding of increased HHV-6A and HHV-6B in MDD, since we have previously shown using HSB-2 cells and human primary leukocytes that *C. trachomatis* induces replication of latent HHV-6A/B (Prusty et al., 2013b). Other studies have also demonstrated co-operation between the Chlamydia family of bacteria and several HHVs; with co-infection implicated in chronic fatigue syndrome and multiple sclerosis (Munger et al., 2003; Nicolson et al., 2003). The meta-analysis (Wang et al., 2014) offers a preliminary insight into the various infectious agents that may play a role in the pathophysiology of MDD. The fact that other HHVs have been linked to MDD adds plausibility to our finding that two additional herpesviruses, HHV-6A and HHV-6B, may be linked to this disease. However major efforts are required to identify and dissect candidate pathogens further, their interactions within the host, temporal mechanisms and host-derived mechanisms resulting in individual differences in pathological outcome. Furthermore, repetition in a larger MDD cohort is required to validate our findings.

Previous studies have suggested transcription of multiple VZV mRNAs in human ganglia in post-mortem tissues because of restricted viral reactivation after death (Ouwendijk et al., 2012). It is highly unlikely that late viral proteins like gB, gp 82/105, and OHV3 could be translated in cells after the death of the patients. In addition, longer PMI did not correlate with an increase in variables for viral protein expression. Rather, we found that longer PMI associated with less HHV-6A.

### Pathological Correlates of HHV-6A and HHV-6B in Human Cerebellum

#### Possible Role of the Cerebellum in Mood Disorders

Traditionally, the cerebellum has been peripheral to the focus of pathological and *in vivo* imaging studies in MDD and BPD. However, recently there has been growing interest in the possible role of the cerebellum in these two mood disorders (DelBello et al., 1999; Maloku et al., 2010; Johnson et al., 2015). A meta-analysis of *in vivo* positron emission tomography studies in MDD highlighted abnormal metabolism in cerebellum amongst other brain regions (Su et al., 2014). In BPD, reduced numbers of Purkinje cells, cerebellar atrophy and microstructural/metabolic abnormalities have been reported (DelBello et al., 1999; Maloku et al., 2010; Johnson et al., 2015). Maldevelopment of the cerebellum may lead to structural and functional abnormalities of fronto-limbic brain structures that in MDD and BPD are more commonly associated with pathophysiology (Reeber et al., 2013). Indeed, cerebellar tumor resection during early childhood leads to cognitive dysfunction and associated structural deficits of the cortex and hippocampus (Moberget et al., 2015). Consequently, it is plausible that increased HHV-6A and HHV-6B infection in BPD and MDD presented here, could impact structure and function of fronto-limbic brain regions; highlighting the need for future studies of HHV-6A and HHV-6B in other brain regions.

To assess the potential impact of HHV-6A and HHV-6B infection in the cerebellum, we carried out morphological and cell density studies of Purkinje cells and also re-analyzed published microarray data (Chen et al., 2013) to enquire if functional changes occur in HHV-6A positive cases.

#### Purkinje Cell Size

Morphometric analysis of Purkinje cells across all diagnostic groups, revealed that the size of the Purkinje cell soma was reduced in cases positive for HHV-6A protein. As expected we found a robust difference in Purkinje cell size between diagnostic groups; specifically, a decrease in Purkinje cell size has been previously reported in psychiatric illness (Tran et al., 1998). Despite the significant effect of diagnosis, when included as a between-subjects factor using Univariate analysis, we still found that HHV-6A protein expression associates with reduced size of Purkinje cells. Further studies of HHV-6A infection of Purkinje cells are required to confirm these findings and to shed light on possible pathological mechanisms.

#### Microarray Data Analysis

Gene expression in the cerebellum of subjects that were positive for both HHV-6A DNA and protein was compared to gene expression in those who were negative for both HHV-6A DNA and protein. The GSEA analysis revealed 254 enriched pathways and most notably changes in pathways involving response to viral infection (particularly to HIV and influenza virus) were overrepresented. HIV and influenza virus have been associated previously with MDD and BPD (Del Guerra et al., 2013; Parboosing et al., 2013; Bornand et al., 2016). Unfortunately, Reactome thus far only includes a small number of pathways that have been explicitly linked to HSV-1 in humans; to our knowledge, there has not yet been an attempt to explicitly link gene expression in cerebellar tissue to infection with the other human herpesviridae family, including HHV-6A. It is conceivable that there are shared genes and pathways activated during HHV-6A, HIV and influenza infection (Bornand et al., 2016) that have not yet been identified. In any event, the present findings are consistent with the inference that HHV-6A can infect Purkinje cells. Gene expression studies are required to validate these findings and explore the possibility of shared pathogenic mechanisms amongst different classes of viruses implicated in mood disorder, including HHV-6A. As we did not assess infection with other viruses, these findings should be interpreted with caution.

The GSEA analysis also revealed significantly enriched pathways involved in the innate immune response, including toll-like receptor signaling (11 of 254 pathways). This class of pattern recognition receptors plays an important role in innate immunity and activation by viruses and other pathogens triggers molecular cascades leading to the production of cytokines and chemokines (Kawai and Akira, 2011). Expression studies of TLRs in human brain are limited, but evidence suggests that TLRs are expressed by microglia, macroglia, and neurons (Atmaca et al., 2014). Dysfunction of TLRs has been implicated in BPD and MDD (Garcia Bueno et al., 2016). Furthermore, disruption of TLR4 signaling is induced by HHV-6B (Murakami et al., 2010) and TLR9 activation plays a key role in inducing pro-inflammatory signaling in a mouse model of HHV-6A infection (Reynaud et al., 2014).

Overall, the GSEA findings suggest that HHV-6A infection of human cerebellum may activate pathways involved in the response to infection. We make this inference cautiously, because the proportion of mRNA from infected Purkinje cells was low relative to mRNA from granule cells and other cell types. Laser micro-dissection of Purkinje cells should be used in future to probe differentially expressed genes further. Nevertheless, given that these pathways have been implicated previously in HHV-6A and mood disorders, this provides a compelling area for further research.

## Conclusion

To our knowledge, this is the first study to report that HHV-6A and HHV-6B may infect cerebellar Purkinje cells, and that such infection may be associated with both BPD and MDD. The gene expression studies suggest that the immune response to such infection may contribute to the pathophysiology of these mood disorders. A limitation of this study is that only a small region of the posterior lobe was examined. To better understand the possible pathological effects of HHV-6A and HHV-6B in cerebellum, stereological and laser-capture microdissection studies of HHV-6A/B positive Purkinje cells should be carried out. That analysis should distinguish between sub-anatomic regions of the cerebellar cortex (identified by the presence/absence of ALDOC also known as Zebrin II) because Purkinje cells in these two regions in rodents are known to differ in sensitivity to genetic, chemical and physical insults and perinatal infection with at least one viral infection, Borna disease virus (Williams et al., 2007; Cerminara et al., 2015). Furthermore, role of co-infection of other pathogens together with HHV-6A and HHV-6B should be studied in mood disorder patients in detail as a number of severe infections have been associated with increased risk of MDD, bipolar and SCZ in a dose-dependent manner (Benros et al., 2013; Prusty et al., 2013b) and infections with other pathogens have the potential to reactivate latent HHV-6A/B (Benros et al., 2013; Prusty et al., 2013b).

## Author Contributions

BP and CT designed the project and wrote the paper. BP and NG designed and carried out qPCR, IFA, FISH, and TEM studies. CT did DNA extractions and performed all statistical analysis. LL and JF carried out microarray data analysis. JF advised on statistical analyses. DA and GK contributed to design and interpretation of IHC and TEM experiments. RA conceived and SG carried out preliminary qPCR studies (data not included). All authors read and approved the manuscript.

## Conflict of Interest Statement

The authors declare that the research was conducted in the absence of any commercial or financial relationships that could be construed as a potential conflict of interest.

## Footnotes

1 http://www.stanleyresearch.org/brain-research/array-collection/

## Supplementary Material

FIGURE S1 HHV-6A and HHV-6B detection through the use of immunofluorescence staining was validated using FFPE sections of HHV-6A and HHV-6B-infected HSB-2 and Molt-3 cells respectively. Uninfected cells served as control.

FIGURE S2 Representative confocal microscopy images showing Purkinje cell-specific staining for HHV-6A **(left)** and HHV-6B **(right)**. Cryo-sectioned cerebellar cortex samples were stained using antibodies raised against HHV-6A gp82/105 or HHV-6B OHV3 (Red). HHV-6 positive Purkinje cells are marked with white arrowhead. Purkinje cell specificity was tested using monoclonal antibodies against human Fox2 (Green). DAPI (Blue) was used to stain DNA. The scale bars (indicated with white lines) represent 200 μm.

FIGURE S3 Immuno-fluorescence analysis to study HHV-6 infection in astrocytes and microglia within cerebellar cortex samples. **(A)** Representative images showing positive staining for astrocytes in HHV-6A and HHV-6B negative samples. **(B)** Representative images showing HHV-6A and -6B positivity only in Purkinje cells. Astrocytes were detected as negative for HHV-6 or it was difficult to conclude HHV-6 positivity in astrocytes. **(C)** Representative images showing HHV-6B positivity in both Purkinje cells as well as astrocytes. HHV-6 positive astrocytes are marked with white arrowheads. **(D)** Representative images showing HHV-6A and -6B positivity in microglial cells. HHV-6 positive astrocytes and microglial cells are marked with white arrowheads. Cryo-sectioned cerebral cortex samples were stained using monoclonal antibodies against gp82/105 and OHV3 together with GFAP or Iba1 antibodies (marker for astrocytes and microglia respectively). The scale bars (indicated with white lines) represent 200 μm.

FIGURE S4 Representative images from confocal image analysis to study HHV-6 infection in astrocytes within cerebellar cortex samples. Red arrowheads point to HHV-6 positive cells whereas white arrowheads point to astrocytes showing HHV-6 positive co-staining. The scale bars represent 200 μm.

FIGURE S5 Enriched pathway network for the toll-like receptors component of the GSEA results. A connection between nodes arises when there is sufficient similarity between the gene sets of these pathways (based on the Dice coefficient). The node colour represents the *q*-value (more intensive shades of red correspond to more significant *q*-values), whereas the node size accounts for the gene set size of the pathway.

TABLE S1 Table showing median age of all the four types of cases from both cohorts 1 and 2. BPD, bipolar disorder; MDD, major depressive disorder; SCZ, schizophrenia; CON, controls.

TABLE S2 Table showing the cause of death for both the cohorts 1 and 2. BPD, bipolar disorder; MDD, major depressive disorder; SCZ, schizophrenia; CON, controls.

TABLE S3 Antibodies used for Immunohistochemistry and Western Blotting.

TABLE S4 Mean (+SD) virus load (per 10e6 cells) for total HHV-6, HHV-6A, and HHV-6B DNA for cohorts 1 and 2. Viral copy numbers were set as 0 for all samples with viral DNA copies below the limit of detection (LOD).

TABLE S5 Real-time based qPCR analysis showing quantification of HHV-6 genome equivalents per cell all the samples from both the cohorts 1 and 2. Mean Cq values as obtained from qPCR analysis and the subsequent calculations are described in detail.

TABLE S6 Summary of results from various types of tests carried out on both the cohort of samples.

TABLE S7 Summary of gene set enrichment analysis (GSEA) analysis showing significantly enriched pathways.
