# Supplementary figures and images for: Active HHV-6 Infection of Cerebellar Purkinje Cells in Mood Disorders
Source: Front Microbiol. 2018 Aug 21;9:1955. doi: 10.3389/fmicb.2018.01955 (PMC6110891; doi:10.3389/fmicb.2018.01955)

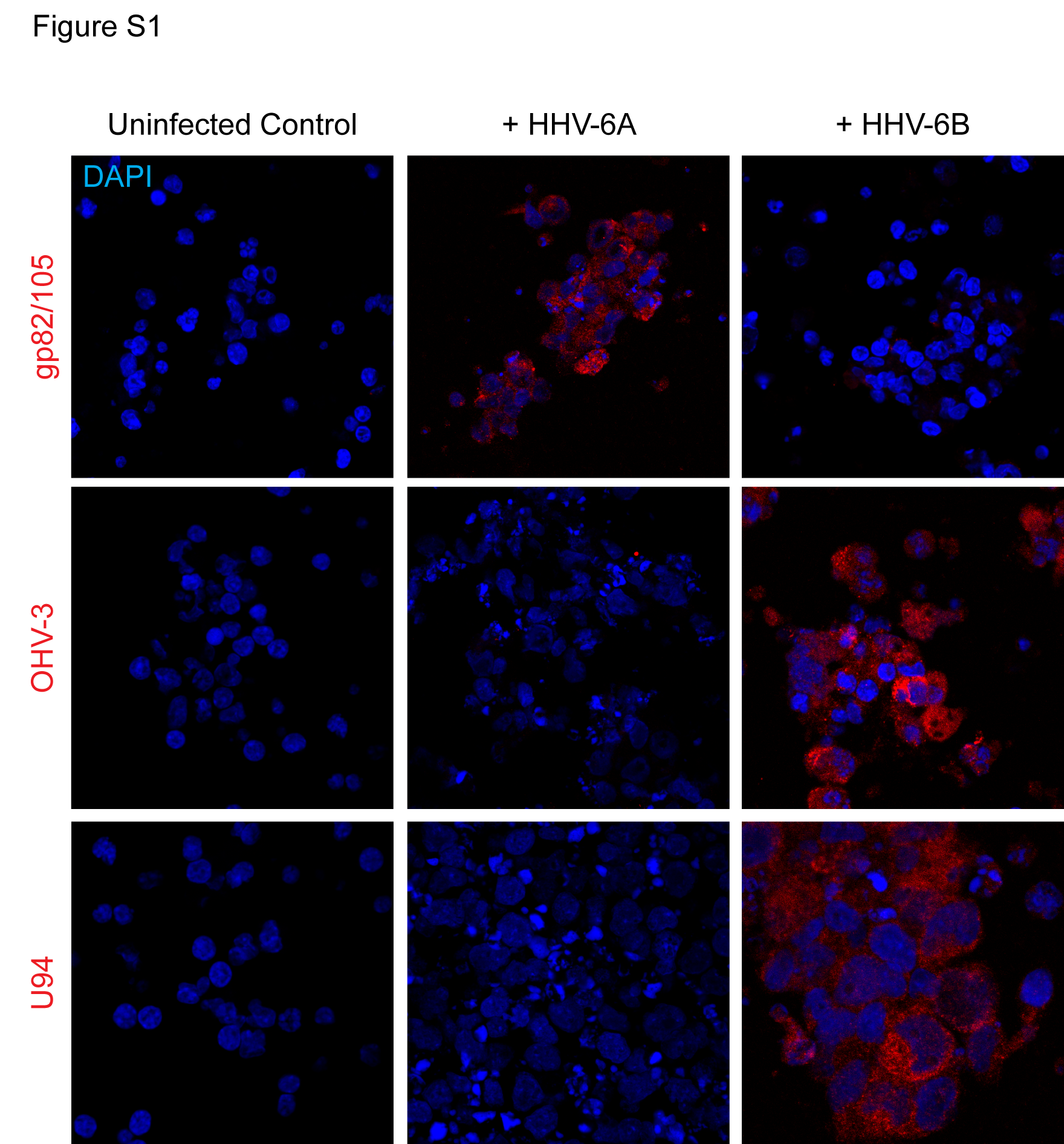

Supplement: FIGURE S1 — HHV-6A and HHV-6B detection through the use of immunofluorescence staining was validated using FFPE sections of HHV-6A and HHV-6B-infected HSB-2 and Molt-3 cells respectively. Uninfected cells served as control. [file Image_1.TIF]

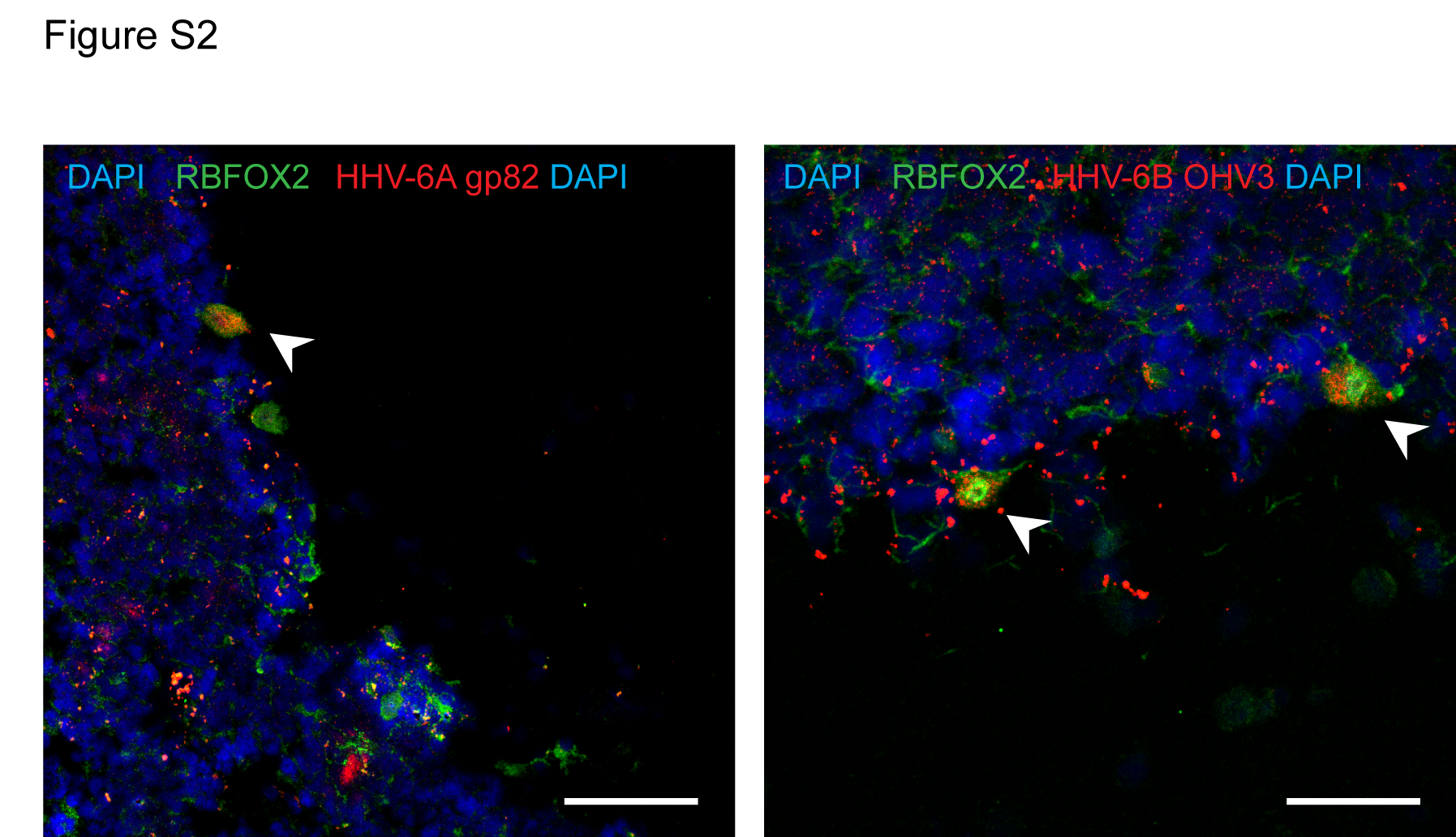

Supplement: FIGURE S2 — Representative confocal microscopy images showing Purkinje cell-specific staining for HHV-6A (left) and HHV-6B (right). Cryo-sectioned cerebellar cortex samples were stained using antibodies raised against HHV-6A gp82/105 or HHV-6B OHV3 (Red). HHV-6 positive Purkinje cells are marked with white arrowhead. Purkinje cell specificity was tested using monoclonal antibodies against human Fox2 (Green). DAPI (Blue) was used to stain DNA. The scale bars (indicated with white lines) represent 200 μm. [file Image_2.TIF]

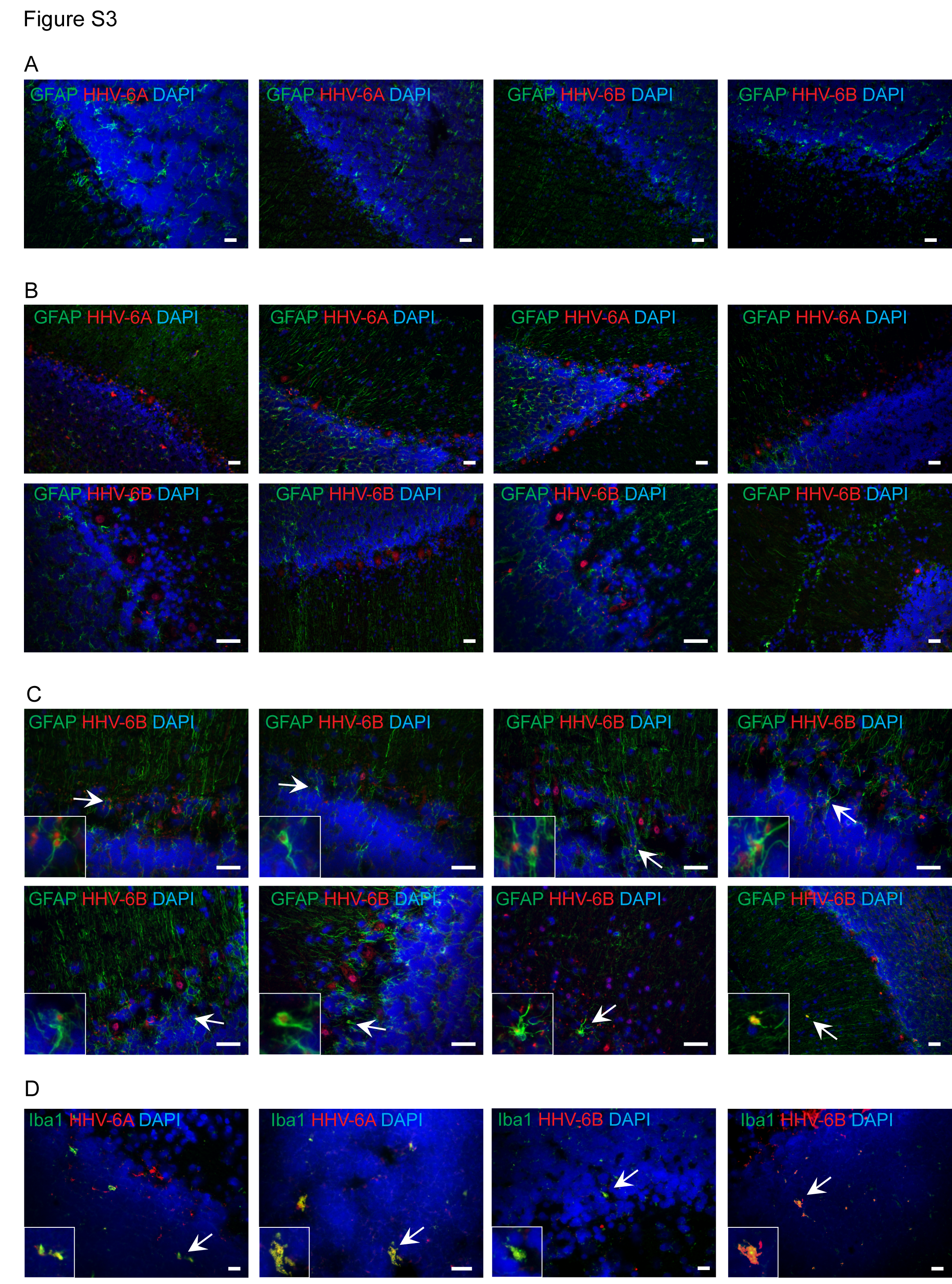

Supplement: FIGURE S3 — Immuno-fluorescence analysis to study HHV-6 infection in astrocytes and microglia within cerebellar cortex samples. (A) Representative images showing positive staining for astrocytes in HHV-6A and HHV-6B negative samples. (B) Representative images showing HHV-6A and -6B positivity only in Purkinje cells. Astrocytes were detected as negative for HHV-6 or it was difficult to conclude HHV-6 positivity in astrocytes. (C) Representative images showing HHV-6B positivity in both Purkinje cells as well as astrocytes. HHV-6 positive astrocytes are marked with white arrowheads. (D) Representative images showing HHV-6A and -6B positivity in microglial cells. HHV-6 positive astrocytes and microglial cells are marked with white arrowheads. Cryo-sectioned cerebral cortex samples were stained using monoclonal antibodies against gp82/105 and OHV3 together with GFAP or Iba1 antibodies (marker for astrocytes and microglia respectively). The scale bars (indicated with white lines) represent 200 μm. [file Image_3.TIF]

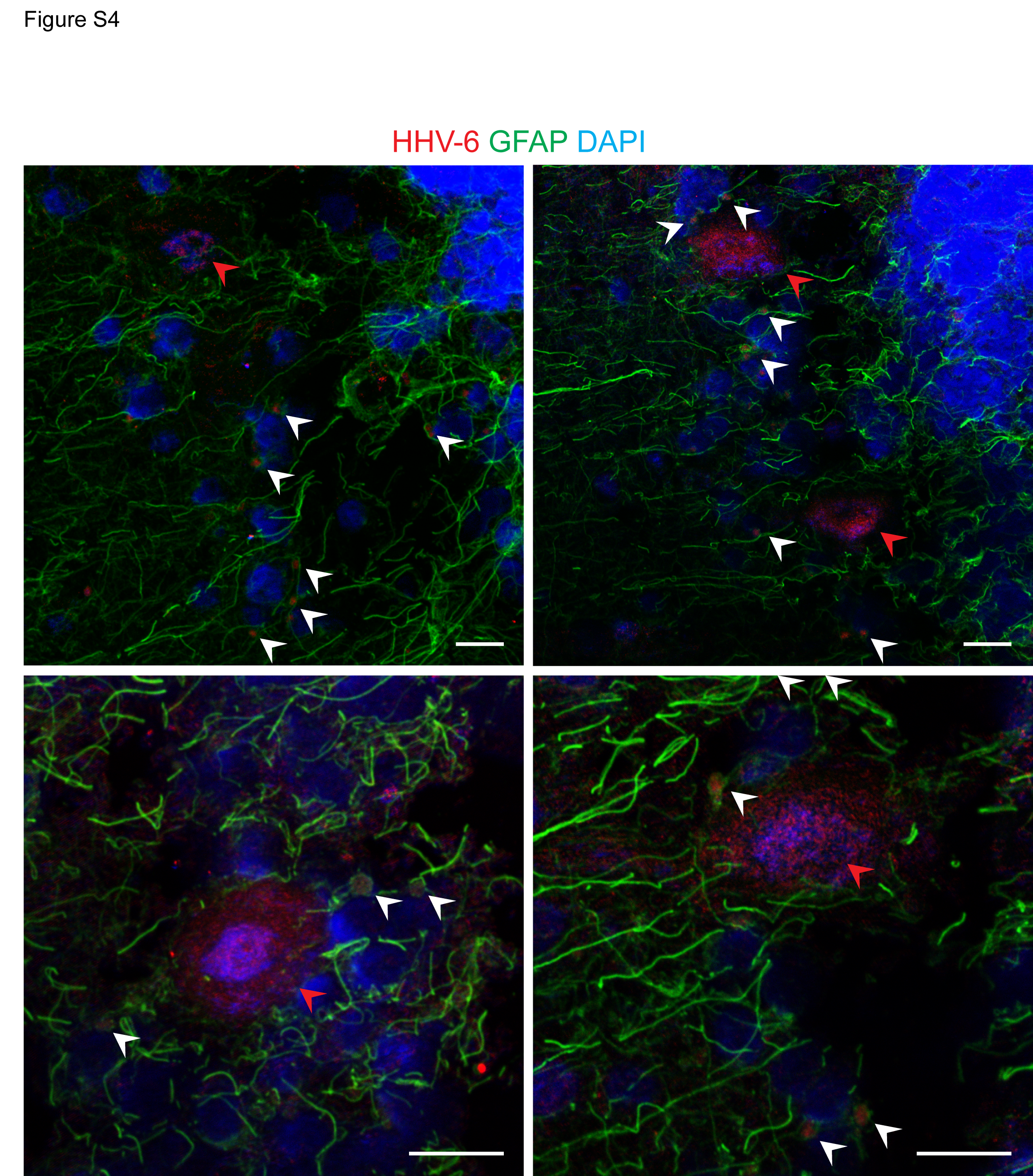

Supplement: FIGURE S4 — Representative images from confocal image analysis to study HHV-6 infection in astrocytes within cerebellar cortex samples. Red arrowheads point to HHV-6 positive cells whereas white arrowheads point to astrocytes showing HHV-6 positive co-staining. The scale bars represent 200 μm. [file Image_4.TIF]

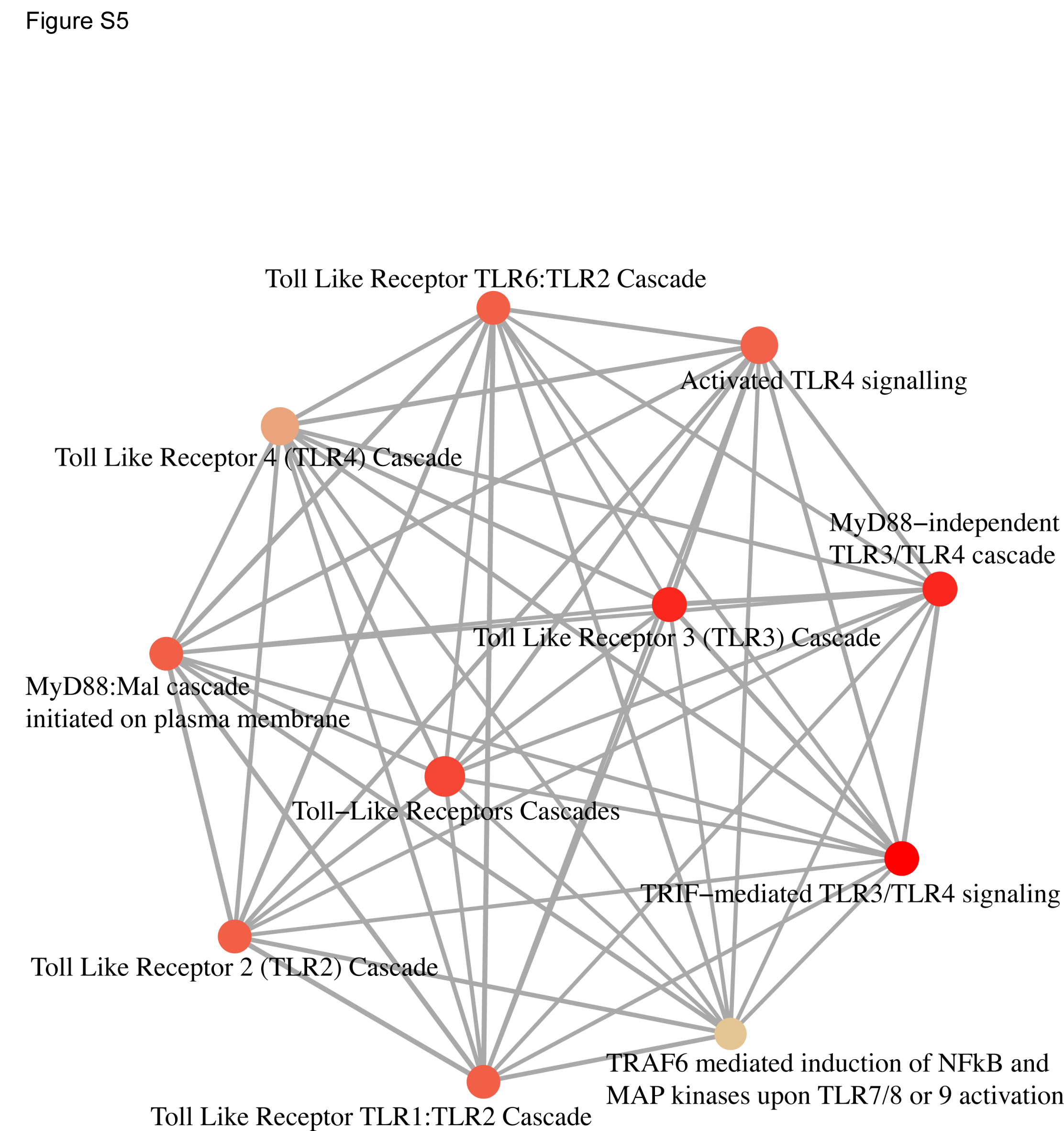

Supplement: FIGURE S5 — Enriched pathway network for the toll-like receptors component of the GSEA results. A connection between nodes arises when there is sufficient similarity between the gene sets of these pathways (based on the Dice coefficient). The node colour represents the q-value (more intensive shades of red correspond to more significant q-values), whereas the node size accounts for the gene set size of the pathway. [file Image_5.TIF]
