# Supplementary material for: Active HHV-6 Infection of Cerebellar Purkinje Cells in Mood Disorders
Source: Front Microbiol. 2018 Aug 21;9:1955. doi: 10.3389/fmicb.2018.01955 (PMC6110891; doi:10.3389/fmicb.2018.01955)
Supplement: TABLE S1 — Table showing median age of all the four types of cases from both cohorts 1 and 2. BPD, bipolar disorder; MDD, major depressive disorder; SCZ, schizophrenia; CON, controls. [file Data_Sheet_1.PDF]

Table S1. Table showing median age of all the four types of cases from both cohorts 1 and 2. BPD, bipolar disorder; MDD, major depressive disorder; SCZ, schizophrenia; CON, controls.

Diagnosis	Count	Median age (in years) at death
BPD	49	45
MDD	15	46
SCZ	50	44
CON	50	46
