# Supplementary material for: Active HHV-6 Infection of Cerebellar Purkinje Cells in Mood Disorders
Source: Front Microbiol. 2018 Aug 21;9:1955. doi: 10.3389/fmicb.2018.01955 (PMC6110891; doi:10.3389/fmicb.2018.01955)
Supplement: TABLE S2 — Table showing the cause of death for both the cohorts 1 and 2. BPD, bipolar disorder; MDD, major depressive disorder; SCZ, schizophrenia; CON, controls. [file Data_Sheet_2.PDF]

Table S2. Table showing the cause of death for both the cohorts 1 and 2. BPD, bipolar disorder; MDD, major depressive disorder; SCZ, schizophrenia; CON, controls.

	BPD	MDD	SCZ	CON
Acute Pancreatic disease	0	0	1	0
Asthma	0	0	0	1
Cancer	0	0	0	1
Cardiac diseases	8	6	20	41
Cirrhosis	0	0	1	0
Chronic Obstructive Pulmonary Disorder	0	0	1	0
Drowning	3	0	1	0
Exhaustive mania/NMS	0	0	1	0
Fall	1	0	0	0
Ketoacidosis	1	0	0	0
Motor Vehicle Accident	0	0	2	2
Myocarditis	1	0	0	1
Drug overdose	6	1	4	0
Pneumonia	3	0	6	0
Pulmonary Embolism	1	1	1	4
Sleep Apnea	1	0	0	0
Suicide	24	7	11	0
Unknown	0	0	1	0
**Total**	**50**	**15**	**50**	**50**
