# Supplementary material for: Active HHV-6 Infection of Cerebellar Purkinje Cells in Mood Disorders
Source: Front Microbiol. 2018 Aug 21;9:1955. doi: 10.3389/fmicb.2018.01955 (PMC6110891; doi:10.3389/fmicb.2018.01955)
Supplement: TABLE S3 — Antibodies used for Immunohistochemistry and Western Blotting. [file Data_Sheet_3.PDF]

Table S3. Antibodies used for Immunohistochemistry and Western Blotting.

Antibody	Dilution	Source
Monoclonal mouse anti- HHV-6A gp82/105 (U100)	1:100	NIH AIDS Reagent Program (Cat. No. 12178)
Monoclonal mouse anti- HHV-6B U94	1:100	NIH AIDS Reagent Program (Cat. No. 12175)
Monoclonal mouse anti- HHV-6B p98-OHV3 clone	1:100	NIH AIDS Reagent Program (Cat. No. 12188)
Monoclonal rabbit anti- HHV-6 gB	1:100	generously provided by Yasuko Mori, Kobe University, Japan
Monoclonal mouse anti- NueN conjugated with Alexa Fluor488	1:100	MAB 377X, Millipore
Monoclonal rabbit anti- Fox2 antibody	1:100	ABE 184, Millipore
Monoclonal rabbit anti-Ibal antibody	1:100	Ab178846, Abcam
Monoclonal rabbit anti-GFAP antibody	1:100	AB5804, Millipore

DAPI was used for counter staining the nuclei. Respective mouse or rabbit secondary antibodies tagged to Cy3 or Cy5 were used for Immunohistochemistry.
