# Supplementary material for: Active HHV-6 Infection of Cerebellar Purkinje Cells in Mood Disorders
Source: Front Microbiol. 2018 Aug 21;9:1955. doi: 10.3389/fmicb.2018.01955 (PMC6110891; doi:10.3389/fmicb.2018.01955)
Supplement: TABLE S4 — Mean (+SD) virus load (per 10e6 cells) for total HHV-6, HHV-6A, and HHV-6B DNA for cohorts 1 and 2. Viral copy numbers were set as 0 for all samples with viral DNA copies below the limit of detection (LOD). [file Data_Sheet_4.PDF]

Table S4. Mean (+SD) virus load (per 10^6 cells) for total HHV-6, HHV-6A and HHV-6B DNA for cohorts 1 and 2. Viral copy numbers were set as 0 for all samples with viral DNA copies below the limit of detection (LOD).

Cohort 1 (N)	BPD (15)	MDD (15)	CON (15)	SCZ (14)	TOTAL (59)
**HHV-6A DNA** [a]	3.795 (3.903)	2.512 (3.433)	1.039 (1.910)	604 (1.168)	1.940 (3.051)
**HHV-6B DNA** [a]	3.535 (4.263)	2.068 (3.714)	878 (1.399)	604 (1.168)	1.791 (3.140)
**Cohort 2 (N)**	**BPD (34)**	**MDD (0)**	**CON (35)**	**SCZ (33)**	**TOTAL (102)**
**HHV-6A DNA** [b]	721 (3.755)	-	206 (391)	1.443 (5.903)	778 (3.995)
**HHV-6B DNA** [b]*	6.862 (40.011)	-	14.7 (49.8)	18.133 (103.813)	8.159 (63.195)

[a] Significant difference in viral copy number for HHV-6. HHV-6A and HHV-6B between diagnostic groups (Kruskal-Wallis, $p < 0.02$; NB this statistical test does not use mean values in the model).

[b] no significant difference across the diagnostic groups for HHV-6. HHV-6A and HHV-6B (Kruskal-Wallis, $p > 0.05$).

*HHV-6B viral load for two cases from cohort 2 (sample 33 and 105 in supplementary table S5) are included in the mean and SD summaries here but excluded from the statistical analysis as they had significantly high viral load mimicking possible chromosomally integrated and inherited HHV-6B (iciHHV-6) conditions and hence considered outliers.
