# Supplementary material for: Active HHV-6 Infection of Cerebellar Purkinje Cells in Mood Disorders
Source: Front Microbiol. 2018 Aug 21;9:1955. doi: 10.3389/fmicb.2018.01955 (PMC6110891; doi:10.3389/fmicb.2018.01955)
Supplement: TABLE S5 — Real-time based qPCR analysis showing quantification of HHV-6 genome equivalents per cell all the samples from both the cohorts 1 and 2. Mean Cq values as obtained from qPCR analysis and the subsequent calculations are described in detail. [file Data_Sheet_5.PDF]

Table S5. Real-time based qPCR analysis showing quantification of HHV-6 genome equivalents per cell all the samples from both the cohorts 1 and 2. Mean Cq values as obtained from qPCR analysis and the subsequent calculations are described in detail.

Sample number	HHV-6 U94 Cq	log10 copies	Actual viral copies	HHV-6A DR Cq	Log10 copies	Actual copies	HHV-6B DR Cq	Log10 copies	Actual copies	PI15 Cq	Log10 copies	Actual copies	Cell number	Viral copies / 10^6 cells	HHV-6A / 10^6 cells	HHV-6B /10^6 cells
COHORT 1																
1	26,51	2,30	201,83	30,24	0,61	41,08	26,45	0,77	58,66	18,71	3,07	59172,28	29586,14	6821,83	1388,65	1982,83
2	30,84	0,97	9,42	33,04	-0,20	6,37	27,25	0,52	32,75	16,57	3,83	338192,54	169096,27	55,69	37,66	193,65
3	29,43	1,41	25,55	32,27	0,03	10,61	26,19	0,85	70,76	18,99	2,97	47026,74	23513,37	1086,54	451,25	3009,45
4	28,37	1,73	54,29	31,72	0,19	15,33	26,89	0,63	42,56	17,32	3,57	184543,74	92271,87	588,42	166,17	461,27
5	30,48	1,09	12,19	31,37	0,29	19,33	25,63	1,03	106,52	16,49	3,86	362459,02	181229,51	67,28	106,66	587,77
6	22,65	3,49	3106,48	27,61	1,37	235,74	24,63	1,35	221,45	17,79	3,40	125393,80	62696,90	49547,57	3760,06	3532,12
7	22,67	3,49	3060,64	27,40	1,43	272,02	24,74	1,31	204,66	17,69	3,43	135933,89	67966,94	45031,34	4002,25	3011,10
8	27,74	1,93	84,99	30,98	0,40	25,08	29,70	-0,26	5,45	16,66	3,80	315036,61	157518,30	539,54	159,20	34,62
9	27,35	2,05	111,83	31,06	0,37	23,68	26,86	0,64	43,55	18,76	3,06	56790,25	28395,13	3938,48	834,11	1533,70
10	25,13	2,73	538,78	29,24	0,90	79,65	27,09	0,57	36,75	21,41	2,12	6576,64	3288,32	163845,59	24221,75	11176,13
11	23,74	3,16	1442,21	28,29	1,18	149,81	27,10	0,56	36,46	19,37	2,84	34583,20	17291,60	83405,01	8663,97	2108,34
12	33,08	0,29	1,93	33,19	-0,24	5,77	28,54	0,11	12,76	17,10	3,64	219719,00	109859,50	17,61	52,56	116,17
13	25,94	2,48	303,28	30,78	0,46	28,63	28,09	0,25	17,75	19,81	2,69	24247,85	12123,92	25015,19	2361,68	1464,16
14	26,57	2,29	194,65	31,28	0,31	20,55	28,42	0,14	13,92	19,33	2,85	35782,02	17891,01	10879,65	1148,66	778,09
15	24,88	2,81	640,81	29,12	0,94	86,62	26,96	0,61	40,38	20,38	2,48	15241,84	7620,92	84085,07	11365,83	5298,81
16	25,42	2,64	438,79	30,51	0,53	34,24	26,71	0,69	48,61	18,95	2,99	48903,47	24451,73	17945,19	1400,45	1988,14
17	28,41	1,72	52,82	31,62	0,22	16,41	30,51	-0,52	3,02	17,16	3,62	209059,59	104529,80	505,28	156,99	28,85
18	25,92	2,49	306,71	30,66	0,49	30,94	27,89	0,31	20,45	19,82	2,68	23978,53	11989,27	25582,46	2580,62	1705,37
19	26,67	2,26	180,71	29,64	0,79	61,31	25,60	1,04	108,90	20,37	2,48	15254,27	7627,14	23692,41	8038,33	14277,65
20	26,93	2,18	150,57	31,37	0,29	19,29	30,72	-0,58	2,60	17,74	3,42	130717,27	65358,64	2303,81	295,16	39,79
21	24,29	2,99	972,98	28,42	1,14	138,04	29,66	-0,25	5,64	18,23	3,24	87525,92	43762,96	22232,91	3154,16	128,79
22	25,53	2,61	406,50	29,97	0,69	49,06	29,97	-0,35	4,49	17,73	3,42	131894,78	65947,39	6163,95	743,88	68,15
23	25,19	2,71	516,74	30,17	0,63	42,86	30,32	-0,46	3,48	18,28	3,23	84441,93	42220,97	12238,92	1015,04	82,43
24	26,56	2,29	194,80	30,97	0,40	25,29	27,94	0,29	19,67	19,68	2,73	26903,92	13451,96	14481,12	1880,00	1462,22
25	27,88	1,88	76,57	31,69	0,19	15,59	30,83	-0,62	2,39	16,50	3,86	359486,79	179743,39	426,01	86,75	13,32
26	28,52	1,69	48,91	32,05	0,09	12,30	30,91	-0,65	2,26	17,42	3,53	170068,05	85034,03	575,16	144,70	26,58
27	35,89	-0,58	0,26	35,87	-1,02	0,97	33,44	-1,45	0,36	15,30	4,28	956051,14	478025,57	0,55	2,02	0,74
28	26,54	2,30	197,48	31,49	0,25	17,80	28,52	0,11	12,89	19,49	2,80	31424,14	15712,07	12568,62	1132,85	820,29
29	31,13	0,88	7,67	32,34	0,01	10,14	30,82	-0,62	2,41	17,39	3,54	173456,64	86728,32	88,47	116,89	27,82
30	29,86	1,28	18,92	32,40	-0,01	9,77	30,97	-0,67	2,15	15,97	4,04	552827,82	276413,91	68,46	35,34	7,79
31	26,08	2,44	274,86	30,32	0,59	38,90	27,59	0,41	25,48	20,45	2,46	14328,47	7164,24	38365,95	5429,32	3556,44
32	31,76	0,69	4,91	33,12	-0,22	6,02	30,82	-0,62	2,40	15,55	4,19	781042,85	390521,43	12,59	15,40	6,15
33	28,11	1,81	65,02	32,87	-0,15	7,11	31,49	-0,83	1,48	16,06	4,01	512922,80	256461,40	253,52	27,74	5,75
34	33,22	0,24	1,76	34,95	-0,75	1,79	32,23	-1,06	0,86	14,99	4,39	1227538,58	613769,29	2,86	2,91	1,40
35	26,28	2,38	237,90	29,73	0,76	57,52	25,65	1,02	105,43	19,41	2,83	33484,55	16742,28	14209,80	3435,70	6297,26
36	27,32	2,06	113,81	31,55	0,24	17,18	31,93	-0,97	1,07	15,80	4,10	635733,86	317866,93	358,06	54,05	3,36
37	27,34	2,05	112,68	32,02	0,10	12,56	31,91	-0,96	1,09	15,37	4,26	899932,75	449966,37	250,41	27,92	2,42
38	31,58	0,75	5,60	33,36	-0,29	5,13	31,49	-0,83	1,48	15,25	4,30	990968,05	495484,02	11,29	10,36	2,99
39	25,62	2,58	380,87	30,38	0,57	37,42	27,60	0,40	25,27	20,79	2,34	10899,68	5449,84	69886,05	6866,94	4637,13
40	26,54	2,30	197,49	31,85	0,15	14,06	31,67	-0,89	1,30	16,72	3,78	300805,63	150402,82	1313,09	93,49	8,63
41	25,07	2,75	562,15	31,72	0,19	15,32	28,21	0,21	16,25	16,05	4,02	519276,03	259638,01	2165,14	59,01	62,60
42	27,92	1,87	74,50	33,34	-0,28	5,21	28,80	0,02	10,50	17,97	3,34	108456,06	54228,03	1373,89	96,04	193,66
43	27,41	2,03	107,24	30,88	0,43	26,75	27,28	0,50	31,87	22,02	1,90	3984,42	1992,21	53827,87	13427,95	15998,34

44	28,54	1,68	47,97	32,37	0,00	9,91	27,32	0,49	30,95	16,85	3,73	270490,66	135245,33	354,68	73,30	228,88
45	30,70	1,02	10,42	34,49	-0,61	2,43	28,88	0,00	9,96	18,62	3,11	63843,46	31921,73	326,42	76,08	311,90
46	26,55	2,29	197,19	31,55	0,23	17,17	25,20	1,17	146,57	17,05	3,66	229233,77	114616,89	1720,39	149,80	1278,82
47	25,86	2,51	321,13	31,58	0,23	16,83	27,67	0,38	23,99	17,12	3,64	217225,50	108612,75	2956,69	154,96	220,87
48	25,02	2,76	579,94	29,08	0,95	88,60	25,10	1,20	157,34	18,91	3,00	50236,98	25118,49	23088,03	3527,32	6263,75
49	27,70	1,94	87,12	27,43	1,42	265,76	25,36	1,11	130,22	17,97	3,34	108367,68	54183,84	1607,85	4904,70	2403,26
50	27,14	2,11	129,25	30,55	0,52	33,44	29,85	-0,31	4,89	19,00	2,97	46892,75	23446,37	5512,69	1426,39	208,39
51	28,10	1,82	65,87	32,42	-0,02	9,62	28,24	0,20	15,87	17,10	3,64	219719,00	109859,50	599,58	87,58	144,42
52	26,68	2,25	179,85	31,69	0,20	15,67	25,90	0,94	87,39	16,81	3,75	279001,45	139500,72	1289,24	112,35	626,47
53	25,92	2,49	308,26	29,85	0,73	53,12	25,89	0,94	87,93	18,95	2,99	48692,62	24346,31	12661,48	2181,67	3611,66
54	25,86	2,51	320,95	29,66	0,78	60,30	25,67	1,02	103,60	19,60	2,76	28600,25	14300,12	22444,02	4216,57	7244,58
55	26,67	2,26	180,87	26,76	1,62	414,86	24,56	1,37	232,90	18,24	3,24	86603,20	43301,60	4177,02	9580,72	5378,55
56	26,88	2,19	156,33	31,87	0,14	13,82	27,59	0,41	25,43	17,35	3,55	179352,44	89676,22	1743,27	154,11	283,61
57	29,06	1,52	33,34	31,41	0,28	18,87	28,00	0,28	18,85	16,35	3,91	407276,30	203638,15	163,72	92,67	92,57
58	26,96	2,17	147,62	30,02	0,68	47,39	27,24	0,52	33,03	19,25	2,88	38013,33	19006,66	7766,68	2493,23	1737,99
59	29,87	1,27	18,75	30,21	0,62	41,98	30,32	-0,46	3,47	16,40	3,89	388150,14	194075,07	96,62	216,31	17,86
60	35,75	-0,53	0,29	32,90	-0,16	6,98	31,20	-0,74	1,82	18,47	3,16	72206,97	36103,49	8,09	193,34	50,40
COHORT 2																
1	33,82	0,06	1,15	35,59	-0,93	1,16	33,80	-1,56	0,27	18,41	3,18	75802,05	37901,03	30,30	30,68	7,21
2	26,89	2,19	155,14	32,93	-0,17	6,83	32,74	-1,23	0,59	18,18	3,26	91599,50	45799,75	3387,34	149,12	12,97
3	25,11	2,74	545,99	31,43	0,27	18,61	33,74	-1,55	0,28	18,40	3,18	76329,15	38164,58	14306,32	487,56	7,46
4	30,12	1,20	15,70	34,46	-0,61	2,47	33,63	-1,51	0,31	18,05	3,31	101517,23	50758,61	309,39	48,75	6,08
5	27,09	2,13	134,61	32,68	-0,09	8,08	33,77	-1,55	0,28	17,57	3,48	150504,51	75252,26	1788,81	107,43	3,72
6	31,73	0,70	5,01	35,45	-0,89	1,28	34,62	-1,82	0,15	18,03	3,31	103219,68	51609,84	97,12	24,83	2,91
7	23,94	3,10	1248,90	30,83	0,44	27,67	33,35	-1,42	0,38	18,15	3,27	93295,22	46647,61	26773,17	593,12	8,12
8	28,65	1,65	44,52	33,49	-0,33	4,71	34,97	-1,93	0,12	17,76	3,41	128435,58	64217,79	693,32	73,30	1,81
9	29,34	1,44	27,38	33,46	-0,32	4,82	33,21	-1,38	0,42	18,00	3,32	105370,78	52685,39	519,61	91,49	7,96
10	25,10	2,74	548,36	30,60	0,51	32,36	32,72	-1,22	0,60	19,33	2,85	35779,10	17889,55	30652,38	1808,98	33,49
11	29,05	1,52	33,47	33,70	-0,39	4,09	33,74	-1,55	0,28	20,31	2,51	16009,84	8004,92	4181,02	511,54	35,56
12	27,20	2,09	124,34	31,90	0,13	13,60	34,35	-1,74	0,18	18,11	3,28	96277,77	48138,88	2583,02	282,56	3,79
13	26,50	2,31	204,23	32,30	0,02	10,38	33,92	-1,60	0,25	18,43	3,17	74412,15	37206,07	5489,20	279,09	6,74
14	35,59	-0,48	0,33	35,87	-1,02	0,96	34,43	-1,76	0,17	19,28	2,87	37197,86	18598,93	17,63	51,87	9,24
15	36,06	-0,63	0,23	36,49	-1,19	0,64	33,32	-1,41	0,39	21,37	2,13	6770,31	3385,16	69,35	189,11	114,48
16	28,09	1,82	66,05	32,83	-0,13	7,33	34,93	-1,92	0,12	18,00	3,32	105370,78	52685,39	1253,61	139,10	2,26
17	28,25	1,77	59,26	32,76	-0,12	7,67	31,79	-0,93	1,18	16,40	3,89	388340,05	194170,02	305,21	39,48	6,09
18	27,05	2,14	138,29	31,90	0,13	13,60	33,37	-1,43	0,38	17,55	3,49	153003,54	76501,77	1807,70	177,79	4,91
19	33,61	0,12	1,33	34,80	-0,71	1,97	34,59	-1,82	0,15	17,68	3,44	136611,57	68305,79	19,48	28,86	2,24
20	27,66	1,95	89,77	32,91	-0,16	6,95	34,38	-1,75	0,18	18,46	3,16	72395,59	36197,79	2480,09	191,93	4,95
21	25,83	2,51	326,98	30,78	0,46	28,67	33,08	-1,34	0,46	15,49	4,22	820866,19	410433,10	796,68	69,86	1,13
22	28,71	1,63	42,71	33,43	-0,31	4,92	35,18	-2,00	0,10	17,38	3,54	174748,26	87374,13	488,77	56,28	1,14
23	25,78	2,53	338,60	30,84	0,44	27,59	34,29	-1,72	0,19	17,55	3,48	152145,28	76072,64	4450,95	362,69	2,51
24	28,27	1,76	58,10	33,59	-0,36	4,40	35,53	-2,11	0,08	17,76	3,41	128446,05	64223,02	904,73	68,58	1,20
25	32,70	0,40	2,53	35,40	-0,88	1,32	34,32	-1,73	0,19	18,99	2,97	47107,32	23553,66	107,60	56,09	7,91
26	27,43	2,02	105,32	31,93	0,13	13,36	30,57	-0,54	2,89	22,40	1,77	2936,58	1468,29	71728,50	9098,19	1970,46
27	27,69	1,94	88,09	33,67	-0,38	4,18	35,04	-1,96	0,11	18,66	3,09	61684,38	30842,19	2856,05	135,51	3,59
28	31,03	0,92	8,28	34,33	-0,57	2,69	34,48	-1,78	0,17	18,79	3,05	55481,44	27740,72	298,53	96,96	6,01
29	28,35	1,74	55,15	32,53	-0,05	8,96	31,32	-0,78	1,67	18,06	3,30	100291,54	50145,77	1099,74	178,67	33,26
30	28,68	1,64	43,44	32,63	-0,08	8,34	31,13	-0,72	1,92	17,99	3,33	106528,19	53264,10	815,62	156,65	36,14

31	29,20	1,48	30,21	33,43	-0,31	4,91	34,50	-1,79	0,16	18,44	3,17	73717,77	36858,88	819,56	133,24	4,45
32	33,43	0,18	1,51	35,29	-0,85	1,42	33,59	-1,50	0,32	20,24	2,53	17045,67	8522,84	177,46	166,84	37,50
33	21,95	3,71	5109,20	27,62	1,37	234,51	20,59	2,63	4223,71	20,47	2,45	14163,55	7081,78	721456,75	33114,26	596419,24
34	27,60	1,97	93,25	33,12	-0,22	6,03	34,56	-1,81	0,16	18,78	3,05	55981,22	27990,61	3331,54	215,56	5,59
35	26,65	2,26	183,71	31,07	0,37	23,57	30,16	-0,41	3,90	19,14	2,92	41613,69	20806,85	8829,32	1132,86	187,32
36	25,89	2,50	313,94	30,24	0,61	41,07	30,66	-0,57	2,72	19,08	2,94	43949,92	21974,96	14286,34	1868,87	123,55
37	29,00	1,54	34,69	32,05	0,09	12,28	31,41	-0,80	1,57	18,09	3,29	98028,12	49014,06	707,69	250,62	32,05
38	28,02	1,84	69,71	32,80	-0,13	7,46	33,86	-1,58	0,26	17,82	3,39	122563,86	61281,93	1137,49	121,75	4,28
39	25,80	2,53	335,71	31,87	0,14	13,86	33,90	-1,60	0,25	18,49	3,15	71137,71	35568,85	9438,25	389,61	7,13
40	29,35	1,43	27,15	33,06	-0,20	6,28	31,88	-0,95	1,11	17,99	3,33	106354,64	53177,32	510,59	118,16	20,88
41	28,60	1,66	46,22	33,65	-0,37	4,24	33,55	-1,48	0,33	17,40	3,54	172680,63	86340,31	535,28	49,06	3,80
42	27,22	2,09	122,40	33,52	-0,34	4,62	33,23	-1,38	0,41	17,36	3,55	178041,31	89020,65	1374,92	51,89	4,64
43	26,74	2,24	171,81	32,13	0,07	11,66	32,80	-1,25	0,57	18,11	3,29	96931,45	48465,72	3545,01	240,63	11,67
44	24,72	2,86	719,07	31,05	0,38	23,94	32,40	-1,12	0,76	18,25	3,24	86490,31	43245,16	16627,87	553,55	17,58
45	33,15	0,26	1,84	35,48	-0,90	1,25	32,97	-1,30	0,50	18,57	3,12	66229,13	33114,56	55,48	37,75	15,18
46	24,99	2,77	594,48	31,07	0,37	23,63	32,65	-1,20	0,63	18,71	3,07	59037,36	29518,68	20139,24	800,54	21,39
47	28,24	1,78	59,61	33,32	-0,28	5,27	34,93	-1,92	0,12	18,47	3,16	71760,97	35880,48	1661,31	146,95	3,33
48	31,00	0,93	8,45	33,58	-0,35	4,44	33,09	-1,34	0,46	17,36	3,55	178491,84	89245,92	94,67	49,73	5,16
49	33,18	0,26	1,80	35,54	-0,92	1,20	34,30	-1,72	0,19	17,61	3,46	145094,97	72547,48	24,80	16,61	2,61
50	32,25	0,54	3,48	35,66	-0,95	1,11	33,29	-1,40	0,40	19,14	2,92	41613,69	20806,85	167,29	53,35	19,11
51	26,13	2,42	265,44	31,31	0,30	20,08	32,86	-1,27	0,54	18,83	3,03	53644,50	26822,25	9896,12	748,48	20,23
52	33,17	0,26	1,82	35,58	-0,93	1,18	35,55	-2,12	0,08	19,69	2,73	26750,82	13375,41	135,78	87,94	5,68
53	27,81	1,91	80,59	33,02	-0,19	6,43	34,36	-1,74	0,18	19,64	2,74	27682,64	13841,32	5822,66	464,35	13,08
54	28,88	1,58	37,87	33,75	-0,40	3,96	32,34	-1,10	0,80	18,89	3,01	51271,38	25635,69	1477,20	154,32	31,07
55	27,70	1,94	87,43	32,77	-0,12	7,59	33,23	-1,38	0,41	18,81	3,04	54771,37	27385,69	3192,48	277,19	15,14
56	32,77	0,38	2,41	35,61	-0,94	1,15	35,24	-2,02	0,10	19,88	2,66	22874,84	11437,42	210,91	100,40	8,33
57	35,00	-0,30	0,50	35,00	-0,76	1,72	32,30	-1,09	0,82	21,17	2,20	7997,94	3998,97	124,26	431,30	204,78
58	32,11	0,59	3,85	35,49	-0,90	1,25	36,80	-2,51	0,03	18,82	3,04	54370,97	27185,49	141,68	45,85	1,13
59	31,29	0,84	6,85	36,09	-1,08	0,84	34,22	-1,70	0,20	22,88	1,60	1972,70	986,35	6947,66	847,50	204,11
60	30,98	0,93	8,58	34,50	-0,62	2,40	34,85	-1,90	0,13	18,96	2,98	48191,07	24095,54	356,20	99,72	5,28
61	33,62	0,12	1,32	35,74	-0,98	1,06	35,24	-2,02	0,10	18,79	3,05	55535,75	27767,87	47,70	38,05	3,43
62	30,78	0,99	9,87	34,29	-0,56	2,76	35,58	-2,13	0,07	19,37	2,84	34555,02	17277,51	571,50	159,83	4,30
63	30,30	1,14	13,89	33,66	-0,38	4,21	35,00	-1,94	0,11	19,45	2,81	32294,19	16147,09	860,16	260,99	7,05
64	30,79	0,99	9,78	34,20	-0,53	2,94	35,07	-1,97	0,11	19,59	2,76	29023,04	14511,52	673,72	202,85	7,45
65	29,53	1,38	23,90	33,54	-0,34	4,55	34,49	-1,78	0,16	19,52	2,79	30527,72	15263,86	1566,02	298,26	10,78
66	32,72	0,40	2,50	34,72	-0,68	2,07	35,05	-1,96	0,11	18,52	3,14	69209,89	34604,94	72,32	59,93	3,18
67	32,96	0,32	2,11	35,24	-0,83	1,47	34,89	-1,91	0,12	18,68	3,08	60548,40	30274,20	69,60	48,49	4,07
68	32,61	0,43	2,71	35,38	-0,87	1,34	35,63	-2,14	0,07	18,06	3,30	100783,32	50391,66	53,73	26,51	1,43
69	32,64	0,42	2,64	35,13	-0,80	1,58	36,08	-2,29	0,05	18,11	3,29	96994,69	48497,34	54,38	32,59	1,06
70	32,98	0,32	2,07	35,85	-1,01	0,98	35,15	-1,99	0,10	18,16	3,27	92968,74	46484,37	44,54	21,10	2,19
71	33,03	0,30	2,01	35,64	-0,95	1,13	35,01	-1,95	0,11	18,53	3,14	68536,11	34268,05	58,60	32,91	3,29
72	31,40	0,80	6,35	34,49	-0,62	2,43	35,42	-2,08	0,08	18,25	3,24	86321,25	43160,62	147,19	56,19	1,94
73	34,14	-0,04	0,91	35,66	-0,95	1,11	35,76	-2,18	0,07	18,34	3,20	80149,07	40074,53	22,78	27,74	1,63
74	31,84	0,67	4,65	32,81	-0,13	7,43	33,61	-1,50	0,31	16,01	4,03	537364,15	268682,07	17,32	27,64	1,17
75	33,03	0,30	2,01	33,65	-0,37	4,23	34,17	-1,68	0,21	16,26	3,94	435788,65	217894,32	9,21	19,41	0,96
76	31,51	0,77	5,88	32,21	0,04	11,04	33,11	-1,34	0,45	19,13	2,93	42228,85	21114,43	278,54	523,00	21,40
77	33,68	0,10	1,26	33,31	-0,27	5,33	34,39	-1,75	0,18	18,18	3,26	91316,17	45658,08	27,68	116,75	3,89
78	32,32	0,52	3,31	33,59	-0,35	4,42	31,90	-0,96	1,10	18,51	3,14	69605,98	34802,99	95,04	127,04	31,54
79	34,49	-0,15	0,71	34,76	-0,69	2,02	34,44	-1,77	0,17	17,75	3,41	129761,68	64880,84	11,01	31,19	2,63
81	33,60	0,13	1,34	34,20	-0,53	2,95	33,54	-1,48	0,33	17,66	3,45	139526,78	69763,39	19,16	42,22	4,74

82	35,87	-0,57	0,27	34,81	-0,71	1,96	35,79	-2,19	0,06	18,22	3,25	88098,63	44049,31	6,08	44,43	1,45
83	33,57	0,14	1,37	35,63	-0,94	1,14	35,12	-1,98	0,10	17,85	3,38	119797,80	59898,90	22,80	18,98	1,74
84	33,22	0,24	1,76	34,89	-0,73	1,86	32,97	-1,30	0,50	17,89	3,36	115765,30	57882,65	30,33	32,15	8,68
85	34,67	-0,20	0,63	36,25	-1,12	0,75	33,85	-1,58	0,26	18,45	3,17	73453,81	36726,91	17,14	20,47	7,17
86	35,26	-0,38	0,41	34,96	-0,75	1,77	38,81	-3,15	0,01	18,41	3,18	75456,77	37728,39	10,96	46,95	0,19
87	33,49	0,16	1,45	34,98	-0,76	1,75	33,74	-1,55	0,29	19,22	2,89	38986,27	19493,13	74,39	89,97	14,62
88	33,78	0,07	1,18	34,00	-0,48	3,35	32,89	-1,27	0,53	17,88	3,37	116522,79	58261,40	20,17	57,49	9,14
89	31,36	0,82	6,54	35,00	-0,76	1,72	36,19	-2,32	0,05	21,78	1,99	4844,32	2422,16	2700,08	712,08	19,73
90	32,72	0,40	2,50	35,56	-0,93	1,19	35,20	-2,01	0,10	18,63	3,10	63273,49	31636,75	79,16	37,50	3,11
91	32,84	0,36	2,30	35,27	-0,84	1,44	34,68	-1,84	0,14	19,89	2,65	22560,00	11280,00	203,95	127,92	12,70
92	30,34	1,13	13,48	33,82	-0,42	3,77	35,27	-2,03	0,09	19,89	2,65	22560,00	11280,00	1195,23	334,52	8,25
93	33,44	0,18	1,50	35,48	-0,90	1,25	35,05	-1,96	0,11	21,04	2,25	8899,37	4449,69	337,15	281,78	24,59
94	31,22	0,86	7,20	34,35	-0,58	2,66	37,56	-2,76	0,02	19,91	2,65	22195,14	11097,57	648,42	239,25	1,58
95	31,45	0,79	6,14	34,93	-0,74	1,81	35,17	-2,00	0,10	19,95	2,63	21483,03	10741,52	571,28	168,45	9,32
96	31,10	0,89	7,84	34,65	-0,66	2,18	35,70	-2,17	0,07	19,94	2,64	21694,23	10847,12	722,71	201,40	6,27
97	30,69	1,02	10,52	34,81	-0,71	1,95	35,30	-2,04	0,09	19,10	2,94	43098,36	21549,18	488,35	90,71	4,24
98	33,76	0,08	1,20	35,48	-0,90	1,26	31,00	-0,68	2,11	18,44	3,17	73874,19	36937,10	32,41	34,01	57,01
99	34,63	-0,19	0,64	34,90	-0,73	1,84	34,81	-1,88	0,13	17,80	3,40	124375,68	62187,84	10,37	29,62	2,10
100	28,79	1,60	40,19	32,53	-0,05	8,96	32,00	-0,99	1,02	18,20	3,25	89912,61	44956,30	893,96	199,30	22,68
101	28,95	1,56	36,09	32,34	0,01	10,17	33,15	-1,36	0,44	18,43	3,17	74418,22	37209,11	969,91	273,26	11,80
102	33,32	0,21	1,64	35,06	-0,78	1,66	35,54	-2,11	0,08	17,43	3,53	167754,65	83877,33	19,50	19,82	0,92
103	33,78	0,07	1,18	35,70	-0,97	1,08	34,04	-1,64	0,23	18,21	3,25	88819,79	44409,89	26,50	24,39	5,18
104	31,07	0,91	8,04	33,40	-0,30	5,02	34,60	-1,82	0,15	18,06	3,30	100701,19	50350,59	159,71	99,71	3,03
105	19,53	4,45	28235,65	23,90	2,45	2793,99	17,92	3,47	29681,25	16,92	3,71	254447,37	127223,68	221937,04	21961,20	233299,71
HeLa-C2 Positive c	19,23	4,54	34990,12	18,98	3,87	73955,74	31,88	-0,95	1,11	19,46	2,81	32267,87	16133,94	2168727,98	4583862,45	68,88
