# Supplementary material for: Active HHV-6 Infection of Cerebellar Purkinje Cells in Mood Disorders
Source: Front Microbiol. 2018 Aug 21;9:1955. doi: 10.3389/fmicb.2018.01955 (PMC6110891; doi:10.3389/fmicb.2018.01955)
Supplement: TABLE S6 — Summary of results from various types of tests carried out on both the cohort of samples. [file Data_Sheet_6.PDF]

Table S6: Summary of results from various types of tests carried out on both the cohort of samples.

X CODE	HHV-6 DNA (PCR)	HHV-6A DNA (PCR)	HHV-6B DNA (PCR)	HHV-6A protein (IFA)	HHV-6B protein (IFA)	FISH	TEM
487	Positive	Negative	Positive	Negative	Negative	-	-
484	Positive	Positive	Negative	Positive	Positive	-	-
482	Positive	Negative	Positive	Positive	Positive	-	-
470	Negative	Negative	Negative	Negative	Negative	Negative	-
467	Positive	Positive	Positive	Positive	Positive	Positive	Positive
466	Positive	Positive	Positive	Positive	Positive	-	-
462	Positive	Positive	Negative	Positive	Positive	-	-
459	Positive	Positive	Positive	Negative	Negative	-	-
457	Positive	Positive	Negative	Negative	Positive	-	-
454	Positive	Positive	Positive	Positive	Negative	Positive	Positive
453	Negative	Negative	Negative	Negative	Negative	Positive	-
452	Positive	Positive	Positive	Negative	Positive	-	-
451	Positive	Positive	Negative	Negative	Positive	-	-
441	Positive	Positive	Negative	Negative	Positive	-	-
440	Positive	Positive	Positive	Positive	Positive	-	-
436	Positive	Positive	Positive	Negative	Negative	-	-
434	Positive	Positive	Negative	Positive	Positive	Positive	Positive
432	Positive	Positive	Positive	Positive	Positive	-	-
430	Positive	Positive	Positive	Positive	Negative	-	-
428	Positive	Positive	Positive	Negative	Positive	-	-
427	Positive	Positive	Positive	Negative	Negative	-	-
425	Positive	Positive	Positive	Positive	Positive	-	-
419	Negative	Negative	Negative	Positive	Positive	Positive	Positive
418	Positive	Negative	Positive	Positive	Negative	-	-
417	Positive	Positive	Positive	Positive	Positive	-	-
415	Negative	Negative	Negative	Positive	Positive	-	-

412	Positive	Positive	Positive	Positive	Negative	Positive	Positive
411	Positive	Positive	Negative	Negative	Negative	-	-
409	Positive	Positive	Negative	Positive	Positive	-	-
408	Positive	Positive	Negative	Positive	Positive	-	-
407	Positive	Positive	Positive	Positive	Positive	-	-
401	Positive	Positive	Positive	Positive	Positive	-	-
400	Positive	Positive	Negative	Positive	Negative	-	-
399	Positive	Positive	Positive	Negative	Positive	-	-
397	Positive	Positive	Positive	Negative	Negative	-	-
396	Positive	Negative	Positive	Positive	Positive	Positive	-
384	Positive	Negative	Positive	Negative	Negative	-	-
382	Negative	Negative	Negative	Negative	Negative	-	-
380	Positive	Positive	Positive	Negative	Positive	-	-
377	Negative	Negative	Negative	Negative	Negative	-	-
376	Negative	Negative	Negative	Positive	Negative	-	-
374	Positive	Positive	Negative	Negative	Positive	-	-
372	Positive	Positive	Positive	Negative	Negative	-	-
365	Positive	Positive	Negative	Positive	Negative	-	-
364	Positive	Negative	Positive	Negative	Negative	-	-
362	Positive	Positive	Positive	Negative	Positive	-	-
359	Negative	Negative	Negative	Positive	Negative	-	-
353	Positive	Positive	Negative	Positive	Negative	Positive	-
351	Positive	Negative	Positive	Negative	Negative	-	-
344	Positive	Positive	Positive	Positive	Positive	-	-
342	Negative	Negative	Negative	Positive	Positive	Negative	-
338	Positive	Positive	Negative	Negative	Positive	-	-
337	Positive	Positive	Positive	Positive	Positive	-	-
335	Positive	Negative	Positive	Negative	Negative	-	-
332	Positive	Positive	Negative	Positive	Positive	-	-
329	Positive	Positive	Positive	Positive	Positive	-	-
328	Negative	Negative	Negative	Negative	Negative	-	-
327	Positive	Positive	Negative	Positive		-	-

326	Negative	Negative	Negative	Positive	Positive	-	-
321	Negative	Negative	Negative	Negative	Positive	-	-
165	Negative	Negative	Negative	Positive	Positive	-	-
24	Positive	Positive	Negative	Negative	Negative	Positive	-
167	Positive	Positive	Negative	Negative	Negative	-	-
32	Positive	Positive	Negative	Negative	Positive	-	-
172	Positive	Positive	Negative	Positive	Negative	-	-
35	Negative	Negative	Negative	Negative	Negative	-	-
174	Positive	Positive	Negative	Negative	Negative	-	-
44	Positive	Positive	Negative	Positive	Negative	-	-
179	Positive	Positive	Negative	Negative	Negative	Positive	-
48	Positive	Positive	Negative	Negative	Positive	-	-
186	Positive	Positive	Negative	Positive	Negative	-	-
49	Positive	Positive	Negative	Negative	Negative	-	-
194	Positive	Positive	Negative	Negative	Negative	-	-
50	Negative	Negative	Negative	Positive	Negative	Negative	-
198	Negative	Negative	Negative	Positive	Negative	-	-
51	Positive	Positive	Negative	Negative	Positive	-	-
201	Positive	Positive	Negative	Negative	Positive	-	-
53	Positive	Positive	Negative	Negative	Negative	-	-
202	Negative	Negative	Negative	Positive	Negative	-	-
54	Positive	Positive	Negative	Negative	Negative	-	-
209	Positive	Positive	Negative	Negative	Positive	-	-
58	Positive	Positive	Negative	Negative	Negative	-	-
210	Positive	Positive	Negative	Negative	Negative	Positive	-
59	Positive	Positive	Negative	Negative	Positive	-	-
211	Negative	Negative	Negative	Negative	Negative	Negative	Negative
60	Positive	Positive	Positive	Negative	Negative	-	-
212	Positive	Positive	Negative	Negative	Negative	-	-
61	Negative	Negative	Negative	Positive	Positive	-	-
214	Positive	Positive	Negative	Negative	Negative	-	-
62	Positive	Positive	Negative	Negative	Negative	-	-

220	Positive	Positive	Negative	Negative	Positive	-	-
67	Negative	Negative	Negative	Negative	Negative	Negative	Negative
230	Positive	Positive	Positive	Negative	Negative	-	-
68	Positive	Positive	Negative	Negative	Negative	-	-
232	Positive	Positive	Positive	Negative	Negative	-	-
72	Positive	Positive	Positive	Negative	Negative	-	-
238	Positive	Positive	Negative	Negative	Negative	Positive	-
76	Positive	Positive	Negative	Negative	Negative	-	-
78	Positive	Positive	Negative	Negative	Negative	Positive	-
245	Positive	Positive	Negative	Negative	Negative	-	-
79	Positive	Positive	Negative	Negative	Negative	-	-
256	Positive	Positive	Negative	Negative	Positive	-	-
85	Positive	Positive	Negative	Negative	Negative	Positive	-
262	Negative	Negative	Negative	Negative	Positive	-	-
86	Positive	Positive	Negative	Negative	Negative	-	-
265	Positive	Positive	Negative	Negative	Positive	-	-
88	Negative	Negative	Negative	Negative	Negative	-	-
269	Negative	Negative	Negative	Negative	Negative	-	-
89	Negative	Negative	Negative	Negative	Positive	-	-
271	Positive	Positive	Negative	Negative	Negative	-	-
91	Negative	Negative	Negative	Negative	Negative	-	-
273	Positive	Positive	Negative	Negative	Negative	Positive	-
96	Positive	Positive	Negative	Negative	Negative	-	-
274	Positive	Positive	Negative	Negative	Negative	Positive	-
99	Negative	Negative	Negative	Negative	Negative	-	-
276	-	-	-	Negative	Positive	-	-
100	Negative	Negative	Negative	Negative	Negative	-	-
278	Negative	Negative	Positive	Negative	Negative	-	-
101	Negative	Negative	Negative	Negative	Negative	-	-
283	Negative	Negative	Negative	Negative	Positive	-	-
105	Positive	Positive	Negative	Negative	Positive	-	-
287	Positive	Positive	Negative	Positive	Negative	-	-

107	Positive	Positive	Negative	Positive	Negative	-	-
288	Positive	Positive	Negative	Negative	Negative	Positive	-
108	Negative	Negative	Negative	Positive	Negative	-	-
296	Negative	Negative	Negative	Negative	Negative	-	-
109	Negative	Negative	Negative	Negative	Positive	-	-
297	Negative	Negative	Negative	Positive	Positive	-	-
113	Negative	Negative	Negative	Negative	Negative	-	-
299	Negative	Negative	Negative	Positive	Negative	-	-
119	Negative	Negative	Negative	Negative	Positive	-	-
301	Negative	Negative	Negative	Negative	Positive	-	-
120	Negative	Negative	Negative	Negative	Negative	-	-
302	Negative	Negative	Negative	Negative	Negative	-	-
123	Negative	Negative	Negative	Negative	Negative	-	-
307	Negative	Negative	Negative	Positive	Negative	-	-
127	Negative	Negative	Negative	Positive	Positive	-	-
308	Negative	Negative	Negative	Negative	Negative	-	-
131	-	-	-	Positive	Negative	-	-
311	Negative	Negative	Negative	Negative	Positive	-	-
132	Negative	Negative	Negative	Positive	Negative	-	-
313	Negative	Negative	Negative	Negative	Positive	-	-
135	Negative	Negative	Negative	Positive	Negative	-	-
325	Negative	Negative	Negative	Negative	Positive	-	-
139	Negative	Negative	Negative	Negative	Negative	-	-
330	Negative	Negative	Negative	Negative	Positive	-	-
141	Negative	Negative	Negative	Negative	Negative	-	-
333	Negative	Negative	Negative	Negative	Negative	-	-
143	Negative	Negative	Negative	Negative	Negative	-	-
345	Negative	Negative	Negative	Positive	Negative	-	-
144	Positive	Positive	Negative	Positive	Negative	-	-
367	Negative	Negative	Negative	Positive	Negative	-	-
146	Negative	Negative	Negative	Negative	Positive	-	-
381	Negative	Negative	Negative	Positive	Negative	-	-

150	Negative	Negative	Negative	Negative	Negative	-	-
387	Positive	Positive	Negative	Negative	Negative	Positive	-
153	Negative	Negative	Negative	Negative	Positive	-	-
389	Negative	Negative	Negative	Positive	Negative	-	-
158	Positive	Positive	Negative	Positive	Negative	-	-
390	Positive	Positive	Negative	Positive	Negative	Positive	-
160	Negative	Negative	Negative	Negative	Negative	-	-
458	Negative	Negative	Negative	Negative	Positive	-	-
164	Negative	Negative	Negative	Positive	Positive	-	-
461	Positive	Positive	Positive	Positive	Positive	Positive	-
