# Supplementary material for: Active HHV-6 Infection of Cerebellar Purkinje Cells in Mood Disorders
Source: Front Microbiol. 2018 Aug 21;9:1955. doi: 10.3389/fmicb.2018.01955 (PMC6110891; doi:10.3389/fmicb.2018.01955)
Supplement: TABLE S7 — Summary of gene set enrichment analysis (GSEA) analysis showing significantly enriched pathways. [file Data_Sheet_7.pdf]

Table S7. Summary of gene set enrichment analysis (GSEA) analysis showing significantly enriched pathways.

Pathway Analysis	GSEA		Cutoff: q-value <= 0.05				
ID	Description	setSize	enrichmentScore	NES	pvalue	qvalues	coreEnrichment
390522	Striated Muscle Contraction	33	0,4830	1,6739	0,0018	0,0038	7273/4624/4632/7136/88/7171/4607/4703/89/4621/58/70/4635/7137/7140/4633/7135/1756/7139/4604/7170/4626/1674
375280	Amine ligand-binding receptors	36	0,5247	1,8453	0,0019	0,0038	150/3354/151/155/1129/1128/154/3360/3351/1812/59340/3352/3269/3358/1813
2555396	Mitotic Metaphase and Anaphase	156	-0,3677	-1,6825	0,0019	0,0038	5685/29882/5694/7324/5691/5700/26271/51434/5690/5695/5716/5717/5704/23332/23141/2491/5689/79980/5688/79902/10726/8881/5709/55839/8697/5707/79023/55166/5683/246184/5708/55055/6396/5692/80152/10213/5903/10197/54908/5518/55746/5682/1060/5684/4928/5693/9861/5718/5714/5705/7314/9183/23279/5687/91687/5905/23421/81929/5347/10393/401541/5702/5686/64682/2010/6233/9735/5706/5721/7443/5719
5653656	Vesicle-mediated transport	171	-0,3462	-1,6066	0,0019	0,0038	11267/91782/372/10484/9698/9847/811/11336/949/11316/9525/808/801/7532/11127/162/8027/27183/27243/9276/23166/338/55738/8907/8906/84062/134829/5564/7531/137492/3312/10640/51422/22818/149371/8775/9632/164/51028/1282/10717/1211/23216/6396/5571/23431/805/2706/11154/54536/51552/8773/208/3263/7251/51510/9685/5872/7314/84313/10802/2512/2495/3320/3250/81035/6233/3240/1278/6678/348/1601/10808/2697/9332/3043
74159	Transcription	146	-0,6106	-2,7630	0,0019	0,0038	904/10914/27297/134353/2962/6917/7307/905/84265/2963/5438/9295/6877/4331/5451/221830/2068/11338/10189/55703/9785/6429/9329/4116/2965/64425/5595/6882/9328/9330/10978/8683/10622/83860/1479/4686/6924/53981/7270/6923/11051/51692/6884/6829/5434/171568/10302/65109/6873/6628/2071/51585/2960/1477/5437/6430/6426/8732/6617/9014/10623/2968/6883/6428/10621/22916/51728/64216/5435/5440/84172/5432/6427/2961/2958/7978/5436/25885/6921/2971/6431/25888/9015/51082/6636/7019/54700/6432/1022/9533/6749/7702/6637/30834/6635
975956	Nonsense Mediated Decay (NMD) independent of the Exon Junction Complex (EJC)	74	-0,5343	-2,1499	0,0019	0,0038	11224/6235/6169/6154/1981/6187/6138/6227/4686/2107/6234/6201/9045/6136/6160/5976/6157/6181/22916/6133/6229/6189/6137/6175/6202/6228/6152/6233/6207/6171/6135/6134/6192
176408	Regulation of APC/C activators between G1/S and early anaphase	74	-0,5604	-2,2547	0,0019	0,0038	5685/29882/5694/7324/5691/5700/26271/51434/5690/5695/5716/5717/5704/5689/5688/8881/5709/8697/5707/6500/5683/246184/5708/5692/10213/891/10197/8454/5682/5684/5693/9861/5718/5714/5705/7314/5687/5347/10393/5702/5686/64682/6233/5706/5721/5719
2990846	SUMOylation	74	-0,4976	-2,0019	0,0019	0,0038	1911/79902/10762/9631/9972/53371/29843/165918/79023/59343/10054/8086/10055/6996/6015/56160/9818/23511/5903/55746/286053/4928/129401/23165/641/8480/23279/493861/81929/23137/7329/7341
212436	Generic Transcription Pathway	182	-0,3594	-1,6748	0,0020	0,0038	2744/7068/6257/5915/904/9377/64750/9968/905/4089/112950/9443/5564/7531/1024/22938/27244/6667/5933/27165/51422/9167/7596/29959/5469/51003/23327/7323/6256/2821/1327/54205/9439/10155/10025/54778/50484/7003/5571/9975/7004/7157/1351/7067/1490/4088/84246/892/9440/7181/25937/4087/29079/7314/2936/1329/10413/10001/29982/1350/25824/1349/6233/9477/6009/90390/4929/3726/4853/54541

913531	Interferon Signaling	153	-0,4988	-2,2646	0,0020	0,0038	3840/815/10762/7334/9631/6772/1981/9972/53371/3437/3837/5595/79023/9246/8086/10437/3430/11274/3117/85363/9818/3839/23511/3459/5903/4600/55746/3454/3841/684/3383/6737/4928/129401/23165/5771/8480/3838/55128/7314/3717/23279/51447/3661/81929/389898/3659/4281/5300/3460/7341/1974/6233/25939/3115/567/5335/3113/10346/9775/3664/2634/10379/3394/10410/7412/8519/1958/10581/4502
156842	Eukaryotic Translation Elongation	72	-0,5178	-2,0746	0,0020	0,0038	11224/6235/6169/6154/6187/6138/6227/1937/6234/6201/9045/6136/6160/1915/6157/6181/6133/6229/6189/6137/6175/6202/6228/6152/6233/6207/6171/6135/6134/6192
73894	DNA Repair	133	-0,4047	-1,8122	0,0020	0,0038	56852/83932/6917/5429/4437/8451/2237/5438/10973/4331/5423/2068/2175/5985/1161/2965/5111/5427/9246/6996/7515/7353/10459/9100/2956/2072/1642/56949/5434/2547/2189/7415/2071/4683/5437/2968/4436/9978/84164/7314/10714/5435/5440/5432/25898/57697/4361/29089/5436/5982/328/6233/5984/1022/55215/5426
5610787	Hedgehog 'off' state	77	-0,5062	-2,0530	0,0020	0,0038	5685/5694/5691/5700/5573/8650/5690/5695/5727/5716/5717/5704/1452/9742/5689/5576/5688/79809/5709/5707/57728/6500/5683/5708/5692/10213/2735/10197/8454/5682/5684/5693/9861/9978/5718/5714/5705/7314/5687/5702/5686/57539/6233/5706/5721/5719
4086400	PCP/CE pathway	77	-0,4586	-1,8600	0,0020	0,0038	5685/5694/409/5691/5700/5690/5695/64750/161/5716/5717/5704/5216/6259/5689/5688/5709/5707/5683/5708/5692/10213/1856/10197/160/5682/5684/5693/9861/5718/5714/5705/7314/5687/5702/5686/5879/6233/7474/5706/5721/5719
176814	Activation of APC/C and APC/C:Cdc20 mediated degradation of mitotic proteins	70	-0,5374	-2,1380	0,0020	0,0038	5685/29882/5694/7324/5691/5700/51434/5690/5695/5716/5717/5704/5689/5688/8881/5709/8697/5707/5683/246184/5708/5692/10213/891/10197/5682/5684/5693/9861/5718/5714/5705/7314/5687/5347/10393/5702/5686/64682/6233/5706/5721/5719
72689	Formation of a pool of free 40S subunits	78	-0,5190	-2,1061	0,0020	0,0038	6130/8667/6146/6156/3646/8668/6155/6125/8664/8669/6194/11224/6235/6169/6154/6187/8666/6138/6227/6234/6201/9045/8661/6136/6160/6157/6181/8662/6133/6229/6189/6137/6175/6202/6228/6152/6233/6207/6171/6135/6134/6192
69304	Regulation of DNA replication	70	-0,5423	-2,1577	0,0020	0,0038	5685/5694/5691/5700/5690/5695/5716/5717/5704/5689/5925/5688/5709/23595/5707/5683/5708/5692/4175/4176/10213/51053/4999/10197/5682/5684/5693/9861/5718/5714/5705/7314/5687/84515/5001/5702/5686/6233/5706/5721/1027/990/5719
69300	Removal of licensing factors from origins	70	-0,5423	-2,1577	0,0020	0,0038	5685/5694/5691/5700/5690/5695/5716/5717/5704/5689/5925/5688/5709/23595/5707/5683/5708/5692/4175/4176/10213/51053/4999/10197/5682/5684/5693/9861/5718/5714/5705/7314/5687/84515/5001/5702/5686/6233/5706/5721/1027/990/5719
72172	mRNA Splicing	107	-0,6381	-2,7585	0,0020	0,0038	10914/1660/10946/2962/57819/7307/6625/988/2963/11100/5438/9295/9128/3312/10236/57794/11338/10189/3192/9785/6429/4116/5725/10978/8683/84844/1479/4686/53981/83443/23450/5093/11051/51692/3187/5434/56259/9416/65109/3181/6628/3190/9343/51585/6626/1477/5437/6430/6426/10907/27339/6428/22916/6627/5435/5440/4670/5432/4809/6427/5436/51503/10286/6431/24148/6636/6632/6432/3178/6637/6629/6635
72163	mRNA Splicing - Major Pathway	107	-0,6381	-2,7585	0,0020	0,0038	10914/1660/10946/2962/57819/7307/6625/988/2963/11100/5438/9295/9128/3312/10236/57794/11338/10189/3192/9785/6429/4116/5725/10978/8683/84844/1479/4686/53981/83443/23450/5093/11051/51692/3187/5434/56259/9416/65109/3181/6628/3190/9343/51585/6626/1477/5437/6430/6426/10907/27339/6428/22916/6627/5435/5440/4670/5432/4809/6427/5436/51503/10286/6431/24148/6636/6632/6432/3178/6637/6629/6635

72737	Cap-dependent Translation Initiation	95	-0,5180	-2,1994	0,0020	0,0038	6130/8667/6146/6156/3646/8668/6155/6125/1965/8664/8669/6194/11224/6235/6169/6154/1981/8894/6187/8666/6138/6227/6234/6201/9045/8661/1975/8892/6136/6160/8891/8890/6157/6181/8662/6133/1983/6229/6189/6137/6175/8893/6202/6228/1974/6152/6233/6207/6171/6135/6134/6192
5607764	CLEC7A (Dectin-1) signaling	95	-0,5084	-2,1587	0,0020	0,0038	808/801/5713/5685/5694/5691/5700/5532/3709/5530/4775/5690/5336/5695/5170/5716/5717/5704/7322/5689/5534/5688/23118/10892/7334/6885/5709/23291/5707/6500/4790/5683/5708/5692/10213/805/10197/4773/8454/5682/5970/5684/5693/3551/9861/5718/5714/5705/7314/5687/1147/389898/5702/5686/6233/5706/5721/5719/4792
72613	Eukaryotic Translation Initiation	95	-0,5180	-2,1994	0,0020	0,0038	6130/8667/6146/6156/3646/8668/6155/6125/1965/8664/8669/6194/11224/6235/6169/6154/1981/8894/6187/8666/6138/6227/6234/6201/9045/8661/1975/8892/6136/6160/8891/8890/6157/6181/8662/6133/1983/6229/6189/6137/6175/8893/6202/6228/1974/6152/6233/6207/6171/6135/6134/6192
168255	Influenza Life Cycle	96	-0,5544	-2,3553	0,0020	0,0038	6194/11224/6235/79902/10762/6169/6154/9631/9972/53371/3837/79023/6187/8086/6138/6227/9818/23511/6234/6201/9045/5903/6136/55746/6160/6157/6181/4928/129401/23165/8480/6133/23279/5901/6229/6189/81929/6137/6175/6202/6228/6152/6233/6207/6171/6135/6134/6192
168273	Influenza Viral RNA Transcription and Replication	92	-0,5573	-2,3492	0,0020	0,0038	6194/11224/6235/79902/10762/6169/6154/9631/9972/53371/79023/6187/8086/6138/6227/9818/23511/6234/6201/9045/5903/6136/55746/6160/6157/6181/4928/129401/23165/8480/6133/23279/6229/6189/81929/6137/6175/6202/6228/6152/6233/6207/6171/6135/6134/6192
162599	Late Phase of HIV Life Cycle	114	-0,5582	-2,4327	0,0020	0,0038	5045/8178/27183/27243/904/2962/6917/905/2963/5438/5902/6877/137492/4927/4331/79902/2068/10762/9631/9972/53371/2965/23327/6882/79023/8086/83860/4686/6924/6923/6884/9818/23511/6829/5434/5903/6873/55746/2071/2960/4836/5437/8732/4928/2968/7251/129401/23165/6883/51510/22916/8480/7314/23279/5435/5440/5901/5432/81929/2961/2958/5436/6921/6233/1022/6749
975957	Nonsense Mediated Decay (NMD) enhanced by the Exon Junction Complex (EJC)	90	-0,5274	-2,2092	0,0020	0,0038	11224/6235/6169/6154/1981/4116/23381/6187/6138/6227/4686/2107/6234/6201/9045/65109/6136/5518/6160/5976/6157/6181/22916/6133/6229/6189/6137/6175/6202/6228/6152/6233/55181/6207/9775/6171/6135/6134/6192
927802	Nonsense-Mediated Decay (NMD)	90	-0,5274	-2,2092	0,0020	0,0038	11224/6235/6169/6154/1981/4116/23381/6187/6138/6227/4686/2107/6234/6201/9045/65109/6136/5518/6160/5976/6157/6181/22916/6133/6229/6189/6137/6175/6202/6228/6152/6233/55181/6207/9775/6171/6135/6134/6192
450531	Regulation of mRNA stability by proteins that bind AU-rich elements	84	-0,5434	-2,2408	0,0020	0,0038	5713/5685/5694/5691/5700/5393/5690/5695/22894/5716/5717/5704/5689/3312/5688/1981/5709/5707/6418/5683/5708/5692/10213/10197/1432/5682/23016/5684/23404/5693/51013/5578/9861/3315/5718/5714/5705/7314/5687/7538/5702/5686/167227/6233/5706/5721/51010/5719
69239	Synthesis of DNA	95	-0,5325	-2,2606	0,0020	0,0038	5685/5694/9837/5691/5700/6119/5690/5695/5716/5717/5704/2237/5689/5925/5688/5985/5709/1763/23595/5111/5427/5707/5683/5708/5692/4175/4176/10213/4999/5558/10197/5682/5684/5693/9861/5718/5714/5705/7314/10714/5687/84515/5001/5702/5686/5982/328/6233/5984/5706/5721/1027/990/5719/5426
157279	3' -UTR-mediated translational regulation	87	-0,5138	-2,1362	0,0020	0,0038	6130/8667/6146/6156/3646/8668/6155/6125/1965/8664/8669/6194/11224/6235/6169/6154/1981/8894/6187/8666/6138/6227/6234/6201/9045/8661/1975/6136/6160/6157/6181/8662/6133/6229/6189/6137/6175/6202/6228/1974/6152/6233/6207/6171/6135/6134/6192
4641257	degradation of AXIN	54	-0,5846	-2,2189	0,0020	0,0038	5685/5694/5691/5700/5690/5695/64750/5716/5717/5704/5689/5688/5709/5707/5683/5708/5692/10213/81847/10197/5682/5684/5693/9861/5718/5714/5705/7314/5687/5702/5686/6233/5706/5721/5719

4641258	degradation of DVL	55	-0,5633	-2,1425	0,0020	0,0038	59349/5713/5685/5694/5691/5700/5690/5695/5716/5717/5704/5689/5688/5709/5707/51339/5683/5708/5692/10213/1856/10197/5682/5684/5693/9861/9978/5718/5714/5705/7314/5687/5702/5686/6233/5706/5721/5719
5387390	Hh mutants abrogate ligand secretion	55	-0,5776	-2,1971	0,0020	0,0038	5713/5685/5694/5691/5700/5690/5695/10956/5716/5717/5704/5689/5688/5709/5707/51009/5683/5708/5692/10213/10197/7415/5682/5684/5693/9861/5718/5714/5705/7314/5687/5702/5686/6233/5706/5721/5719
5362768	Hh mutants that don't undergo autocatalytic processing are degraded by ERAD	55	-0,5776	-2,1971	0,0020	0,0038	5713/5685/5694/5691/5700/5690/5695/10956/5716/5717/5704/5689/5688/5709/5707/51009/5683/5708/5692/10213/10197/7415/5682/5684/5693/9861/5718/5714/5705/7314/5687/5702/5686/6233/5706/5721/5719
156827	L13a-mediated translational silencing of Ceruloplasmin expression	87	-0,5138	-2,1362	0,0020	0,0038	6130/8667/6146/6156/3646/8668/6155/6125/1965/8664/8669/6194/11224/6235/6169/6154/1981/8894/6187/8666/6138/6227/6234/6201/9045/8661/1975/6136/6160/6157/6181/8662/6133/6229/6189/6137/6175/6202/6228/1974/6152/6233/6207/6171/6135/6134/6192
5368287	Mitochondrial translation	87	-0,5534	-2,3008	0,0020	0,0038	84545/128308/51073/3396/60488/63931/51021/65993/116540/51258/23107/51253/51069/57129/64965/65005/29088/28973/64976/51650/740/64960/118487/10884/54534/9553/123263/56945/124995/28998/54148/219927/55173/10240/64949/55037/7284/10102/92259/55052/65080/85476/51318/65008/29093/51023/26589/29074/6183/51649/28957
68949	Orc1 removal from chromatin	68	-0,5602	-2,2178	0,0020	0,0038	5685/5694/5691/5700/5690/5695/5716/5717/5704/5689/5925/5688/5709/23595/5707/5683/5708/5692/4175/4176/10213/4999/10197/5682/5684/5693/9861/5718/5714/5705/7314/5687/84515/5001/5702/5686/6233/5706/5721/1027/990/5719
69563	p53-Dependent G1 DNA Damage Response	55	-0,5969	-2,2702	0,0020	0,0038	5685/5694/5691/5700/5690/5695/5716/5717/5704/64326/5689/5688/5709/5707/5683/5708/5692/10213/7157/10197/5682/5684/5693/9861/5718/5714/5705/7314/5687/5702/5686/6233/5706/5721/1027/5719
69580	p53-Dependent G1/S DNA damage checkpoint	55	-0,5969	-2,2702	0,0020	0,0038	5685/5694/5691/5700/5690/5695/5716/5717/5704/64326/5689/5688/5709/5707/5683/5708/5692/10213/7157/10197/5682/5684/5693/9861/5718/5714/5705/7314/5687/5702/5686/6233/5706/5721/1027/5719
174113	SCF-beta-TrCP mediated degradation of Emi1	54	-0,5994	-2,2751	0,0020	0,0038	5685/5694/5691/5700/26271/5690/5695/5716/5717/5704/5689/5688/5709/5707/6500/5683/5708/5692/10213/10197/8454/5682/5684/5693/9861/5718/5714/5705/7314/5687/5702/5686/6233/5706/5721/5719
69052	Switching of origins to a post-replicative state	68	-0,5602	-2,2178	0,0020	0,0038	5685/5694/5691/5700/5690/5695/5716/5717/5704/5689/5925/5688/5709/23595/5707/5683/5708/5692/4175/4176/10213/4999/10197/5682/5684/5693/9861/5718/5714/5705/7314/5687/84515/5001/5702/5686/6233/5706/5721/1027/990/5719
176409	APC/C:Cdc20 mediated degradation of mitotic proteins	69	-0,5327	-2,1045	0,0020	0,0038	5685/29882/5694/7324/5691/5700/51434/5690/5695/5716/5717/5704/5689/5688/8881/5709/8697/5707/5683/246184/5708/5692/10213/891/10197/5682/5684/5693/9861/5718/5714/5705/7314/5687/10393/5702/5686/64682/6233/5706/5721/5719
174178	APC/C:Cdh1 mediated degradation of Cdc20 and other APC/C:Cdh1 targeted proteins in late mitosis/early G1	69	-0,5710	-2,2560	0,0020	0,0038	5685/29882/5694/7324/5691/5700/51434/5690/5695/5716/5717/5704/5689/5688/8881/5709/8697/5707/5683/246184/5708/5692/10213/10197/5682/6502/5684/5693/9861/5718/5714/5705/7314/5687/5347/10393/5702/5686/64682/6233/6790/5706/5721/5719
72764	Eukaryotic Translation Termination	69	-0,5206	-2,0568	0,0020	0,0038	11224/6235/6169/6154/6187/6138/6227/2107/6234/6201/9045/6136/6160/6157/6181/6133/6229/6189/6137/6175/6202/6228/6152/6233/6207/6171/6135/6134/6192
156902	Peptide chain elongation	69	-0,5238	-2,0695	0,0020	0,0038	11224/6235/6169/6154/6187/6138/6227/6234/6201/9045/6136/6160/1915/6157/6181/6133/6229/6189/6137/6175/6202/6228/6152/6233/6207/6171/6135/6134/6192

72203	Processing of Capped Intron-Containing Pre-mRNA	111	-0,6288	-2,7145	0,0020	0,0038	10914/1660/10946/2962/57819/7307/6625/988/2963/11100/5438/9295/9128/3312/10236/57794/11338/10189/3192/9785/6429/4116/5725/10978/8683/84844/1479/4686/53981/56339/83443/23450/5093/11051/51692/3187/5434/56259/9416/65109/3181/6628/3190/9343/51585/6626/1477/5437/6430/6426/10907/27339/6428/22916/6627/5435/5440/4670/5432/4809/6427/5436/51503/10286/6431/24148/6636/6632/6432/3178/6637/6629/6635
3108232	SUMO E3 ligases SUMOylate target proteins	69	-0,4798	-1,8954	0,0020	0,0038	1911/79902/10762/9631/9972/53371/165918/79023/8086/6996/6015/56160/9818/23511/5903/55746/286053/4928/129401/23165/641/8480/23279/493861/81929/23137/7329/7341
3108214	SUMOylation of DNA damage response and repair proteins	69	-0,4798	-1,8954	0,0020	0,0038	1911/79902/10762/9631/9972/53371/165918/79023/8086/6996/6015/56160/9818/23511/5903/55746/286053/4928/129401/23165/641/8480/23279/493861/81929/23137/7329/7341
1236975	Antigen processing-Cross presentation	67	-0,5552	-2,1992	0,0020	0,0038	5685/5694/5691/5700/5690/5695/5716/5717/5704/5689/5688/5709/6892/5707/4688/5683/5708/5692/10213/10197/5682/5684/6891/5693/9861/5718/5714/5705/7314/5687/5702/5686/55176/6233/567/5706/23480/5721/1520/5719/1536
174143	APC/C-mediated degradation of cell cycle proteins	79	-0,5429	-2,2055	0,0020	0,0038	5685/29882/5694/7324/5691/5700/26271/51434/5690/5695/5716/5717/5704/5689/5688/8881/5709/8697/5707/6500/5683/246184/5708/5692/10213/891/10197/8454/5682/6502/5684/5693/9861/5718/5714/5705/7314/5687/5347/10393/5702/5686/64682/6233/6790/5706/5721/5719
179419	APC:Cdc20 mediated degradation of cell cycle proteins prior to satisfation of the cell cycle checkpoint	67	-0,5350	-2,1191	0,0020	0,0038	5685/29882/5694/7324/5691/5700/51434/5690/5695/5716/5717/5704/5689/5688/8881/5709/8697/5707/5683/246184/5708/5692/10213/10197/5682/5684/5693/9861/5718/5714/5705/7314/5687/10393/5702/5686/64682/6233/5706/5721/5719
3371556	Cellular response to heat stress	67	-0,6388	-2,5305	0,0020	0,0038	7531/3312/4927/3281/5594/79902/815/10762/3305/9631/9972/27000/53371/5595/573/79023/8086/51501/23411/9818/9530/23511/5903/7415/55746/4928/129401/2288/7266/23165/8480/23279/81929/3320/3326/10728/9531/10808/3337
69002	DNA Replication Pre-Initiation	79	-0,5231	-2,1252	0,0020	0,0038	5685/5694/5691/5700/6119/5690/5695/5716/5717/5704/5689/5688/5709/23595/5427/5707/5683/5708/5692/4175/4176/10213/51053/4999/5558/10197/5682/5684/10926/5693/9861/5718/5714/5705/7314/5687/84515/5001/5702/5686/6233/8317/5706/5721/990/5719/5426
72706	GTP hydrolysis and joining of the 60S ribosomal subunit	88	-0,5125	-2,1312	0,0020	0,0038	6130/8667/6146/6156/3646/8668/6155/6125/1965/8664/8669/6194/11224/6235/6169/6154/1981/8894/6187/8666/6138/6227/6234/6201/9045/8661/1975/6136/6160/6157/6181/8662/6133/1983/6229/6189/6137/6175/6202/6228/1974/6152/6233/6207/6171/6135/6134/6192
68874	M/G1 Transition	79	-0,5231	-2,1252	0,0020	0,0038	5685/5694/5691/5700/6119/5690/5695/5716/5717/5704/5689/5688/5709/23595/5427/5707/5683/5708/5692/4175/4176/10213/51053/4999/5558/10197/5682/5684/10926/5693/9861/5718/5714/5705/7314/5687/84515/5001/5702/5686/6233/8317/5706/5721/990/5719/5426
453276	Regulation of mitotic cell cycle	79	-0,5429	-2,2055	0,0020	0,0038	5685/29882/5694/7324/5691/5700/26271/51434/5690/5695/5716/5717/5704/5689/5688/8881/5709/8697/5707/6500/5683/246184/5708/5692/10213/891/10197/8454/5682/6502/5684/5693/9861/5718/5714/5705/7314/5687/5347/10393/5702/5686/64682/6233/6790/5706/5721/5719
1799339	SRP-dependent cotranslational protein targeting to membrane	88	-0,5424	-2,2556	0,0020	0,0038	11224/6235/6169/6154/90701/6187/6138/9789/6227/6729/6726/6234/6201/9045/6747/6136/6160/6157/6181/6727/6133/6734/6229/6189/28972/6137/6175/6746/6202/6228/6730/6152/6233/58477/6207/23480/6171/6135/6134/6192
192823	Viral mRNA Translation	67	-0,5221	-2,0680	0,0020	0,0038	11224/6235/6169/6154/6187/6138/6227/6234/6201/9045/6136/6160/6157/6181/6133/6229/6189/6137/6175/6202/6228/6152/6233/6207/6171/6135/6134/6192

69656	Cyclin A:Cdk2-associated events at S phase entry	59	-0,5921	-2,2907	0,0020	0,0038	5685/5694/5691/5700/5690/5695/5716/5717/5704/5689/4331/5925/5688/5709/5707/6500/5683/5708/5692/10213/10197/8454/5682/6502/5684/5693/9861/5718/5714/5705/7314/5687/5702/5686/6233/7465/5706/5721/1027/1022/5719
69202	Cyclin E associated events during G1/S transition	61	-0,5815	-2,2630	0,0020	0,0038	5685/5694/5691/5700/5690/5695/5716/5717/5704/5689/4331/5925/5688/5709/9088/5707/6500/5683/5708/5692/898/10213/10197/8454/5682/6502/5684/5693/9861/5718/5714/5705/7314/5687/5702/5686/6233/7465/5706/5721/1027/1022/5719
5610780	Degradation of GLI1 by the proteasome	59	-0,5662	-2,1906	0,0020	0,0038	5685/5694/5691/5700/8650/5690/5695/5716/5717/5704/5689/5688/5709/5707/6500/5683/5708/5692/10213/2735/10197/8454/5682/5684/5693/9861/9978/5718/5714/5705/7314/5687/5702/5686/6233/5706/5721/5719
5610783	Degradation of GLI2 by the proteasome	59	-0,5625	-2,1763	0,0020	0,0038	5685/5694/5691/5700/5690/5695/5716/5717/5704/1452/5689/5688/5709/5707/6500/5683/5708/5692/10213/10197/8454/5682/5684/5693/9861/9978/5718/5714/5705/7314/5687/5702/5686/6233/5706/5721/5719
5610785	GLI3 is processed to GLI3R by the proteasome	59	-0,5619	-2,1739	0,0020	0,0038	5685/5694/5691/5700/5690/5695/5716/5717/5704/1452/5689/5688/5709/5707/6500/5683/5708/5692/10213/10197/8454/5682/5684/5693/9861/9978/5718/5714/5705/7314/5687/5702/5686/6233/5706/5721/5719
504046	RNA Polymerase I, RNA Polymerase III, and Mitochondrial Transcription	61	-0,6275	-2,4421	0,0020	0,0038	5451/221830/2068/55703/9329/2965/64425/5595/9328/9330/10622/7270/5434/171568/10302/2071/5437/6617/9014/10623/2968/10621/51728/64216/5435/5440/84172/7978/25885/2971/9015/51082/7019/54700/1022/9533/7702/30834
174154	APC/C:Cdc20 mediated degradation of Securin	64	-0,5660	-2,2162	0,0020	0,0038	5685/29882/5694/7324/5691/5700/51434/5690/5695/5716/5717/5704/5689/5688/8881/5709/8697/5707/5683/246184/5708/5692/10213/10197/5682/5684/5693/9861/5718/5714/5705/7314/5687/10393/5702/5686/64682/6233/5706/5721/5719
68867	Assembly of the pre-replicative complex	64	-0,5594	-2,1902	0,0020	0,0038	5685/5694/5691/5700/5690/5695/5716/5717/5704/5689/5688/5709/23595/5707/5683/5708/5692/4175/4176/10213/51053/4999/10197/5682/5684/5693/9861/5718/5714/5705/7314/5687/84515/5001/5702/5686/6233/5706/5721/990/5719
174084	Autodegradation of Cdh1 by Cdh1:APC/C	60	-0,5996	-2,3295	0,0020	0,0038	5685/29882/5694/7324/5691/5700/51434/5690/5695/5716/5717/5704/5689/5688/8881/5709/8697/5707/5683/246184/5708/5692/10213/10197/5682/5684/5693/9861/5718/5714/5705/7314/5687/10393/5702/5686/64682/6233/5706/5721/5719
349425	Autodegradation of the E3 ubiquitin ligase COP1	51	-0,6016	-2,2562	0,0020	0,0038	5685/5694/5691/5700/5690/5695/5716/5717/5704/64326/5689/5688/5709/5707/5683/5708/5692/10213/7157/10197/5682/5684/5693/9861/5718/5714/5705/7314/5687/5702/5686/6233/5706/5721/5719
69206	G1/S Transition	101	-0,4915	-2,1038	0,0020	0,0038	5685/5694/5691/5700/26271/6119/5690/5695/5716/5717/5704/5689/4331/5925/5688/5709/23595/9088/5111/5427/5707/6500/5683/5708/5692/4175/898/4176/10213/4999/891/5558/10197/8454/5682/6502/5684/10926/5693/9861/5718/5714/5705/7314/5687/84515/5001/5702/5686/6233/8317/7465/5706/5721/1027/1022/990/5719/5426
877300	Interferon gamma signaling	60	-0,5126	-1,9916	0,0020	0,0038	3117/85363/3459/3383/6737/5771/55128/3717/3661/3659/4281/3460/7341/3115/567/3113/10346/3664/2634/10379/3394/7412/4502
611105	Respiratory electron transport	64	-0,6039	-2,3645	0,0020	0,0038	7381/7384/6391/4711/9167/4708/2110/4714/1327/54205/55967/1537/4710/6390/4705/4700/4724/1351/4707/126328/2108/4696/7386/4716/4697/4717/7388/4694/27089/1329/4706/1350/4729/1349/4698/4722/4712/6392/4695
167172	Transcription of the HIV genome	60	-0,5935	-2,3059	0,0020	0,0038	8178/904/2962/6917/905/2963/5438/6877/4331/2068/2965/6882/83860/4686/6924/6923/6884/6829/5434/6873/2071/2960/5437/8732/2968/6883/22916/5435/5440/5432/2961/2958/5436/6921/1022/6749

72766	Translation	123	-0,5243	-2,3132	0,0020	0,0038	6130/8667/6146/6156/3646/60559/8668/6155/6125/1965/8664/8669/6194/11224/6235/6169/6154/1981/8894/90701/6187/8666/6138/9789/6227/6729/2107/1937/6726/6234/6201/9045/8661/1975/8892/6747/6136/6160/8891/8890/1915/6157/6181/6727/8662/6133/6734/1983/6229/6189/28972/6137/6175/6746/8893/6202/6228/6730/1974/6152/6233/58477/6207/23480/6171/6135/6134/6192
381119	Unfolded Protein Response (UPR)	64	-0,4694	-1,8381	0,0020	0,0038	10113/1649/7494/9695/7095/811/84447/10954/1639/11015/51360/10130/51726/55738/1965/2673/468/9820/10106/9570/7466/5611/37/7094/9114/3309/116138/3576/9709/6734/30827/55176/27230/1200/6347/4189/58477/23480/64061
180534	Vpu mediated degradation of CD4	51	-0,5755	-2,1582	0,0020	0,0038	5685/5694/5691/5700/5690/5695/5716/5717/5704/5689/5688/5709/5707/6500/5683/5708/5692/10213/10197/5682/5684/5693/9861/5718/5714/5705/7314/5687/5702/5686/6233/5706/5721/5719
983168	Antigen processing: Ubiquitination & Proteasome degradation	53	-0,5802	-2,1937	0,0020	0,0038	5713/5685/5694/5691/5700/5690/5695/5716/5717/5704/5689/5688/5709/5707/5683/5708/5692/10213/10197/5682/5684/5693/9861/5718/5714/5705/7314/5687/5702/5686/6233/5706/5721/5719
1169410	Antiviral mechanism by IFN-stimulated genes	63	-0,5670	-2,2074	0,0020	0,0038	4927/79902/3840/10762/7334/9631/6772/1981/9972/53371/3837/5595/79023/9246/8086/11274/9818/3839/23511/5903/55746/3841/4928/129401/23165/8480/3838/7314/23279/3661/81929/389898/5300/1974/6233/5335/9775
450408	AUF1 (hnRNP D0) destabilizes mRNA	53	-0,6100	-2,3066	0,0020	0,0038	5685/5694/5691/5700/5690/5695/5716/5717/5704/5689/3312/5688/1981/5709/5707/5683/5708/5692/10213/10197/5682/5684/5693/9861/3315/5718/5714/5705/7314/5687/5702/5686/6233/5706/5721/5719
983169	Class I MHC mediated antigen processing & presentation	82	-0,5099	-2,0807	0,0020	0,0038	5685/5694/5691/5700/5690/5695/5716/5717/5704/10134/5689/5688/5709/9632/6892/5707/4688/5683/5708/6396/5692/10213/10197/64167/5682/5684/6891/5693/9861/5718/5714/5705/7314/5687/10802/5702/5686/55176/6233/567/5706/23480/5721/1520/5719/1536
1834949	Cytosolic sensors of pathogen-associated DNA	56	-0,5910	-2,2542	0,0020	0,0038	10622/4790/22954/6778/8737/51428/5434/171568/2547/5437/115004/5970/6737/10623/3551/10621/7314/51728/5435/5440/3661/1147/4361/6233/9208/51082/3428/9533/4792
1169408	ISG15 antiviral mechanism	63	-0,5670	-2,2074	0,0020	0,0038	4927/79902/3840/10762/7334/9631/6772/1981/9972/53371/3837/5595/79023/9246/8086/11274/9818/3839/23511/5903/55746/3841/4928/129401/23165/8480/3838/7314/23279/3661/81929/389898/5300/1974/6233/5335/9775
211733	Regulation of activated PAK-2p34 by proteasome mediated degradation	49	-0,5984	-2,2288	0,0020	0,0038	5685/5694/5691/5700/5690/5695/5716/5717/5704/5689/5688/5709/5707/5683/5708/5692/10213/10197/5682/5684/5693/9861/5718/5714/5705/7314/5687/5702/5686/6233/5706/5721/5719
3371453	Regulation of HSF1-mediated heat shock response	49	-0,6623	-2,4670	0,0020	0,0038	7531/3312/4927/5594/79902/10762/9631/9972/27000/53371/5595/573/79023/8086/51501/23411/9818/9530/23511/5903/55746/4928/129401/7266/23165/8480/23279/81929/9531/10808/3337
163200	Respiratory electron transport, ATP synthesis by chemiosmotic coupling, and heat production by uncoupling proteins.	83	-0,5592	-2,2921	0,0020	0,0038	4702/9377/7381/7384/6391/4711/9167/4708/2110/4714/1327/54205/518/55967/1537/4710/6390/4705/498/4700/4724/1351/4707/126328/515/2108/506/4696/7386/4716/4697/4717/7388/4694/27089/1329/4706/1350/10632/4729/1349/4698/4722/4712/6392/4695
180585	Vif-mediated degradation of APOBEC3G	53	-0,6020	-2,2762	0,0020	0,0038	5685/5694/5691/5700/5690/5695/5716/5717/5704/5689/5688/5709/5707/6923/5683/5708/5692/10213/10197/5682/5684/5693/9861/9978/5718/5714/5705/7314/5687/5702/5686/6921/6233/5706/5721/5719
69017	CDK-mediated phosphorylation and removal of Cdc6	50	-0,6055	-2,2671	0,0020	0,0038	5685/5694/5691/5700/5690/5695/5716/5717/5704/5689/5688/5709/5707/5683/5708/5692/10213/10197/5682/5684/5693/9861/5718/5714/5705/7314/5687/5702/5686/6233/5706/5721/990/5719
5358346	Hedgehog ligand biogenesis	62	-0,5231	-2,0390	0,0020	0,0038	5713/5685/5694/5691/5700/5690/5695/10956/85455/5716/5717/5704/5689/5688/5709/5707/51009/5683/5708/5692/10213/10197/7415/5682/5684/5693/9861/5718/5714/5705/7314/5687/5702/5034/5686/2262/6233/5706/5721/5719

5389840	Mitochondrial translation elongation	81	-0,5721	-2,3256	0,0020	0,0038	84545/128308/51073/3396/60488/63931/51021/65993/116540/51258/23107/51253/51069/57129/64965/65005/29088/28973/64976/51650/740/64960/118487/10884/54534/9553/56945/124995/28998/54148/219927/55173/10240/64949/55037/7284/10102/92259/55052/65080/85476/51318/65008/29093/51023/26589/29074/6183/51649/28957
5368286	Mitochondrial translation initiation	81	-0,5567	-2,2630	0,0020	0,0038	84545/128308/51073/3396/60488/63931/51021/65993/116540/51258/23107/51253/51069/57129/64965/65005/29088/28973/64976/51650/740/64960/118487/10884/54534/9553/123263/56945/124995/28998/54148/219927/55173/10240/64949/55037/92259/55052/65080/51318/65008/29093/51023/26589/29074/6183/51649/28957
5419276	Mitochondrial translation termination	81	-0,5466	-2,2222	0,0020	0,0038	84545/128308/51073/3396/60488/63931/51021/65993/116540/51258/23107/51253/51069/57129/64965/65005/29088/28973/64976/51650/740/64960/118487/10884/54534/9553/56945/124995/28998/54148/219927/55173/10240/64949/55037/92259/55052/65080/51318/65008/29093/51023/26589/29074/6183/51649/28957
69610	p53-Independent DNA Damage Response	52	-0,5917	-2,2246	0,0020	0,0038	5685/5694/5691/5700/1111/5690/5695/5716/5717/5704/5689/5688/5709/5707/5683/5708/5692/10213/10197/5682/5684/5693/9861/5718/5714/5705/7314/5687/5702/5686/6233/5706/5721/5719
69613	p53-Independent G1/S DNA damage checkpoint	52	-0,5917	-2,2246	0,0020	0,0038	5685/5694/5691/5700/1111/5690/5695/5716/5717/5704/5689/5688/5709/5707/5683/5708/5692/10213/10197/5682/5684/5693/9861/5718/5714/5705/7314/5687/5702/5686/6233/5706/5721/5719
169911	Regulation of Apoptosis	50	-0,6029	-2,2575	0,0020	0,0038	5685/5694/5691/5700/5690/5695/5716/5717/5704/5689/5688/5709/5707/5683/5708/5692/10213/10197/5682/5684/5693/9861/5718/5714/5705/7314/5687/5702/5686/79658/6233/5706/5721/5719
350562	Regulation of ornithine decarboxylase (ODC)	50	-0,5749	-2,1526	0,0020	0,0038	5685/5694/5691/5700/4946/5690/5695/5716/5717/5704/5689/5688/4947/5709/5707/5683/5708/5692/10213/10197/5682/5684/5693/51582/9861/5718/1728/5714/5705/5687/5702/5686/5706/5721/5719
187577	SCF(Skp2)-mediated degradation of p27/p21	52	-0,5858	-2,2025	0,0020	0,0038	5685/5694/5691/5700/5690/5695/5716/5717/5704/5689/5688/5709/5707/6500/5683/5708/5692/10213/10197/8454/5682/6502/5684/5693/9861/5718/5714/5705/7314/5687/5702/5686/6233/5706/5721/1027/5719
69541	Stabilization of p53	52	-0,5965	-2,2426	0,0020	0,0038	5685/5694/5691/5700/5690/5695/5716/5717/5704/64326/5689/5688/5709/5707/5683/5708/5692/10213/7157/10197/5682/5684/5693/9861/5718/5714/5705/7314/5687/5702/5686/6233/5706/5721/5719
69601	Ubiquitin Mediated Degradation of Phosphorylated Cdc25A	52	-0,5917	-2,2246	0,0020	0,0038	5685/5694/5691/5700/1111/5690/5695/5716/5717/5704/5689/5688/5709/5707/5683/5708/5692/10213/10197/5682/5684/5693/9861/5718/5714/5705/7314/5687/5702/5686/6233/5706/5721/5719
75815	Ubiquitin-dependent degradation of Cyclin D	50	-0,6062	-2,2699	0,0020	0,0038	5685/5694/5691/5700/5690/5695/5716/5717/5704/5689/5688/5709/5707/5683/5708/5692/10213/10197/5682/5684/5693/9861/5718/5714/5705/7314/5687/5702/5686/6233/5706/5721/1019/5719
69229	Ubiquitin-dependent degradation of Cyclin D1	50	-0,6062	-2,2699	0,0020	0,0038	5685/5694/5691/5700/5690/5695/5716/5717/5704/5689/5688/5709/5707/5683/5708/5692/10213/10197/5682/5684/5693/9861/5718/5714/5705/7314/5687/5702/5686/6233/5706/5721/1019/5719
1169091	Activation of NF-kappaB in B cells	66	-0,5584	-2,1987	0,0020	0,0038	5685/5694/5691/5700/5690/5695/5716/5717/5704/5689/5688/10892/6885/5709/23291/5707/6500/4790/5683/5708/5692/10213/10197/8454/5682/5966/5970/5684/5693/3551/9861/5718/5714/5705/7314/4794/5687/1147/5702/5686/6233/5706/5721/5719/4792

174184	Cdc20:Phospho-APC/C mediated degradation of Cyclin A	66	-0,5515	-2,1715	0,0020	0,0038	5685/29882/5694/7324/5691/5700/51434/5690/5695/5716/5717/5704/5689/5688/8881/5709/8697/5707/5683/246184/5708/5692/10213/10197/5682/5684/5693/9861/5718/5714/5705/7314/5687/10393/5702/5686/64682/6233/5706/5721/5719
195253	Degradation of beta-catenin by the destruction complex	66	-0,5731	-2,2567	0,0020	0,0038	5685/5694/5691/5700/5690/5695/5716/5717/5704/1452/5689/5688/5709/5707/6500/5683/6934/5708/5692/10213/10197/8454/5682/5684/5693/9861/9978/5718/5714/5705/7314/5687/5702/5686/6233/5706/5721/5719
73885	Nucleotide Excision Repair	47	-0,5721	-2,1087	0,0020	0,0038	4331/2068/5985/1161/2965/5111/5427/2072/1642/56949/5434/2071/5437/2968/10714/5435/5440/5432/5436/5982/328/5984/1022/5426
73857	RNA Polymerase II Transcription	99	-0,6195	-2,6348	0,0020	0,0038	10914/134353/2962/6917/7307/905/2963/5438/9295/6877/4331/2068/11338/10189/9785/6429/4116/2965/6882/10978/8683/83860/1479/4686/6924/53981/6923/11051/51692/6884/6829/5434/65109/6873/6628/2071/51585/2960/1477/5437/6430/6426/8732/2968/6883/6428/22916/5435/5440/5432/6427/2961/2958/5436/6921/6431/25888/6636/6432/1022/6749/6637/6635
1236978	Cross-presentation of soluble exogenous antigens (endosomes)	45	-0,5906	-2,1550	0,0020	0,0038	5685/5694/5691/5700/5690/5695/5716/5717/5704/5689/5688/5709/5707/5683/5708/5692/10213/10197/5682/5684/5693/9861/5718/5714/5705/5687/5702/5686/5706/5721/5719
5621481	C-type lectin receptors (CLRs)	117	-0,4600	-2,0092	0,0020	0,0038	808/801/5713/5685/5694/5691/5063/5700/5532/3709/5530/4775/5690/5336/5695/5170/5716/5717/5704/7322/5689/5534/5688/23118/10892/7334/6885/5709/23291/5707/5894/6500/4790/5683/5708/5692/10213/805/10197/4773/8454/5682/5970/5684/5693/3551/9861/5718/5714/5705/7314/2534/5687/1147/9252/389898/5702/5686/50856/6233/5706/5721/5719/2207/4792
162587	HIV Life Cycle	124	-0,4970	-2,1891	0,0020	0,0038	5045/8178/27183/27243/904/7520/2962/6917/905/2963/5438/5902/6877/137492/4927/4331/79902/2068/10762/9631/9972/53371/2965/23327/6882/79023/8086/83860/4686/6924/6923/6884/9818/23511/6829/5434/2547/5903/6873/1234/55746/2071/2960/4836/5437/8732/4928/2968/7251/129401/23165/6883/51510/22916/8480/7314/23279/5435/5440/5901/5432/81929/2961/2958/5436/6921/6233/1022/6749
162909	Host Interactions of HIV factors	122	-0,4917	-2,1655	0,0020	0,0038	5713/5685/5694/292/5691/162/5700/5690/904/1794/5695/8907/161/5716/5717/5704/3836/5902/5689/4927/5688/79902/10762/9631/9972/5709/53371/3837/51606/164/5707/79023/8086/6500/6923/5683/5708/5692/9818/10213/23511/5903/10197/55746/160/5682/5684/4928/129401/4869/5693/23165/9861/9978/8480/5718/5714/5705/7314/2534/23279/5901/5687/81929/291/5702/5686/5879/6921/6233/567/5706/5721/5719
453279	Mitotic G1-G1/S phases	124	-0,4243	-1,8688	0,0020	0,0038	5685/5694/5691/1859/5700/26271/6119/5690/5695/5716/5717/5704/5689/4331/5925/5688/5933/286826/5928/5709/23595/9088/5111/5427/5707/6500/5683/5708/5692/4175/898/4176/10213/4999/891/5558/10197/132660/8454/5682/6502/5684/10926/5693/9861/5718/5714/5705/7314/5687/84515/5001/5702/5686/55957/6233/8317/7465/5706/5721/1027/1022/990/1019/5719/5426
69242	S Phase	117	-0,4965	-2,1690	0,0020	0,0038	5685/5694/9837/5691/5700/6119/5690/5695/5716/5717/5704/2237/114799/5689/4331/5925/5688/5985/5709/1763/23595/5111/5427/5707/6500/5683/5708/5692/4175/4176/10213/4999/5558/10197/8454/5682/6502/5684/5693/9861/5718/5714/5705/7314/10714/5687/84515/5001/5702/5686/5982/328/6233/5984/7465/5706/5721/1027/1022/990/1019/5719/5426
4608870	Asymmetric localization of PCP proteins	58	-0,5619	-2,1511	0,0020	0,0038	5685/5694/5691/5700/5690/5695/64750/5716/5717/5704/5689/5688/5709/5707/5683/5708/5692/10213/1856/10197/5682/5684/5693/9861/5718/5714/5705/7314/5687/5702/5686/6233/7474/5706/5721/5719

68827	CDT1 association with the CDC6:ORC:origin complex	58	-0,5737	-2,1964	0,0020	0,0038	5685/5694/5691/5700/5690/5695/5716/5717/5704/5689/5688/5709/23595/5707/5683/5708/5692/10213/51053/4999/10197/5682/5684/5693/9861/5718/5714/5705/7314/5687/84515/5001/5702/5686/6233/5706/5721/990/5719
69620	Cell Cycle Checkpoints	120	-0,4930	-2,1552	0,0020	0,0038	5685/29882/7532/5694/7324/5691/5700/6119/51434/1111/5690/5695/5716/5717/5704/7531/64326/5689/5688/8881/5985/5709/23595/8697/9088/5707/5683/246184/5708/5692/4175/4176/10213/4999/891/7157/10197/5682/5684/10926/5693/9861/5718/5714/5705/7314/5687/84515/5001/10393/5702/5686/64682/5982/6233/8317/5984/7465/5706/5721/1027/990/5719/9133
5607761	Dectin-1 mediated noncanonical NF-kB signaling	58	-0,5572	-2,1330	0,0020	0,0038	5685/5694/5691/5700/5690/5695/5716/5717/5704/5689/5688/5709/23291/5707/6500/5683/5708/5692/10213/10197/8454/5682/5684/5693/9861/5718/5714/5705/7314/5687/1147/5702/5686/6233/5706/5721/5719
69306	DNA Replication	100	-0,5209	-2,2178	0,0020	0,0038	5685/5694/9837/5691/5700/6119/5690/5695/5716/5717/5704/2237/5689/5925/5688/5985/5709/1763/23595/5111/5427/5707/5683/5708/5692/4175/4176/10213/51053/4999/5558/10197/5682/5684/10926/5693/9861/5718/5714/5705/7314/10714/5687/84515/5001/5702/5686/5982/328/6233/8317/5984/5706/5721/1027/990/5719/5426
69615	G1/S DNA Damage Checkpoints	58	-0,5920	-2,2664	0,0020	0,0038	5685/5694/5691/5700/1111/5690/5695/5716/5717/5704/64326/5689/5688/5709/5707/5683/5708/5692/10213/7157/10197/5682/5684/5693/9861/5718/5714/5705/7314/5687/5702/5686/6233/5706/5721/1027/5719
168254	Influenza Infection	100	-0,5483	-2,3343	0,0020	0,0038	6194/11224/6235/79902/10762/6169/6154/9631/9972/53371/3837/10898/79023/6187/8086/6138/6227/9818/23511/6234/6201/9045/5903/6136/55746/6160/6157/6181/4928/129401/23165/8480/6133/23279/5901/6229/6189/81929/6137/6175/6202/6228/6152/6233/6207/6171/6135/6134/6192
375276	Peptide ligand-binding receptors	184	0,4020	1,9207	0,0020	0,0038	2861/623/624/10663/4988/2834/6368/2852/10874/5368/3827/2925/5367/6375/4159/7200/729230/128674/283869/6348/6360/51289/84539/6870/51554/5539/1235/551/6361/2862/256933/84109/8811/23620/6750/9034/2829/1908/552/84634/6366/3061/347148/1232/6376/51083/59350/4829/2149/10316/6753/2832/4985/200558/1238/5473/2358/27202/4887/4922/10022/887/5697/1230/4158/885/2922/8484/5173/129521/4923/84432/117579/56923/3060/56477/64106/2837/553/4852/2587/2831/6352/6755/6354/51052/50865/6387/2826/6374/4160/3640/5179/10803/9002/2833/886/1237/2147/3062/1524/7852/10886/6372/122042/1907/2151/4987/5020
674695	RNA Polymerase II Pre-transcription Events	58	-0,5956	-2,2802	0,0020	0,0038	8178/904/2962/6917/905/2963/5438/6877/4331/2068/2965/6882/83860/4686/6924/6923/6884/6829/5434/6873/2071/2960/5437/2968/6883/22916/5435/5440/5432/2961/2958/5436/6921/1022/6749
1428517	The citric acid (TCA) cycle and respiratory electron transport	120	-0,5448	-2,3817	0,0020	0,0038	6391/51660/4711/23530/5163/9167/4708/2110/1738/4714/1327/54205/518/55967/1537/682/4710/6390/3420/3939/4705/498/4700/4724/1351/8803/4707/126328/515/2271/3945/2108/506/4696/7386/1737/5166/4716/4697/4717/7388/4694/27089/1329/8050/5160/5162/4706/1350/10632/4729/1349/4698/4722/4712/5164/6392/4695/8801
418594	G alpha (i) signalling events	191	0,3949	1,8936	0,0021	0,0038	259289/1902/623/624/4543/10663/4988/6368/1268/2852/10874/150/5368/3827/53637/5367/9170/3354/151/729230/283869/6360/50838/51289/84539/1129/2771/5539/1235/256933/353164/53829/8811/23566/846/3351/6750/2913/165140/6366/9294/111/59340/1232/3352/5733/51083/2918/4544/10316/56670/6753/2832/50833/4985/5473/338442/2780/2358/1813/4887/259286/10022/5697/1230/8698/8484/6010/27199/5173/129521/117579/56923/114/2840/8843/1132/56477/259285/4852/2587/2916/3350/2831/6352/2770/57121/6755

1236974	ER-Phagosome pathway	57	-0,5923	-2,2571	0,0021	0,0038	5685/5694/5691/5700/5690/5695/5716/5717/5704/5689/5688/5709/6892/5707/5683/5708/5692/10213/10197/5682/5684/6891/5693/9861/5718/5714/5705/7314/5687/5702/5686/55176/6233/567/5706/23480/5721/5719
416476	G alpha (q) signalling events	159	0,4158	1,9439	0,0021	0,0038	1902/623/624/7225/23236/6368/10874/3827/2925/9630/5588/1607/5367/6375/9170/7200/128674/84539/6870/1128/551/2862/27334/2864/84109/23566/23620/846/2829/1908/552/84634/2867/3710/3061/347148/29933/4829/2149/10316/3269/3358/222545/2641/200558/2642/2358/4922/887/5724/2798/885/2922/129521/4923/84432/94233/7222/56923/2840/3060/8111/64106/2837/2797/553/8997/156
881907	Gastrin-CREB signalling pathway via PKC and MAPK	179	0,3852	1,8288	0,0021	0,0038	1902/623/624/7225/23236/6368/10874/3827/2925/9630/5588/1607/5367/6375/9170/7200/128674/84539/6870/1128/551/2862/27334/2864/84109/23566/23620/6654/846/2829/1908/552/6196/84634/2867/3710/3061/347148/29933/4829/2149/10316/3269/3358/222545/2641/200558/2642/2358/4922/5605/887/5724/2798/885/2922/129521/4923/84432/94233/983/7222/56923/2840/3060/8111/64106/2837/2797/553/8997/156
167152	Formation of HIV elongation complex in the absence of HIV Tat	39	-0,6357	-2,2299	0,0021	0,0038	904/2962/6917/905/2963/5438/4331/2068/2965/4686/6924/6923/6829/5434/2071/5437/2968/22916/5435/5440/5432/5436/6921/1022/6749
112382	Formation of RNA Pol II elongation complex	39	-0,6357	-2,2299	0,0021	0,0038	904/2962/6917/905/2963/5438/4331/2068/2965/4686/6924/6923/6829/5434/2071/5437/2968/22916/5435/5440/5432/5436/6921/1022/6749
2980766	Nuclear Envelope Breakdown	40	-0,5999	-2,1079	0,0021	0,0038	79902/10762/9631/9972/53371/79023/91754/8086/9818/23511/891/23175/5903/55746/4928/129401/23165/5578/8480/23279/81929/5347/2010/10783/255919/7443/9133
75955	RNA Polymerase II Transcription Elongation	39	-0,6357	-2,2299	0,0021	0,0038	904/2962/6917/905/2963/5438/4331/2068/2965/4686/6924/6923/6829/5434/2071/5437/2968/22916/5435/5440/5432/5436/6921/1022/6749
2173782	Binding and Uptake of Ligands by Scavenger Receptors	37	-0,5869	-2,0280	0,0021	0,0038	2495/3320/3250/81035/3240/1278/6678/348/10808/9332/3043
167200	Formation of HIV-1 elongation complex containing HIV-1 Tat	38	-0,6337	-2,2031	0,0021	0,0038	904/2962/6917/2963/5438/4331/2068/2965/4686/6924/6923/6829/5434/2071/5437/2968/22916/5435/5440/5432/5436/6921/1022/6749
167169	HIV Transcription Elongation	38	-0,6337	-2,2031	0,0021	0,0038	904/2962/6917/2963/5438/4331/2068/2965/4686/6924/6923/6829/5434/2071/5437/2968/22916/5435/5440/5432/5436/6921/1022/6749
72165	mRNA Splicing - Minor Pathway	42	-0,6670	-2,3653	0,0021	0,0038	83443/23450/5434/9416/6628/9343/5437/6426/10907/22916/5435/5440/5432/4809/6427/5436/6431/24148/6636/6632/6432/6637/6635
167246	Tat-mediated elongation of the HIV-1 transcript	38	-0,6337	-2,2031	0,0021	0,0038	904/2962/6917/2963/5438/4331/2068/2965/4686/6924/6923/6829/5434/2071/5437/2968/22916/5435/5440/5432/5436/6921/1022/6749
73937	Transcription-coupled NER (TC-NER)	42	-0,5989	-2,1236	0,0021	0,0038	4331/2068/5985/1161/2965/5111/5427/2072/56949/5434/2071/5437/2968/10714/5435/5440/5432/5436/5982/328/5984/1022/5426
109688	Cleavage of Growing Transcript in the Termination Region	41	-0,6640	-2,3443	0,0022	0,0038	134353/7307/9295/11338/10189/9785/6429/4116/10978/8683/1479/4686/53981/11051/51692/65109/6628/51585/1477/6430/6426/6428/22916/6427/6431/25888/6636/6432/6637/6635
110304	Dual incision reaction in TC-NER	25	-0,6692	-2,1361	0,0022	0,0038	5438/4331/2068/1161/2965/2072/56949/5434/2071/5437/2968/5435/5440/5432/5436/1022
110302	Formation of transcription-coupled NER (TC-NER) repair complex	25	-0,6692	-2,1361	0,0022	0,0038	5438/4331/2068/1161/2965/2072/56949/5434/2071/5437/2968/5435/5440/5432/5436/1022
167161	HIV Transcription Initiation	41	-0,5959	-2,1037	0,0022	0,0038	5438/6877/4331/2068/2965/6882/83860/6884/5434/6873/2071/2960/5437/2968/6883/5435/5440/5432/2961/2958/5436/1022
76044	Post-Elongation Processing of the Transcript	41	-0,6640	-2,3443	0,0022	0,0038	134353/7307/9295/11338/10189/9785/6429/4116/10978/8683/1479/4686/53981/11051/51692/65109/6628/51585/1477/6430/6426/6428/22916/6427/6431/25888/6636/6432/6637/6635
389957	Prefoldin mediated transfer of substrate to CCT/TriC	25	-0,5943	-1,8968	0,0022	0,0038	81027/7277/84790/6950/5204/7203/10574/10576/10471/7411/10694/908/22948/10383/10575/5203/347733

71406	Pyruvate metabolism and Citric Acid (TCA) cycle	41	-0,5444	-1,9219	0,0022	0,0038	6391/51660/23530/5163/1738/682/6390/3420/3939/8803/2271/3945/1737/5166/8050/5160/5162/5164/6392/8801
77075	RNA Pol II CTD phosphorylation and interaction with CE	25	-0,6555	-2,0923	0,0022	0,0038	2963/5438/4331/2068/2965/6829/5434/2071/5437/8732/2968/5435/5440/5432/5436/1022
167160	RNA Pol II CTD phosphorylation and interaction with CE	25	-0,6555	-2,0923	0,0022	0,0038	2963/5438/4331/2068/2965/6829/5434/2071/5437/8732/2968/5435/5440/5432/5436/1022
167162	RNA Polymerase II HIV Promoter Escape	41	-0,5959	-2,1037	0,0022	0,0038	5438/6877/4331/2068/2965/6882/83860/6884/5434/6873/2071/2960/5437/2968/6883/5435/5440/5432/2961/2958/5436/1022
73776	RNA Polymerase II Promoter Escape	41	-0,5959	-2,1037	0,0022	0,0038	5438/6877/4331/2068/2965/6882/83860/6884/5434/6873/2071/2960/5437/2968/6883/5435/5440/5432/2961/2958/5436/1022
75953	RNA Polymerase II Transcription Initiation	41	-0,5959	-2,1037	0,0022	0,0038	5438/6877/4331/2068/2965/6882/83860/6884/5434/6873/2071/2960/5437/2968/6883/5435/5440/5432/2961/2958/5436/1022
76042	RNA Polymerase II Transcription Initiation And Promoter Clearance	41	-0,5959	-2,1037	0,0022	0,0038	5438/6877/4331/2068/2965/6882/83860/6884/5434/6873/2071/2960/5437/2968/6883/5435/5440/5432/2961/2958/5436/1022
73779	RNA Polymerase II Transcription Pre-Initiation And Promoter Opening	41	-0,5959	-2,1037	0,0022	0,0038	5438/6877/4331/2068/2965/6882/83860/6884/5434/6873/2071/2960/5437/2968/6883/5435/5440/5432/2961/2958/5436/1022
73856	RNA Polymerase II Transcription Termination	41	-0,6640	-2,3443	0,0022	0,0038	134353/7307/9295/11338/10189/9785/6429/4116/10978/8683/1479/4686/53981/11051/51692/65109/6628/51585/1477/6430/6426/6428/22916/6427/6431/25888/6636/6432/6637/6635
76066	RNA Polymerase III Transcription Initiation From Type 2 Promoter	25	-0,6592	-2,1040	0,0022	0,0038	2976/55814/6908/661/27297/84265/55703/9329/9328/9330/10622/5434/171568/5437/10623/10621/51728/5435/5440/51082/9533
379724	tRNA Aminoacylation	41	-0,5774	-2,0386	0,0022	0,0038	5464/3376/833/57038/80222/4141/3735/3035/5859/51520/6897/23438/10667/57176/51067/8565/10056/7453/5917/6301/7965/7407/10352/1615/57505/79731
168325	Viral Messenger RNA Synthesis	25	-0,6895	-2,2007	0,0022	0,0038	79902/10762/9631/9972/53371/79023/8086/9818/23511/5903/55746/4928/129401/23165/8480/23279/81929
1268020	Mitochondrial protein import	28	-0,6371	-2,0828	0,0022	0,0038	25813/9512/9868/131474/26521/131118/3313/29928/10651/26520/9804/56993/29090/23203/80273/10440/26519
180746	Nuclear import of Rev protein	28	-0,7028	-2,2974	0,0022	0,0038	79902/10762/9631/9972/53371/3837/79023/8086/9818/23511/5903/55746/4928/129401/4869/23165/8480/23279/5901/81929
167238	Pausing and recovery of Tat-mediated HIV elongation	28	-0,6212	-2,0307	0,0022	0,0038	904/2962/6917/2963/5438/6924/6923/6829/5434/5437/5435/5440/5432/5436/6921/6749
165054	Rev-mediated nuclear export of HIV RNA	28	-0,6826	-2,2314	0,0022	0,0038	4927/79902/10762/9631/9972/53371/79023/8086/9818/23511/5903/55746/4928/129401/23165/8480/23279/5901/81929
73762	RNA Polymerase I Transcription Initiation	28	-0,7019	-2,2944	0,0022	0,0038	221830/2068/2965/64425/5434/2071/5437/9014/2968/5435/5440/84172/25885/9015/51082/54700/1022/9533/30834
73863	RNA Polymerase I Transcription Termination	28	-0,6772	-2,2136	0,0022	0,0038	221830/2068/2965/64425/7270/5434/2071/5437/9014/2968/5435/5440/84172/25885/9015/51082/1022/9533/30834
167243	Tat-mediated HIV elongation arrest and recovery	28	-0,6212	-2,0307	0,0022	0,0038	904/2962/6917/2963/5438/6924/6923/6829/5434/5437/5435/5440/5432/5436/6921/6749
112387	Elongation arrest and recovery	29	-0,6243	-2,0385	0,0022	0,0038	904/2962/6917/905/2963/5438/6924/6923/6829/5434/5437/5435/5440/5432/5436/6921/6749
113418	Formation of the Early Elongation Complex	29	-0,6397	-2,0887	0,0022	0,0038	2963/5438/4331/2068/2965/4686/6829/5434/2071/5437/2968/22916/5435/5440/5432/5436/1022
167158	Formation of the HIV-1 Early Elongation Complex	29	-0,6397	-2,0887	0,0022	0,0038	2963/5438/4331/2068/2965/4686/6829/5434/2071/5437/2968/22916/5435/5440/5432/5436/1022
167287	HIV elongation arrest and recovery	29	-0,6243	-2,0385	0,0022	0,0038	904/2962/6917/905/2963/5438/6924/6923/6829/5434/5437/5435/5440/5432/5436/6921/6749
167290	Pausing and recovery of HIV elongation	29	-0,6243	-2,0385	0,0022	0,0038	904/2962/6917/905/2963/5438/6924/6923/6829/5434/5437/5435/5440/5432/5436/6921/6749

73854	RNA Polymerase I Promoter Clearance	29	-0,7040	-2,2987	0,0022	0,0038	221830/2068/2965/64425/5595/5434/2071/5437/9014/2968/5435/5440/84172/25885/9015/51082/54700/1022/9533/30834
166658	Complement cascade	34	-0,5751	-1,9454	0,0022	0,0038	715/966/714/720/716/730/3075
391251	Protein folding	31	-0,5761	-1,9029	0,0022	0,0038	81027/7277/84790/6950/6903/5204/7203/6905/10574/10576/10471/7411/10694/908/22948/6904/10383/10575/5203/347733
73864	RNA Polymerase I Transcription	31	-0,6728	-2,2222	0,0022	0,0038	221830/2068/2965/64425/5595/7270/5434/2071/5437/9014/2968/5435/5440/84172/25885/9015/51082/54700/1022/9533/30834
749476	RNA Polymerase III Abortive And Retractive Initiation	34	-0,6486	-2,1937	0,0022	0,0038	11128/6619/2976/55814/6618/6908/661/27297/84265/5451/55703/9329/9328/9330/10622/5434/171568/10302/5437/6617/10623/10621/51728/5435/5440/2971/51082/9533/7702
74158	RNA Polymerase III Transcription	34	-0,6486	-2,1937	0,0022	0,0038	11128/6619/2976/55814/6618/6908/661/27297/84265/5451/55703/9329/9328/9330/10622/5434/171568/10302/5437/6617/10623/10621/51728/5435/5440/2971/51082/9533/7702
76046	RNA Polymerase III Transcription Initiation	34	-0,6486	-2,1937	0,0022	0,0038	11128/6619/2976/55814/6618/6908/661/27297/84265/5451/55703/9329/9328/9330/10622/5434/171568/10302/5437/6617/10623/10621/51728/5435/5440/2971/51082/9533/7702
73777	RNA Polymerase I Chain Elongation	26	-0,7124	-2,2799	0,0022	0,0038	221830/2068/2965/64425/5434/2071/5437/9014/2968/5435/5440/84172/25885/9015/51082/1022/9533/30834
76061	RNA Polymerase III Transcription Initiation From Type 1 Promoter	26	-0,6604	-2,1134	0,0022	0,0038	2976/55814/6908/661/27297/84265/55703/9329/9328/9330/10622/5434/171568/5437/10623/10621/51728/5435/5440/2971/51082/9533
177243	Interactions of Rev with host cellular proteins	30	-0,6905	-2,2688	0,0022	0,0038	4927/79902/10762/9631/9972/53371/3837/79023/8086/9818/23511/5903/55746/4928/129401/4869/23165/8480/23279/5901/81929
3301854	Nuclear Pore Complex (NPC) Disassembly	30	-0,6840	-2,2474	0,0022	0,0038	79902/10762/9631/9972/53371/79023/91754/8086/9818/23511/891/5903/55746/4928/129401/23165/8480/23279/81929/10783/9133
109970	Global Genomic NER (GG-NER)	32	-0,5515	-1,8435	0,0022	0,0038	2068/5985/2965/5111/5427/2072/1642/2071/2968/10714/5982/328/5984/1022/5426
72187	mRNA 3'-end processing	32	-0,6696	-2,2384	0,0022	0,0038	7307/9295/11338/10189/9785/6429/4116/10978/8683/1479/4686/53981/11051/51692/65109/51585/1477/6430/6426/6428/22916/6427/6431/6432
112296	Post-Elongation Processing of Intron-Containing pre-mRNA	32	-0,6696	-2,2384	0,0022	0,0038	7307/9295/11338/10189/9785/6429/4116/10978/8683/1479/4686/53981/11051/51692/65109/51585/1477/6430/6426/6428/22916/6427/6431/6432
72086	mRNA Capping	27	-0,6644	-2,1442	0,0022	0,0038	2963/5438/4331/2068/2965/4686/6829/5434/2071/5437/8732/2968/22916/5435/5440/5432/5436/1022
170822	Regulation of Glucokinase by Glucokinase Regulatory Protein	27	-0,6416	-2,0706	0,0022	0,0038	79902/10762/9631/9972/53371/79023/8086/9818/23511/5903/55746/4928/129401/23165/8480/23279/81929
73772	RNA Polymerase I Promoter Escape	27	-0,7171	-2,3142	0,0022	0,0038	221830/2068/2965/64425/5434/2071/5437/9014/2968/5435/5440/84172/25885/9015/51082/54700/1022/9533/30834
76071	RNA Polymerase III Transcription Initiation From Type 3 Promoter	27	-0,6534	-2,1086	0,0022	0,0038	11128/6619/55814/6618/6908/661/27297/84265/5451/55703/10622/5434/171568/10302/5437/6617/10623/10621/51728/5435/5440/51082/9533/7702
72202	Transport of Mature Transcript to Cytoplasm	27	-0,6540	-2,1107	0,0022	0,0038	9295/11338/10189/9785/6429/4116/8683/4686/53981/51692/65109/6430/6426/6428/22916/6427/6431/6432
5617833	Assembly of the primary cilium	157	-0,3598	-1,6463	0,0038	0,0064	11020/5048/347240/79600/11336/1453/200894/123016/64792/7532/11127/10121/1639/132320/80184/5116/261734/84100/8481/26005/8100/7283/23332/117177/9742/7277/7531/50807/4957/10640/149371/79809/403/583/57728/51626/51668/54585/11258/9657/55112/22832/90410/80321/55142/11116/79867/54536/80254/5518/79959/8766/79659/55212/9662/51259/8655/11064/129880/22995/55081/5310/51098/91147/84902/5347/79989/3320/57539/378/10383/23059/80173/84747/26123

68882	Mitotic Anaphase	155	-0,3660	-1,6708	0,0039	0,0064	5685/29882/5694/7324/5691/5700/51434/5690/5695/5716/5717/5704/23332/23141/2491/5689/79980/5688/79902/10726/8881/5709/55839/8697/5707/79023/55166/5683/246184/5708/55055/6396/5692/80152/10213/5903/10197/54908/5518/55746/5682/1060/5684/4928/5693/9861/5718/5714/5705/7314/9183/23279/5687/91687/5905/23421/81929/5347/10393/401541/5702/5686/64682/2010/6233/9735/5706/5721/7443/5719
1296072	Voltage gated Potassium channels	44	0,4721	1,7408	0,0039	0,0064	131096/3747/3739/169522/3787/90134/9312/27012/3755/27133/3786/26290/26251/56479/93107/3790/3750/3745/81033/170850/3744/23415
429914	Deadenylation-dependent mRNA decay	48	-0,5453	-2,0343	0,0040	0,0064	23019/246175/4850/9924/55802/11340/196513/5393/10605/22894/57819/27258/9125/1981/23644/11157/27257/57472/1975/23016/25904/23404/51013/9337/167227/1974/9775/23658/51010
72702	Ribosomal scanning and start codon recognition	48	-0,5457	-2,0357	0,0040	0,0064	8668/1965/8664/8669/6194/6235/1981/8894/6187/8666/6227/6234/6201/8661/1975/8662/1983/6229/6189/6202/6228/1974/6233/6207/6192
72649	Translation initiation complex formation	48	-0,5398	-2,0140	0,0040	0,0064	8668/1965/8664/8669/6194/6235/1981/8894/6187/8666/6227/6234/6201/8661/1975/8662/6229/6189/6202/6228/1974/6233/6207/6192
418555	G alpha (s) signalling events	92	0,3801	1,6240	0,0040	0,0064	5741/116/2696/5151/5139/4159/155/113091/5744/5142/154/551/3360/2691/151306/2488/1812/10266/111/1392/799/50940/59350/133/2641/7252/2642/5746/5141/5739/2740/4158/7432/117579/5732/114/2492/2695
68875	Mitotic Prophase	69	-0,4651	-1,8377	0,0040	0,0064	5925/79902/10762/9631/9972/5861/53371/8359/79023/91754/2029/8086/10051/8615/6418/9818/23511/891/23175/23133/5903/55746/23310/4928/129401/23165/5578/8480/23279/81929/5347/64689/2010/10783/255919/7443/9133
72662	Activation of the mRNA upon binding of the cap-binding complex and eIFs, and subsequent binding to 43S	49	-0,5347	-1,9915	0,0040	0,0064	8668/1965/8664/8669/6194/6235/1981/8894/6187/8666/6227/6234/6201/8661/1975/8662/6229/6189/6202/6228/1974/6233/6207/6192
5250941	Negative epigenetic regulation of rRNA expression	63	-0,4702	-1,8306	0,0040	0,0064	221830/2068/8819/2965/64425/8359/7270/64426/23411/3066/5434/8350/2071/5437/9014/2968/8357/51742/5435/5440/84172/23378/29115/25885/9015/3021/51082/1022/9533/30834
381038	XBP1(S) activates chaperone genes	47	-0,5336	-1,9668	0,0041	0,0064	10954/1639/11015/10130/51726/55738/2673/9820/10106/9570/7466/5611/37/7094/9114/116138/6734/30827/55176/27230/1200/4189/58477/23480/64061
72695	Formation of the ternary complex, and subsequently, the 43S complex	42	-0,5341	-1,8938	0,0043	0,0068	3646/8668/1965/8664/8669/6194/6235/8894/6187/8666/6227/6234/6201/8661/8662/6229/6189/6202/6228/6233/6207/6192
389958	Cooperation of Prefoldin and TriC/CCT in actin and tubulin folding	26	-0,5826	-1,8646	0,0044	0,0069	81027/7277/84790/6950/5204/7203/10574/10576/10471/7411/10694/908/22948/10383/10575/5203/347733
212165	Epigenetic regulation of gene expression	78	-0,4447	-1,8048	0,0059	0,0092	221830/2068/8819/5928/2965/64425/8359/6996/7270/64426/23411/3066/5434/2146/200424/8350/3720/5931/2071/5437/9014/2968/8357/51742/5435/5440/84172/23378/29115/25885/9015/3021/51082/1022/9533/30834
5632684	Hedgehog 'on' state	78	-0,4406	-1,7882	0,0059	0,0092	5685/5694/11127/409/5691/79577/5700/8650/5690/84455/5695/5727/64750/5716/5717/5704/1452/5689/5688/5709/5707/5683/5708/5692/10213/2735/10197/25989/5682/5684/5693/9861/9978/5718/5714/5705/7314/5687/5702/5686/6233/5706/5721/5719
211000	Regulatory RNA pathways	84	-0,4139	-1,7070	0,0060	0,0092	5438/4927/79902/10762/9631/9972/53371/8359/79023/8086/7247/9818/23511/5434/5903/8350/55746/57510/5437/122402/11022/4928/129401/23165/8357/8480/4603/23279/5435/5440/5901/113802/5432/81929/5436/3021/55124
381070	IRE1alpha activates chaperones	50	-0,5149	-1,9282	0,0061	0,0093	10113/7494/9695/7095/84447/10954/1639/11015/10130/51726/55738/2673/9820/10106/9570/7466/5611/37/7094/9114/3309/116138/6734/30827/55176/27230/1200/4189/58477/23480/64061
5628897	TP53 Regulates Metabolic Genes	52	-0,4658	-1,7515	0,0061	0,0093	7531/27244/27165/51422/9167/2821/1327/54205/50484/5571/7157/1351/2936/1329/1350/25824/1349/6009/54541
3700989	Transcriptional Regulation by TP53	52	-0,4658	-1,7515	0,0061	0,0093	7531/27244/27165/51422/9167/2821/1327/54205/50484/5571/7157/1351/2936/1329/1350/25824/1349/6009/54541

5358351	Signaling by Hedgehog	109	-0,4085	-1,7574	0,0061	0,0093	6868/5575/84447/5713/5685/5694/11127/409/5691/79577/5700/5573/8650/5690/84455/5695/10956/5727/85455/64750/5716/5717/5704/1452/9742/5689/5576/5688/79809/5709/5707/57728/6500/51009/5683/5708/5692/10213/2735/10197/7415/8454/25989/5682/5684/5693/9861/9978/5718/5714/5705/7314/5687/5702/5034/5686/57539/2262/6233/5706/5721/5719
180910	Vpr-mediated nuclear import of PICs	29	-0,5397	-1,7623	0,0066	0,0099	4927/79902/10762/9631/9972/53371/79023/8086/9818/23511/5903/55746/4928/129401/23165/8480/23279/81929
390466	Chaperonin-mediated protein folding	27	-0,5621	-1,8138	0,0067	0,0101	81027/7277/84790/6950/5204/7203/10574/10576/10471/7411/10694/908/22948/10383/10575/5203/347733
419037	NCAM1 interactions	38	0,4775	1,7056	0,0075	0,0112	6900/1287/2674/1298/1285/1299/9048/8128/375790/1291/775/8912/1292/8911/8913/4902/1297/1286/50509/1280/5621
937061	TRIF-mediated TLR3/TLR4 signaling	74	-0,4054	-1,6310	0,0078	0,0116	5604/5594/23118/6885/2002/7323/5595/7187/29110/329/4790/6197/8737/1432/5608/5970/3551/7314/3661/1147/5528/1848/9252/929/6233/7099/4792/2353
2467813	Separation of Sister Chromatids	148	-0,3641	-1,6495	0,0078	0,0116	5685/29882/5694/7324/5691/5700/51434/5690/5695/5716/5717/5704/23332/2491/5689/79980/5688/79902/10726/8881/5709/55839/8697/5707/79023/55166/5683/246184/5708/55055/6396/5692/80152/10213/5903/10197/54908/55746/5682/1060/5684/4928/5693/9861/5718/5714/5705/7314/9183/23279/5687/91687/5905/23421/81929/5347/10393/401541/5702/5686/64682/6233/9735/5706/5721/5719
69275	G2/M Transition	106	-0,3898	-1,6821	0,0080	0,0118	1639/80184/4659/5116/8481/7283/23332/7277/7531/10844/4957/4331/27229/286826/5928/23291/27175/85378/9088/10426/6500/11258/80321/55142/11116/891/80254/5518/132660/79959/8454/9662/8655/11064/22995/7314/5347/890/3320/55957/10383/6233/7465/6790/1022/9133
5578749	Transcriptional regulation by small RNAs	59	-0,4484	-1,7347	0,0080	0,0118	4927/79902/10762/9631/9972/53371/8359/79023/8086/9818/23511/5434/5903/8350/55746/5437/4928/129401/23165/8357/8480/23279/5435/5440/5901/5432/81929/5436/3021
427413	NoRC negatively regulates rRNA expression	60	-0,4579	-1,7791	0,0081	0,0118	221830/2068/8819/2965/64425/8359/7270/64426/3066/5434/8350/2071/5437/9014/2968/8357/51742/5435/5440/84172/29115/25885/9015/3021/51082/1022/9533/30834
156580	Phase II conjugation	57	-0,4722	-1,7995	0,0082	0,0120	27306/6799/10249/27430/6817/7172/9446/4144/9060/2950/7360/79017/51000/1836/7358/2947/4257/2944/2949
3214847	HATs acetylate histones	107	-0,3722	-1,6092	0,0099	0,0143	23587/9913/8345/55167/7994/93624/8518/10943/56943/26610/8520/8359/10856/9643/2648/3017/79960/9575/1810/11143/5077/54556/8350/64769/5931/8349/11091/55140/55250/80314/6944/10902/6883/8357/86/27097/112869/84289/84148/54934/8607/8342/8347
1592230	Mitochondrial biogenesis	29	-0,5348	-1,7460	0,0110	0,0158	56652/133522/5564/51422/211/54205/5571/1432/2551/506/2553/64216/6742/23082/7978/11232/7019
176033	Interactions of Vpr with host cellular proteins	32	-0,5213	-1,7428	0,0111	0,0158	4927/79902/10762/9631/9972/53371/79023/8086/9818/23511/5903/55746/4928/129401/23165/8480/23279/81929/291
5601884	PIWI-interacting RNA (piRNA) biogenesis	27	-0,5433	-1,7533	0,0112	0,0159	5437/122402/11022/4603/5435/5440/113802/5432/5436/55124
5357801	Programmed Cell Death	152	-0,3427	-1,5564	0,0117	0,0166	637/5713/5685/7532/5694/5691/5700/7186/6093/5690/5695/7098/5716/8797/3146/5717/5704/3836/10134/7531/5689/5534/5688/5709/3837/5707/54205/329/5683/197259/5708/5692/10213/8737/355/7157/10197/4836/598/5682/22985/5684/8655/5693/9861/5718/5714/5705/7314/5687/5702/5686/929/90249/79658/6233/9414/5706/5721/5719/7431/7099/1832
453274	Mitotic G2-G2/M phases	108	-0,3713	-1,6005	0,0122	0,0173	1639/80184/4659/5116/8481/7283/23332/7277/7531/10844/4957/4331/27229/286826/5928/23291/27175/85378/9088/10426/6500/11258/80321/55142/11116/891/80254/5518/132660/79959/8454/9662/8655/11064/22995/7314/5347/890/3320/55957/10383/6233/7465/6790/1022/9133

373080	Class B/2 (Secretin family receptors)	41	0,4383	1,6068	0,0130	0,0183	5741/116/2696/113091/5744/8325/2691/10266/7855/1392/799/7471/8322/133/2641/2642/5746/2740/6608
3560782	Diseases associated with glycosaminoglycan metabolism	26	-0,5549	-1,7758	0,0132	0,0184	2239/63827/2262/2331/6385/1634/4958/4969
3781865	Diseases of glycosylation	26	-0,5549	-1,7758	0,0132	0,0184	2239/63827/2262/2331/6385/1634/4958/4969
109581	Apoptosis	149	-0,3403	-1,5424	0,0136	0,0188	637/5713/5685/7532/5694/5691/5700/7186/6093/5690/5695/7098/5716/8797/3146/5717/5704/3836/10134/7531/5689/5534/5688/5709/3837/5707/54205/329/5683/5708/5692/10213/8737/355/7157/10197/4836/598/5682/22985/5684/8655/5693/9861/5718/5714/5705/7314/5687/5702/5686/929/90249/79658/6233/9414/5706/5721/5719/7431/7099/1832
166166	MyD88-independent TLR3/TLR4 cascade	75	-0,3980	-1,6051	0,0136	0,0188	5604/5594/23118/6885/2002/7323/5595/7187/29110/329/4790/6197/8737/1432/5608/5970/3551/7314/3661/1147/5528/1848/9252/929/6233/7099/4792/2353
168164	Toll Like Receptor 3 (TLR3) Cascade	75	-0,3980	-1,6051	0,0136	0,0188	5604/5594/23118/6885/2002/7323/5595/7187/29110/329/4790/6197/8737/1432/5608/5970/3551/7314/3661/1147/5528/1848/9252/929/6233/7099/4792/2353
421270	Cell-cell junction organization	46	0,4177	1,5589	0,0138	0,0189	1006/53842/49861/137075/23562/1007/24146/5818/92359/1005/1004/64398/1000/1002/999/5819/1012/1009/1015/84552/81607
909733	Interferon alpha/beta signaling	58	-0,4377	-1,6756	0,0143	0,0195	3454/684/51447/3661/3659/25939/3664/2634/10379/3394/10410/8519/1958/10581
2565942	Regulation of PLK1 Activity at G2/M Transition	78	-0,3912	-1,5876	0,0158	0,0215	55125/79866/10142/7840/5048/1453/7532/10121/1639/80184/4659/5116/8481/7283/23332/7277/7531/4957/23291/6500/11258/80321/55142/11116/891/80254/5518/79959/8454/9662/8655/11064/22995/7314/5347/3320/10383/6233/6790/9133
114608	Platelet degranulation	77	-0,3891	-1,5781	0,0177	0,0239	7094/7414/3309/5660/29106/967/813/5627/6647/5265/2335/1191/5175/2316/5341/6678/7450/7076/710/7057/5552/7422/7042/2162
168898	Toll-Like Receptors Cascades	105	-0,3528	-1,5207	0,0180	0,0242	5604/5594/23118/7334/6885/2002/7323/30849/5595/7187/29110/329/4790/1508/6197/8737/1432/5608/3689/5970/3551/7314/1513/3661/1147/5528/1848/9252/389898/929/7097/1514/6233/1520/7099/4792/2353
70153	Glucose transport	38	-0,4879	-1,6960	0,0193	0,0259	79902/10762/9631/9972/53371/3098/79023/8086/9818/23511/5903/55746/6515/4928/129401/23165/8480/23279/81929
3858494	beta-catenin independent WNT signaling	112	-0,3518	-1,5257	0,0198	0,0264	801/5713/5685/5694/409/5691/5700/5532/3709/5530/5690/5695/192669/64750/161/5716/5717/5704/5216/6259/5689/5534/5688/815/6885/5709/5707/5683/6934/5708/5692/10213/805/1856/10197/160/5682/5684/54331/5693/5578/9861/5718/5714/5705/7314/5687/5702/5686/5879/6233/7474/5706/5721/5719
166058	MyD88:Mal cascade initiated on plasma membrane	70	-0,3850	-1,5317	0,0217	0,0285	5594/23118/7334/6885/2002/5595/4790/6197/1432/5608/5970/3551/7314/1147/5528/1848/9252/389898/929/7097/6233/7099/4792/2353
181438	Toll Like Receptor 2 (TLR2) Cascade	70	-0,3850	-1,5317	0,0217	0,0285	5594/23118/7334/6885/2002/5595/4790/6197/1432/5608/5970/3551/7314/1147/5528/1848/9252/389898/929/7097/6233/7099/4792/2353
168179	Toll Like Receptor TLR1:TLR2 Cascade	70	-0,3850	-1,5317	0,0217	0,0285	5594/23118/7334/6885/2002/5595/4790/6197/1432/5608/5970/3551/7314/1147/5528/1848/9252/389898/929/7097/6233/7099/4792/2353
168188	Toll Like Receptor TLR6:TLR2 Cascade	70	-0,3850	-1,5317	0,0217	0,0285	5594/23118/7334/6885/2002/5595/4790/6197/1432/5608/5970/3551/7314/1147/5528/1848/9252/389898/929/7097/6233/7099/4792/2353
166054	Activated TLR4 signalling	88	-0,3735	-1,5534	0,0220	0,0287	5604/5594/23118/7334/6885/2002/7323/5595/7187/29110/329/4790/6197/8737/1432/5608/5970/3551/7314/3661/1147/5528/1848/9252/389898/929/7097/6233/7099/4792/2353
73886	Chromosome Maintenance	67	-0,3906	-1,5472	0,0220	0,0287	2491/5985/5928/1763/55839/5111/8359/5427/7013/55166/80152/5558/65057/5931/26277/1736/1060/4869/10714/91687/54386/23421/401541/54069/5982/328/8607/55320/5984/5426
76005	Response to elevated platelet cytosolic Ca2+	82	-0,3774	-1,5401	0,0222	0,0288	7094/7414/3309/5660/29106/967/813/5627/5578/6647/5265/2335/1191/5175/2316/5341/6678/7450/7076/710/7057/5552/7422/7042/2162

5620912	Anchoring of the basal body to the plasma membrane	86	-0,3708	-1,5446	0,0236	0,0304	55125/54903/10142/7840/5048/347240/79600/1453/7532/10121/1639/132320/80184/5116/261734/8481/26005/7283/23332/117177/7277/7531/4957/11258/9657/22832/80321/55142/11116/79867/80254/5518/79959/8766/9662/51259/8655/11064/22995/91147/84902/5347/3320/10383/26123
211897	Cytochrome P450 - arranged by substrate type	49	0,4014	1,5146	0,0237	0,0304	1591/1558/8529/1585/1543/1576/56603/1572/1586/1555/1592/9420/66002/1544/1557/1594/1580/6916/1595/1588/126410/5740
2022090	Assembly of collagen fibrils and other multimeric structures	39	0,4244	1,5267	0,0244	0,0312	85301/1287/3918/7093/1285/4016/1288/1295/255631/3909/1291/1300/1292/3914/1286/50509/1280/1296/80781/1308/667
1500931	Cell-Cell communication	118	0,3143	1,4122	0,0252	0,0321	1006/8935/1630/3918/53842/84063/49861/7827/88/2318/137075/84623/9423/57453/23562/1007/3852/24146/7305/8826/5818/3909/7408/89/92359
1474228	Degradation of the extracellular matrix	103	0,3169	1,3763	0,0279	0,0354	2/1287/3918/7093/3912/1515/4318/7077/256076/4312/1298/4327/1215/1285/9507/1299/5340/1288/1295/3909/9313/1291/4313/1300/84570/91522/1292/1991/826/176/1297/3914/1286/4324/6696/5650/50509/131873/1280/999/1511
1296071	Potassium Channels	96	0,3297	1,4216	0,0280	0,0354	131096/2786/3759/3747/3739/169522/348980/3787/90134/9312/9424/610/27012/3755/2792/27133/10242/89822
199991	Membrane Trafficking	136	-0,3193	-1,4279	0,0296	0,0372	10113/6456/26258/9146/1315/8729/11267/91782/372/10484/9698/9847/11336/11316/9525/808/801/7532/11127/162/8027/27183/27243/9276/55738/8907/8906/84062/134829/5564/7531/137492/3312/10640/51422/22818/149371/8775/9632/164/51028/10717/1211/23216/6396/5571/23431/805/2706/11154/54536/51552/8773/208/7251/51510/9685/5872/7314/84313/10802/2512/2495/6233/1601/2697
2672351	Stimuli-sensing channels	85	0,3378	1,4139	0,0305	0,0383	7225/22802/56302/140803/7439/9635/79054/1184/63982/121601/203859/4308/59341/10110/10345/8989/7809/338440/7224/285335/259232/55283/162514/54795/6337/7222/1179/41/55503/10786/11059/55107/6262/1180
166016	Toll Like Receptor 4 (TLR4) Cascade	91	-0,3649	-1,5318	0,0319	0,0398	5604/5594/23118/7334/6885/2002/7323/5595/7187/29110/329/4790/6197/8737/1432/5608/3689/5970/3551/7314/3661/1147/5528/1848/9252/389898/929/7097/6233/7099/4792/2353
73923	Lipid digestion, mobilization, and transport	43	0,3936	1,4525	0,0340	0,0422	3949/646486/2169/81693/3991/64240/2170/345/5568/4018/336/337/5408/29881
189200	Hexose transport	40	-0,4625	-1,6253	0,0341	0,0422	79902/10762/9631/9972/53371/3098/79023/8086/9818/23511/5903/55746/6515/4928/129401/23165/8480/23279/81929
5620924	Intraflagellar transport	37	-0,4683	-1,6183	0,0343	0,0422	112752/26160/83657/83658/56912/9321/11020/64792/11127/8100/9742/79809/57728/51626/51668/55112/90410/79659/8655/55081/51098/79989/57539/23059/80173
2559583	Cellular Senescence	128	-0,3163	-1,4062	0,0358	0,0439	6667/5594/8881/5928/5595/8697/8359/7290/7013/10919/4790/6015/79813/246184/6197/7157/8550/2146/65057/1432/8350/5608/5931/4683/3569/29855/25842/26277/5970/3576/8357/7314/54386/10393/64682/4361/6233/3021/1027/1019/2114/2353
975138	TRAF6 mediated induction of NFkB and MAP kinases upon TLR7/8 or 9 activation	64	-0,3750	-1,4681	0,0363	0,0444	5594/23118/7334/6885/2002/5595/4790/6197/1432/5608/5970/3551/7314/1147/5528/1848/9252/389898/6233/4792/2353
425397	Transport of vitamins, nucleosides, and related molecules	35	0,4244	1,4950	0,0383	0,0466	6579/28965/28234/3933/28232/11309/10599/551/11046

**setSize**	**|**	**Number of genes on the pathway**
**enrichmentScore**	**|**	**Enrichment score**
**NES**	**|**	**Normalized enrichment score**
**qvalues**	**|**	**q-value**
**pvalue**	**|**	**p-value**
**coreEnrichment**	**|**	**Core enrichment for the pathway (Entrez IDs).**

Notes	|	log2 (fold changes) computed with limma R package between the groups
Notes	|	(HHV-6 infected vs. noninfected) with diagnosis/no diagnosis, age, and
Notes	|	post-mortal interval (PMI) as covariates.
Notes	|	seed = 20
Notes	|	set size >= 25 & >=200
Notes	|	ReactomePA R package 1.18.1, R version 3.3.2
Notes	|	Highlighted in green = pathways associated with viral infection
Notes	|	Highlighted in red = pathways associated with Toll-like receptor signaling
